# Efficacy of a four-curvature auxiliary arch at preventing maxillary central incisor linguoclination during orthodontic treatment: a finite element analysis

**DOI:** 10.1186/s12903-023-02833-2

**Published:** 2023-03-11

**Authors:** Ping-Zhu Yang, Li-Yun Bai, He-Xuan Zhang, Wen-Jun Zhao, Yu liu, Xiu-Jie Wen, Rui Liu

**Affiliations:** 1 Department of Stomatology, Daping Hospital, Third Military Medical University (Army Medical University), Chongqing, 400042 China; 2 Department of Orthodontics, School of Dentistry, Southwest Medical University, Luzhou, 646000 China; 3 ChuangNeng Technology (ChongQing) Co. LTD, Chongqing, 400042 China; 4 State Key Laboratory of Trauma, Burns and Combined Injury, Wound Trauma Medical Center, Daping Hospital, Third Military Medical University (Army Medical University), Chongqing, 400042 China

**Keywords:** Microimplant, Four-curvature auxiliary arch, Three-dimensional finite element, Maxillary incisors, Torque

## Abstract

**Background:**

Correct torque of the incisors is beneficial in the assessment of the effects of orthodontic treatment. However, evaluating this process effectively remains a challenge. Improper anterior teeth torque angle can cause bone fenestrations and exposure of the root surface.

**Methods:**

A three-dimensional finite element model of the maxillary incisor torque controlled by a homemade four-curvature auxiliary arch was established. The four-curvature auxiliary arch placed on the maxillary incisors was divided into four different state groups, among which 2 groups had tooth extraction space retracted traction force set to 1.15 N. Initial displacements and pressure stresses of the periodontal tissue in the maxillary incisors and molars were calculated after torque forces (0.5, 1, 1.5, and 2 N) were applied to the teeth at different stable states.

**Results:**

The effect of using the four-curvature auxiliary arch on the incisors was significant but did not affect the position of the molars. Given the absence of tooth extraction space, when the four-curvature auxiliary arch was used in conjunction with absolute anchorage, the recommended force value was < 1.5 N. In the other 3 groups (i.e., molar ligation, molar retraction, and microimplant retraction groups), the recommended force value was < 1 N. The application of a four-curvature auxiliary arch did not influence the molar periodontal and displacement.

**Conclusion:**

A four-curvature auxiliary arch may treat severely upright anterior teeth and correct cortical fenestrations of the bone and root surface exposure.

**Supplementary Information:**

The online version contains supplementary material available at 10.1186/s12903-023-02833-2.

## Introduction

Good torque control of the maxillary incisors is very important. Incorrect torque influences orthodontic treatment, thereby affecting aesthetic outcomes and occlusal relationship stability. For example, when the maxillary incisors are severely upright, the root of the maxillary incisors can pierce the last line of defense of the alveolar bone. This condition eventually causes bone fenestrations and results in the root without a foothold [1–3]. Experimental studies on the movement of teeth during orthodontic treatment are based on the ability of the alveolar bone and periodontal ligament to respond to mechanical stimuli, which generate force during a loading process, and the strain action causes tooth movement [4–6]. The combined action of multiple orthodontic forces during an orthodontic process had been shown to cause abnormal torque movement of the teeth. If a large periodontal stress is generated at the root apex of a tooth, tooth root absorption may occur, which is an undesirable histological reaction [7, 8].

Arch wire stiffness refers to the inherent rigidity determined by the shape and area of the cross section of the material. Orthodontists can change the stiffness of the whole or local orthodontic appliance by selecting different materials of the arch wire, various cross-section sizes, and designing distinct spans of the arch wire and the configuration of the bend to accurately control the orthodontic force [9–11]. To achieve good control of the maxillary incisor torque angle during the correction process, we proposed a new type of torque auxiliary arch device: a homemade four-curvature auxiliary arch, which is used in orthodontics treatment (Patent No: ZL 201420113873.9). The four-curvature auxiliary arch is used on the gingival end of each incisor bracket to generate a root–tongue and crown lip–directed root-control movement of the maxillary incisor to correct the axial tilt of the tongue. The shaft is returned to or near its normal position. Contrary to the traditional auxiliary arch, it arch can only be changed by the transmission of the connecting part. In this study, in the four-curvature auxiliary arch, the moment arm could be infinitely close to the tooth cervical, at which the pressure could be directly applied. The longer the torque arm was, the closer it was to the impedance center of the tooth, and the higher the torque efficiency was. In addition, compared with the traditional five-piece auxiliary arch, the four-curvature auxiliary arch was smaller and more beautiful, which were conducive to the maintenance of oral hygiene [12].

Finite element analysis involves quantitative evaluation and intuitive and correct image expression and analysis. The orthodontic force involved in initial tooth displacement, associated size and direction of the teeth, tooth root and periodontal membrane characteristics, and periodontal membrane hydrostatic pressure values were evaluated. The displacement values of the incisors were extracted from the incisor end of the crown and the incisor root tip, which facilitated the analysis of variation in the tooth movements and assessment of the risk of periodontal membrane necrosis and root resorption [13]. Therefore, this study was performed to use finite element analysis to explore whether the effect of the four-curvature auxiliary arch was obvious and if the root torque control movement could be carried out by stabilizing the main bow wire at different states. Whether the application of microimplants could better assist the auxiliary arch to exert torque force was also determined. The results could provide a better basis for clinical application.

## Materials and methods

### Four-curvature materials and methods

**Fig. 1:**
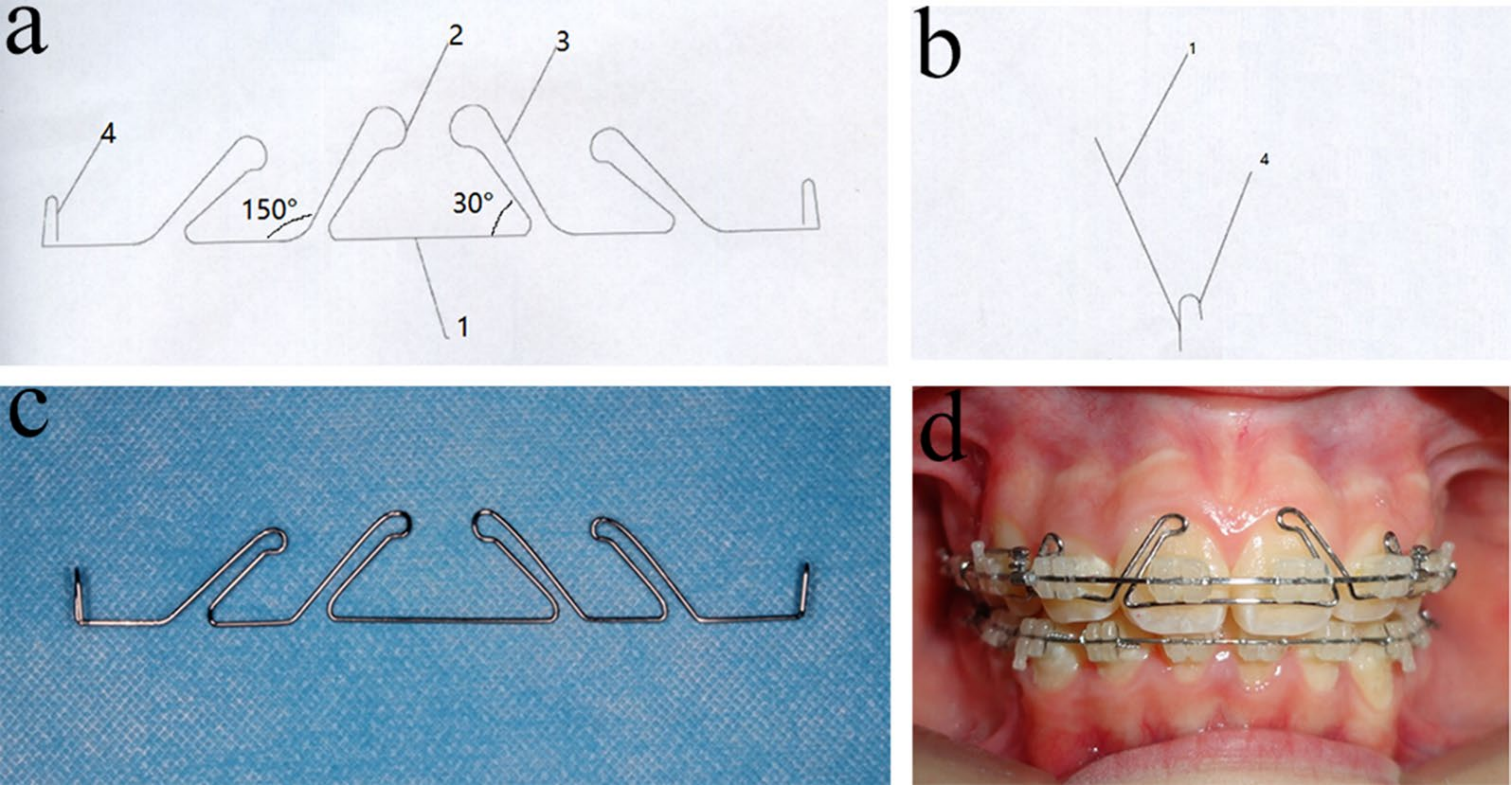
Four-curvature of the process

**Fig. 2:**
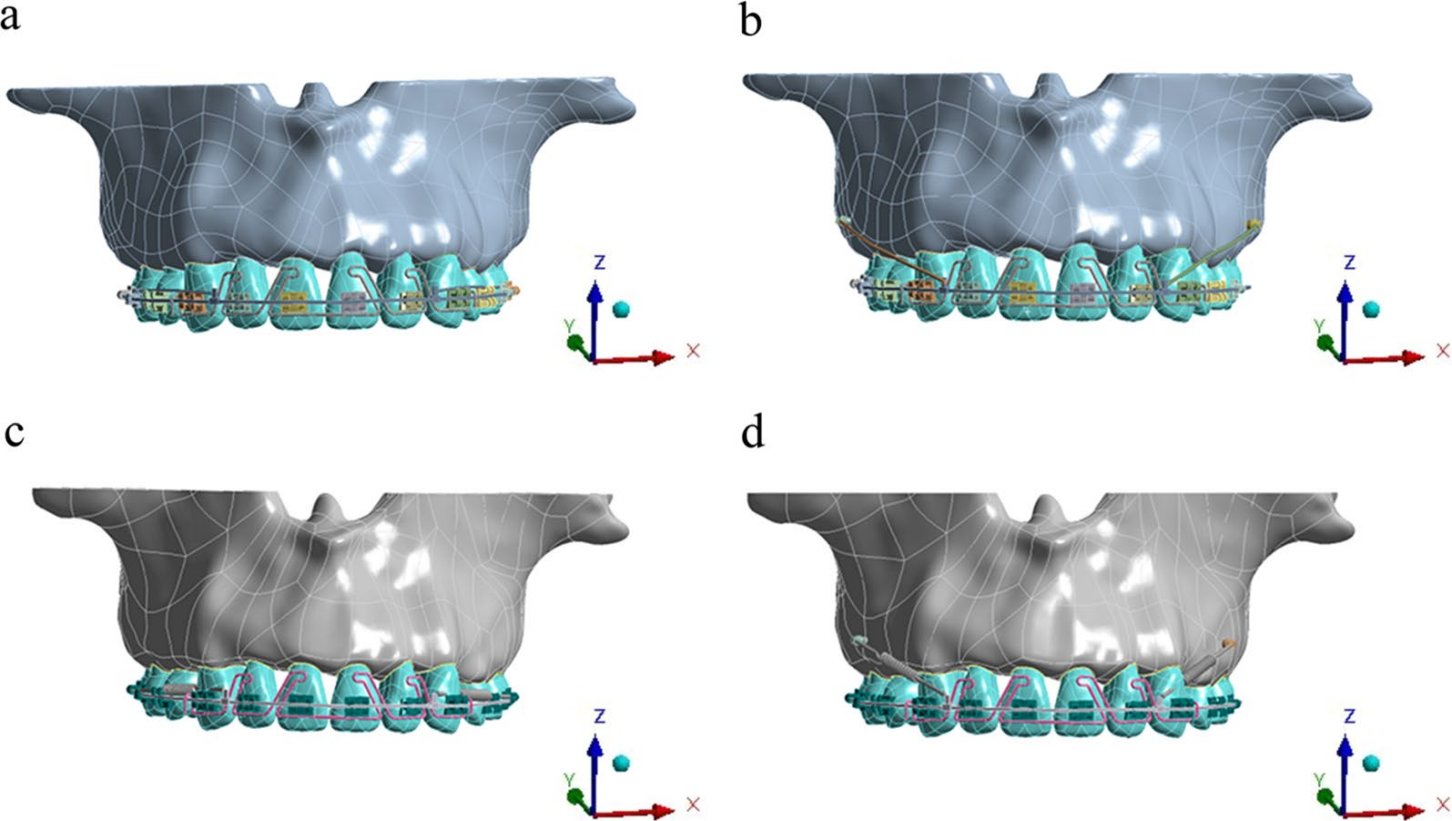
Four kinds of 3D FE models. **a** Molar ligation group. **b** Micro-implant ligation group. **c** Micro-implant retraction group. **d** Micro-implant ligation group

**Fig. 3:**
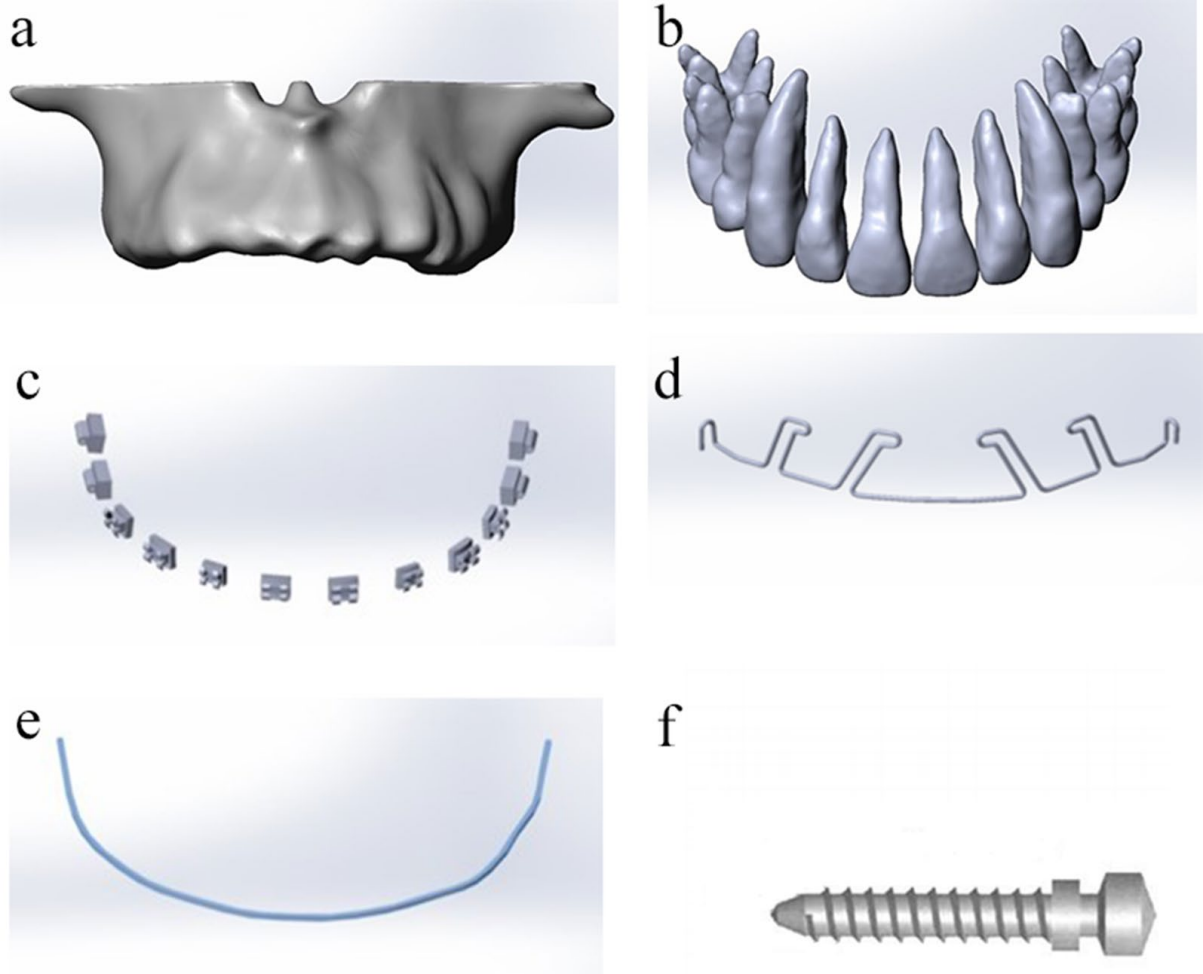
Finite element smooth model

**Fig. 4:**
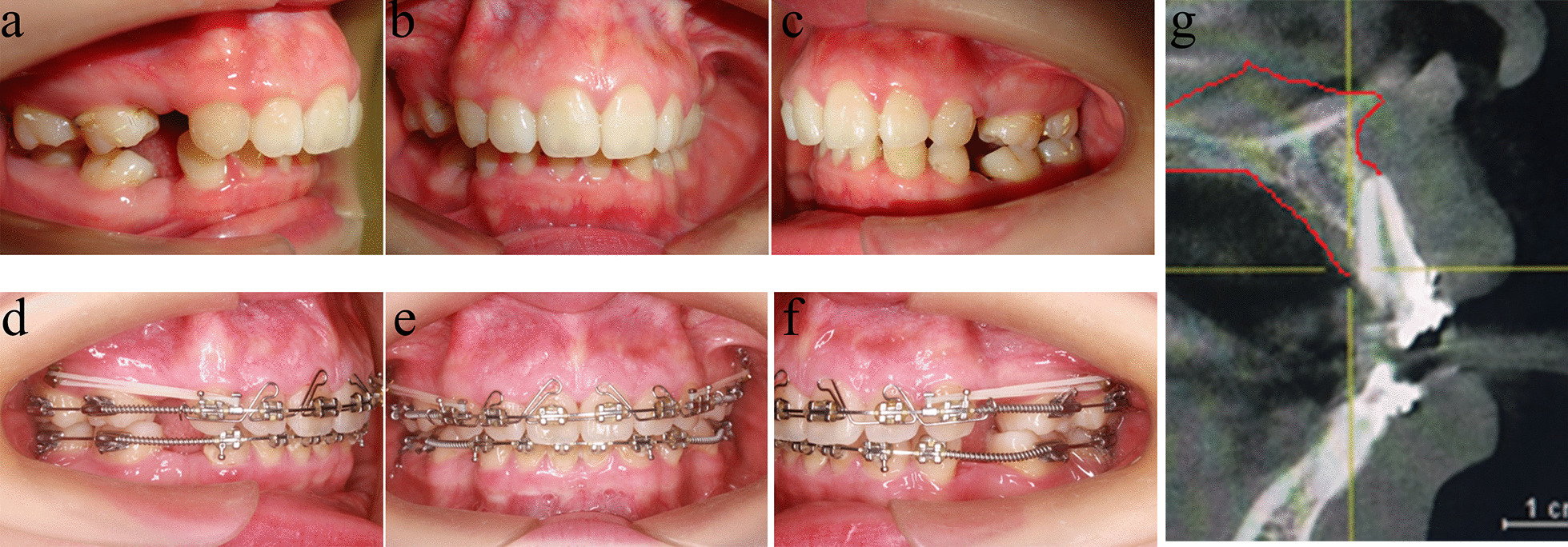
Intraoral photographs & CBCT before and during treatment

A homemade four-curvature auxiliary arch was made of Australian arch wire with a diameter of 0.457 mm (The Fordrough, HayMills, Birmingham B25 8DW, UK). Australian arch wire was selected because of its bow wire characteristics, that is, the balance between hardness and elasticity and slow stress attenuation. First, four curve arms were bended, and the four curves were inclined from the distal to proximal side and reached the midpoint of the labial face and cervical of the teeth. When in use, the formed base of the auxiliary arch was bent in the circumferential direction pointing to the curving process at approximately 150° (Figs. 1a, 2). The four spatulate curving processes were inclined to the palatal side and pressed at approximately 30° (Figs. 1a, 3). Two hooks were bent in the distal position of the two sides of the maxillary canine and hung on the main arch wire for auxiliary arch retention (Figs. 1b, 4). When the auxiliary arch was placed on the dentition, from the maxillary central incisors through the lateral incisors and maxillary canine (Figs. 1b, 4), the four-curvature auxiliary arch was gradually placed on the teeth, and the hook was attached to the main arch wire from the inside out. Thus, four long arch-shaped processes exerting forces were carried out. The auxiliary arch was placed upward on the arcuate wire from the bottom of the anterior denture groove. The four long arch-shaped processes reached the position of the tooth cervical from far to near and close to the center of impedance (Fig. 1c, d).

### Finite element sample collection

A 19-year-old female volunteer without dental caries, no periodontal disease or no systemic diseases, no crowding, no spacing, normal inclination, and anterior overbite, and a class I molar relationship was selected. The patient signed informed consent and confirmed that she volunteered all information/images of her clinical treatment in stomatology to the Stomatology Department of the Army Specialty Medical Center.

### CBCT taking

The CT scanning data were imported into Mimics 19.0 software for image processing. The conditions were as follows: CT exposure timing of 14/2–6 s, kVp 85, mA 5–7, spatial resolution of 150–300 μm, field of view (FoV) of 15 cm × 15 cm × 15 cm, CT slice thickness of 0.2 mm, and space requirement of 1800 × 1800 × 2500.

The images of the maxillary bone and teeth were taken with a CBCT scanner (Sirona Dental, GALILEOS, Germany), saved in the form of digital imaging and communication in medicine (DICOM) data, and exported. The scanning time was 14 s, and 210 single-tooth images were taken. The scanning was conducted from the lower orbital margin to the chin. After scanning, 733 CT images were obtained, and the CT scan data were output in DICOM format.

### Modeling the original 3D volume from CBCT

The DICOM data of the patient’s teeth and alveolar bone were obtained, and the cross-sections were converted into a 3D mathematical model by using MIMICS 19.0 (Materialise, Leuven, Belgium), exported to Geomagic Studio (Geomagic Company, NC, USA), and modified by Solidworks and 3-matic research. The 3D geometric parameters of the maxilla, including the cortical and cancellous bones, teeth and periodontal membrane characteristics, were obtained. The bracket 0.022 (0.5588 × 0.7112 mm) MBT system bracket ceramic bracket and buccal tube, microimplant (2 ORMCO vectors TAS with an intrabone length of 8 mm), stainless steel wire (0.483 × 0.635 mm, TP Orthodontics, Inc., USA), and orthodontic wire (stainless steel wire with a diameter of 0.25 mm) were designed by SolidWorks^®^ 12.0 (SolidWorks Corporation, Velizy-Villacoublay, France). Finally, a smooth geometric model was generated.

The maxillary parts that need to be separated are located through the view and the threshold and contrast were adjusted to carry out regional growth. The selected images were segmented and refined. The 3D toolbar was used to generate a 3D model, which can display the diagram of the maxillary bone and save it in TXT point cloud format. The images of single maxillary tooth were sequentially separated. The obtained 3D images of the maxillary bone and teeth were output to the reverse-engineering software Geomagic in the point cloud format to remove noise, trim the edge shape, and generate a smooth solid model after automatic surface optimization. According to the thickness of the clinical periodontal membrane, the whole tooth root was uniformly extended by 0.2 mm to the periphery by using the migration command to obtain the precursor model of the membrane. The 3D solid model of the periodontal membrane was obtained by Boolean subtraction with the dental model. The models of maxilla, tooth, and periodontal membrane were exported in the IGES format.

### Material properties

The elastic modulus and Poisson’s ratio of the different materials are shown in Table 1 [13–15], and details on the FEM model can be found in the literature.

**Table 1:** Mechanical properties

Material	Poisson's ratio	Elastic modulus (MPa)
Teeth	0.3	20.7 × 10^3^
Cortical bone	0.3	13.7 × 10^3^
Cancellous bone	0.3	1.37 × 10^3^
Periodontal membrane	0.45	68.9
Bracket	0.3	20.6 × 10^4^
Four-curved auxiliary arch	0.3	17.6 × 10^4^
Bow wire	0.3	17.6 × 10^4^
Traction hook	0.3	17.6 × 10^4^
Micro-implant	0.35	10.34 × 10^4^

### Meshing

The proposed model was meshed with 4-node tetrahedral-like elements, material attributes were set (the unit meshed was divided into units with area of 1 mm^2^), contact relations were set. The monomer results of the model were as follows: maxillary cancellous bone, maxillary cortical bone, teeth, periodontal membrane, bracket, bow wire, and micro-implants. The total numbers of elements and nodes are listed in Table 2.

**Table 2:** The number of elements and nodes

Material	Nodes	Elements
Teeth	63,189	10,406
Cortical bone	34,098	44,075
Cancellous bone	33,577	17,924
Periodontal membrane	65,096	20,433
Bracket	13,435	14,078
Bow wire	1248	553
Micro-implant	15,492	9103

### Creating sub-models from the original 3D model

Different torque force values (0.5, 1, 1.5, and 2 N) were applied to the cervical aspect of the incisor buccal side (Fig. 2) in Table 3.

**Table 3:** Model group

There is no gap in or back way		Different torque forces (N)			
		0.5	1	1.5	2
*No tooth extraction space*					
Molar ligation group	(No implant)	1^a^	1^b^	1^c^	1^d^
Micro-implant ligation group	(Implant)	2^a^	2^b^	2^c^	2^d^
*There's a tooth extraction space*					
Molar retraction group	(No implant)	3^a^	3^b^	3^c^	3^d^
Micro-implant retraction group	(Implant)	4^a^	4^b^	4^c^	4^d^

The a, b, c, d respectively correspond to the (0.5, 1, 1.5, 2) N. Where ^a^ stands for 0.5 N, where ^b^ stands for 1 N, where ^c^ stands for 1.5 N, where ^d^ stands for 2 N

Model 1: molar ligation group (the first molar stabilizes the main arch wire and has no traction).

Model 2: micro-implant ligation group (the micro-implants stabilize the main arch wire without traction).

Model 3: molar retraction group (the first molar stabilizes the main arch wire, and the retracted traction force is set at 1.15 N).

Model 4: micro-implant retraction group (the micro-implants stabilize the main arch wire, and the retracted traction force is set at 1.15 N).

### Common characteristics of the sub-models

The root and periodontal membrane, the periodontal membrane and alveolar bone, the tooth and bracket, and the micro-implants and jaw bone had bonded connections. The orthodontic wire and bracket and four-curvature auxiliary arch and tooth were set as contact connections, with a friction coefficient of 0.3. A frictionless connection was set between the teeth (Table 4).

**Table 4:** Connection relation

Material	Connections
Root and periodontal membrane	Bonded connection
Micro-implants and jaw bone	Bonded connection
Periodontal membrane and alveolar bone	Bonded connection
Tooth and bracket	Bonded connection
Bow wire and the bracket	Contact connection
Four-curvature auxiliary arch and teeth	Contact connection
Between the teeth	Frictionless connection

### Loading or manipulation

Load was calculated, and Gaussian curvature was determined in the model. The material properties were set as follows: mandible as a heterogeneous and anisotropic linear elastic material; and periodontal membrane as a heterogeneous and anisotropic nonlinear elastic material. The teeth, brackets, arch wires, back-binding wires, and traction hooks were set as continuous, homogeneous, and isotropic linear elastic materials (Fig. 3). Torque force (0.5, 1, 1.5, and 2 N) was applied to the tooth neck by the four models simulating different stabilizing main arch wires, while 1.15 N traction was applied to the tooth extraction group. In the entire experiment, except for a small amount of displacement caused by the setting of the traction force, the rest were under deformation.

### Outcomes

The FEA simulation was static. The created and loaded models were compared in terms of displacement, von Mises stress, and hydrostatic stress PDLs of all the components. Several methods can be used to explain tooth displacement, and two of which were used in this study. Tooth movement can be described based on the displacement of each tooth and its bracket. The direction of the torque force applied to the incisors is exactly the same, indicating that an axis of local coordinates is drawn at the location of the bracket. Another method is the use of external references, such as a global coordinate system. The global coordinate includes three axes: anterior–posterior, up–down, and left–right [16]. PDL separately analyzed the situation of a single tooth from the local axis. The local axes were defined individually for each tooth. The vertical axis was defined as exactly the global (vertical) axis. The mesiodistal axis was defined as the axis pointing from the distal (negative) to the mesial (positive) part of each tooth. The buccolingual (or buccopalatal) axis was defined as the axis pointing from the buccal (negative) to the palatal (positive) part of each tooth.

### Optimal interval of PDL stress–strain

Liao et al. (2016) suggested that the hydrostatic stress of the PDL should be higher than the capillary blood pressure of 0.0047 N/mm^2^ but should not exceed the human systolic pressure of 16.0 kPa [17]. Individual differences, bone category, teeth distribution, and other complex environmental factors as well as the upper and lower limits of optimal hydrostatic stress may significantly vary in clinical situations. Therefore, compressive hydrostatic stresses at the PDLs were compared with—0.0047 N/mm^2^ as a threshold for a significant increase of the risk of external root resorption [18]. If the PDL hydrostatic pressure exceeds the capillary pressure in the area, then the vessels will collapse and blood flow to that area will be impaired, thereby increasing the risk of root resorption [19, 20].

### Four-curvature auxiliary arch application to clinical treatment

All clinical procedures were approved by the Ethical Committee of the Army Medical University and performed in accordance with the applicable guidelines. On July 8, 2015, a 24-year-old female was transferred to the Stomatology Department of the Military Medical Center requiring correction of the incisor protrusion (Fig. 4a–c). The patient signed informed consent and confirmed to have volunteered all information/images subjects of the clinical treatment in stomatology to the Stomatology Department of the Army Specialty Medical Center. The patient received orthodontic treatment (8 premolars were extracted) in a local dental clinic 1 year before consultation with the team, and the failure of anterior root control resulted in incisor lingual inclination. CBCT showed that the anterior tooth root was already outside the alveolar bone, resulting in bone fenestrations. Then, the patient requested to be transferred to our hospital for treatment. (Fig. 4d–g). She had bilateral full class II molar relationships with an overjet of 10.5 mm and skeletal class II. A deep overbite of about 8 mm was diagnosed, and the low incisors bit on the front palate. The left first premolar and second molar were positive in crossbite. Almost no crowd was found in both arches. No significant skeletal asymmetry or temporomandibular joint disease was detected.

## Results

### Displacement of the maxillary dentition

**Fig. 5:**
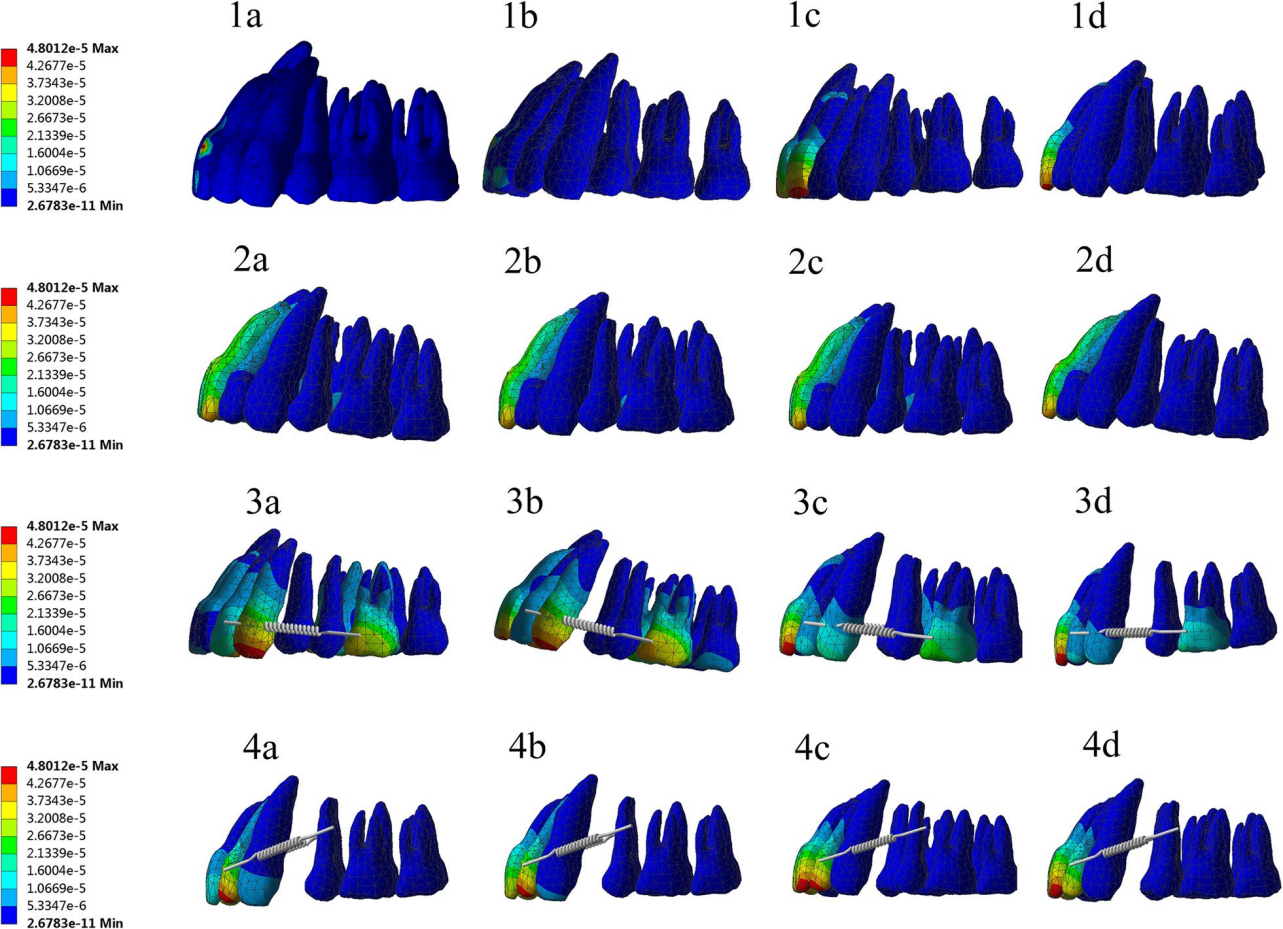
Displacement distribution

**Fig. 6:**
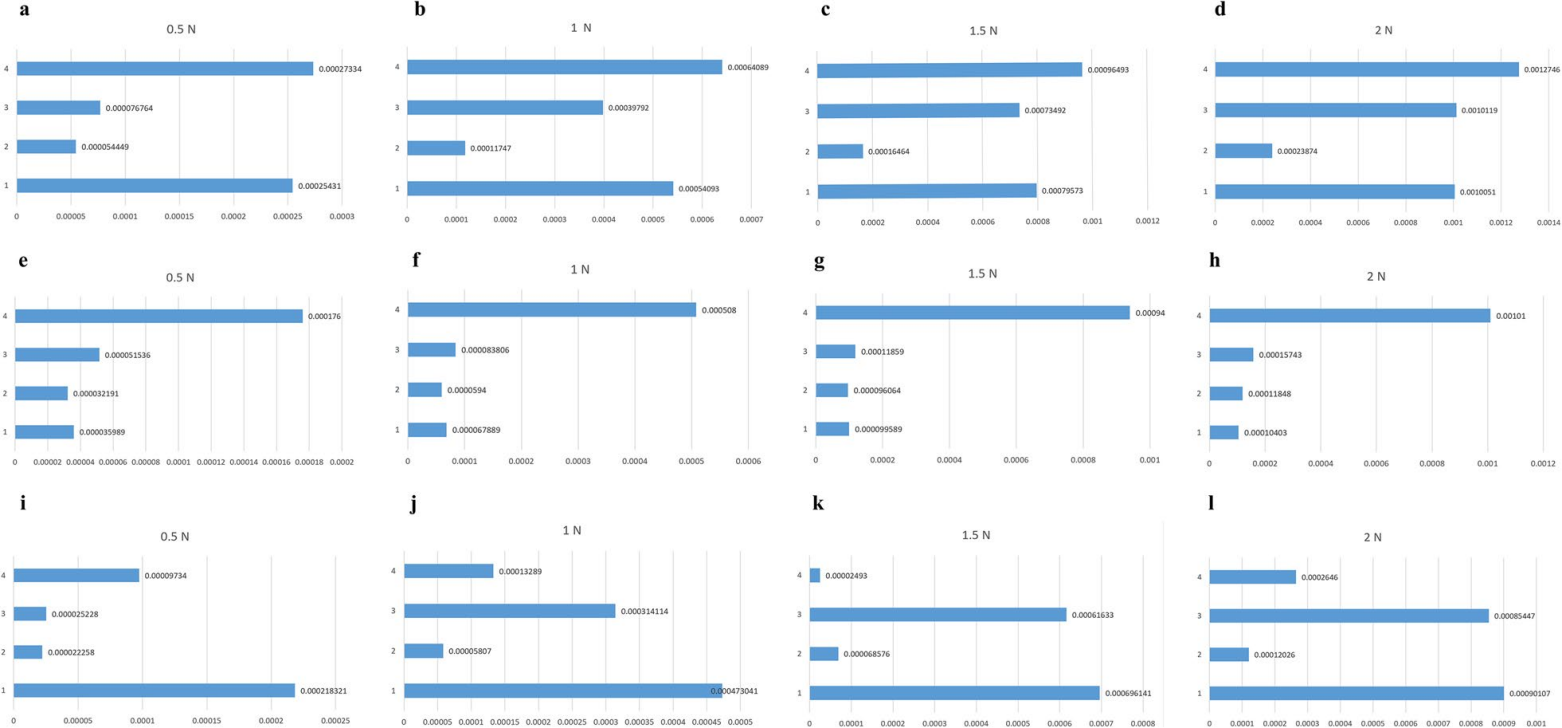
Displacement contrast. (**a**–**d** Incisor end; **e**–**h** Root tip; **i**–**l** Incisor end-Root)

In the 16 groups (Tables 5 and 6), the crown of the maxillary incisors hardly caused buccal movement of the posterior teeth under the action of torque, while the root of the incisors moved toward the palate. The use of micro-implants to control the dentition increased the displacement of the cut end and root end and the difference between the cut end and the root tip. Thus, the incisor end moved slightly anteriorly, and the root tip moved posteriorly. The root control torque movement was realized. The tooth displacement did not change in the up–down and left–right axes, except for torque changes, because the finite element model was static. With the loading of the force value of the four-curvature auxiliary arch, the molar displacement had no movement. Thus, the torque force exerted by the four-curvature auxiliary arch did not affect the movement trend of the maxillary dentition (Figs. 5 and 6).

**Table 5:** Incisor displacement and periodontal stress

Group	Displacement (incisor) (mm)			Stress value of periodontal membrane (incisor) (N/mm^2^)	
	Incisor end	Root tip	Incisor end-root tip	Max	Min
1^a^	0.000254310	− 0.000035989	0.000218321	0.0047893	− 0.0032621
1^b^	0.000540930	− 0.000067889	0.000473041	0.0078286	− 0.0197540
1^c^	0.000795730	− 0.000099589	0.000696141	0.0114920	− 0.0410020
1^d^	0.001005100	− 0.00010403	0.000901070	0.0157670	− 0.0538470
2^a^	0.000054449	− 0.000032191	0.000022258	0.00099313	− 0.0031226
2^b^	0.000117470	− 0.00005940	0.000058070	0.00085706	− 0.0074521
2^c^	0.000164640	− 0.000096064	0.000068576	0.0039847	− 0.0156170
2^d^	0.000238740	− 0.00011848	0.000120260	0.0069679	− 0.0572270
3^a^	0.000076764	− 0.000051536	0.000025228	0.0028015	− 0.0071706
3^b^	0.000397920	− 0.000083806	0.000314114	0.0068559	− 0.0152690
3^c^	0.000734920	− 0.00011859	0.000616330	0.0142670	− 0.0401570
3^d^	0.001011900	− 0.00015743	0.000854470	0.0190740	− 0.0773010
4^a^	0.000273340	− 0.00017600	0.000097340	0.0035990	− 0.0097367
4^b^	0.000640890	− 0.00050800	0.000132890	0.0105710	− 0.0161160
4^c^	0.000964930	− 0.00094000	0.000024930	0.0178110	− 0.0482940
4^d^	0.001274600	− 0.00101000	0.000264600	0.0226450	− 0.0972490

The a, b, c, d respectively correspond to the (0.5, 1, 1.5, 2) N. Where ^a^ stands for 0.5 N, where ^b^ stands for 1 N, where ^c^ stands for 1.5 N, where ^d^ stands for 2 N

**Table 6:** Stress value of periodontal membrane of incisor and molar

Grouping	Stress value of periodontal membrane(incisor)		Stress value of periodontal membrane(molar)	
	Max	Min	Max	Min
2^a^	0.00099313	− 0.0031226	0.00337020	− 0.02439000
2^b^	0.00085706	− 0.0074521	0.00337020	− 0.02439000
2^c^	0.00398470	− 0.0156170	0.00337020	− 0.02439000
2^d^	0.00696790	− 0.0572270	0.00337020	− 0.02439000
4^a^	0.00359900	− 0.0097367	0.00030103	− 0.00047396
4^b^	0.01057100	− 0.0161160	0.00030103	− 0.00047396
4^c^	0.01781100	− 0.0482940	0.00030103	− 0.00047396
4^d^	0.02264500	− 0.0972490	0.00030103	− 0.00047396

The a, b, c, d respectively correspond to the (0.5, 1, 1.5, 2) N. Where ^a^ stands for 0.5 N, where ^b^ stands for 1 N, where ^c^ stands for 1.5 N, where ^d^ stands for 2 N

For the molars (Table 6), some pressure stresses were observed on the root surfaces, but all molar periodontal membrane stress values of the first molar did not exceed 2.6 × 10^−2^ N/mm^2^. In all group models, the stress of the periodontal membrane of the root tip did not exceed 2.6 × 10^−2^ N/mm^2^ under the action of the four-curvature auxiliary arch pedicle force. Thus, the absorption of the root tip was not induced (Figs. 5 and 6).

### Periodontal membrane von mises stresses of the maxillary dentition

**Fig. 7:**
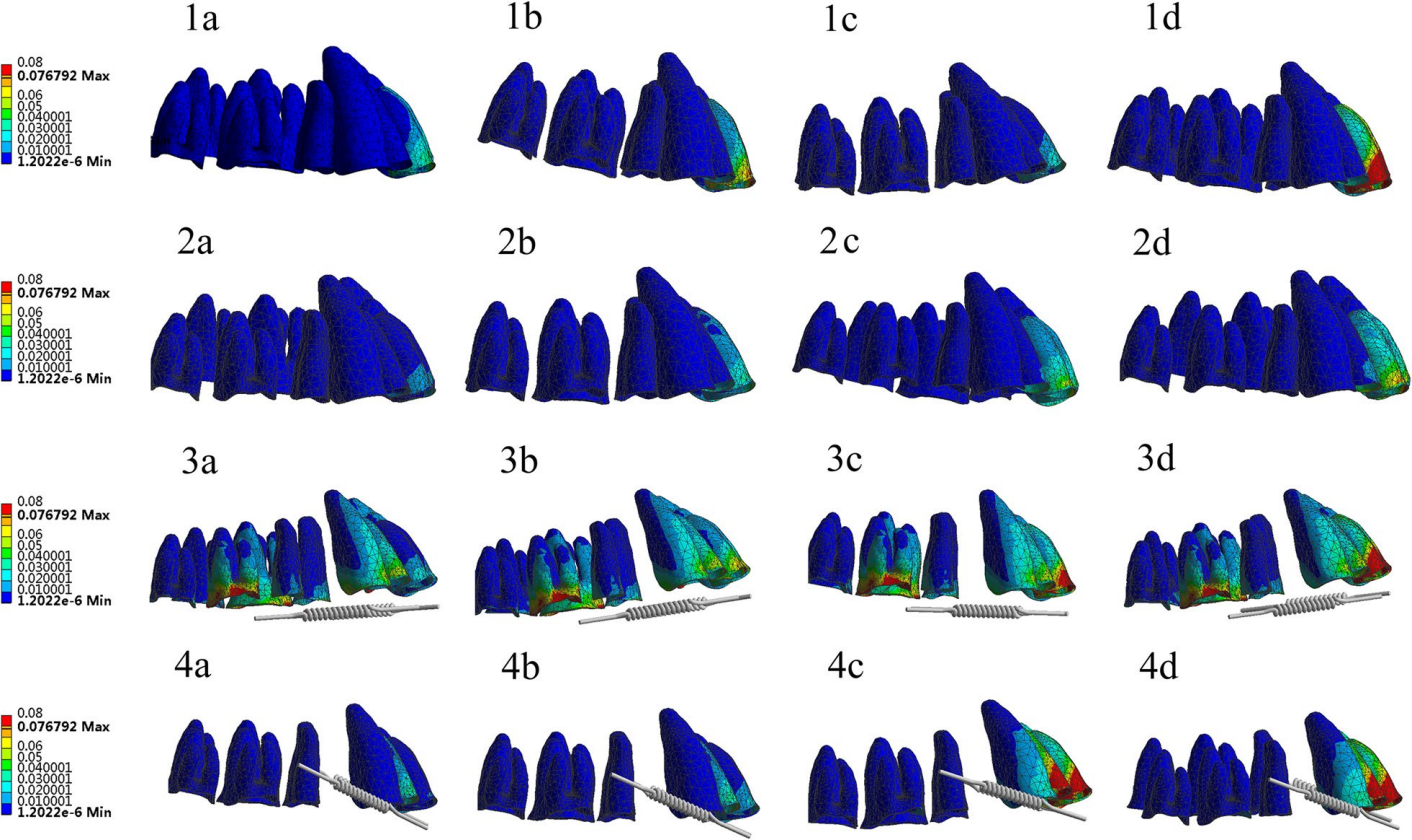
Periodontal membrane

For the incisor (Tables 5 and 6), the stress value in the apical part was considerably less than 2.6 × 10^−2^ N/mm^2^ [21], in all groups. The maximum stress value of the periodontal membrane occurred in the cervical region of the buccal side, and with an increase in the torque force value, the stress value of the periodontal membrane increased. Comparison of 16 kinds of models showed that the periodontal membrane stress values in the cervical incisor buccal side in groups 1a, 1b, 2a, 2b, 2c, 3a, 3b, 4a, and 4b were below 2.6 × 10^−2^ N/mm^2^ (Fig. 7). It can be seen from the figure that the root stress on the distal (negative) to the mesial (positive) of incisors does not significantly change, Because of torque motion, the buccolingual (or buccopalatal) axis is changed.

Among the maxillary teeth, the incisors are the most prone to root resorption, followed by the molars in the maxillary arch [22]. Studies stated that root resorption in premolars and molars may be trivial. In the present experiment, root absorption did not occur in all the maxillary dentition group models [23, 24]. However, in Table 5, the periodontal membrane stress of the root tip increased with increasing torque force of the anterior teeth and did not exceed the maximum stress value in the end; as such, it was considered safe for the root tip.

### PDL stress distribution

**Fig. 8:**
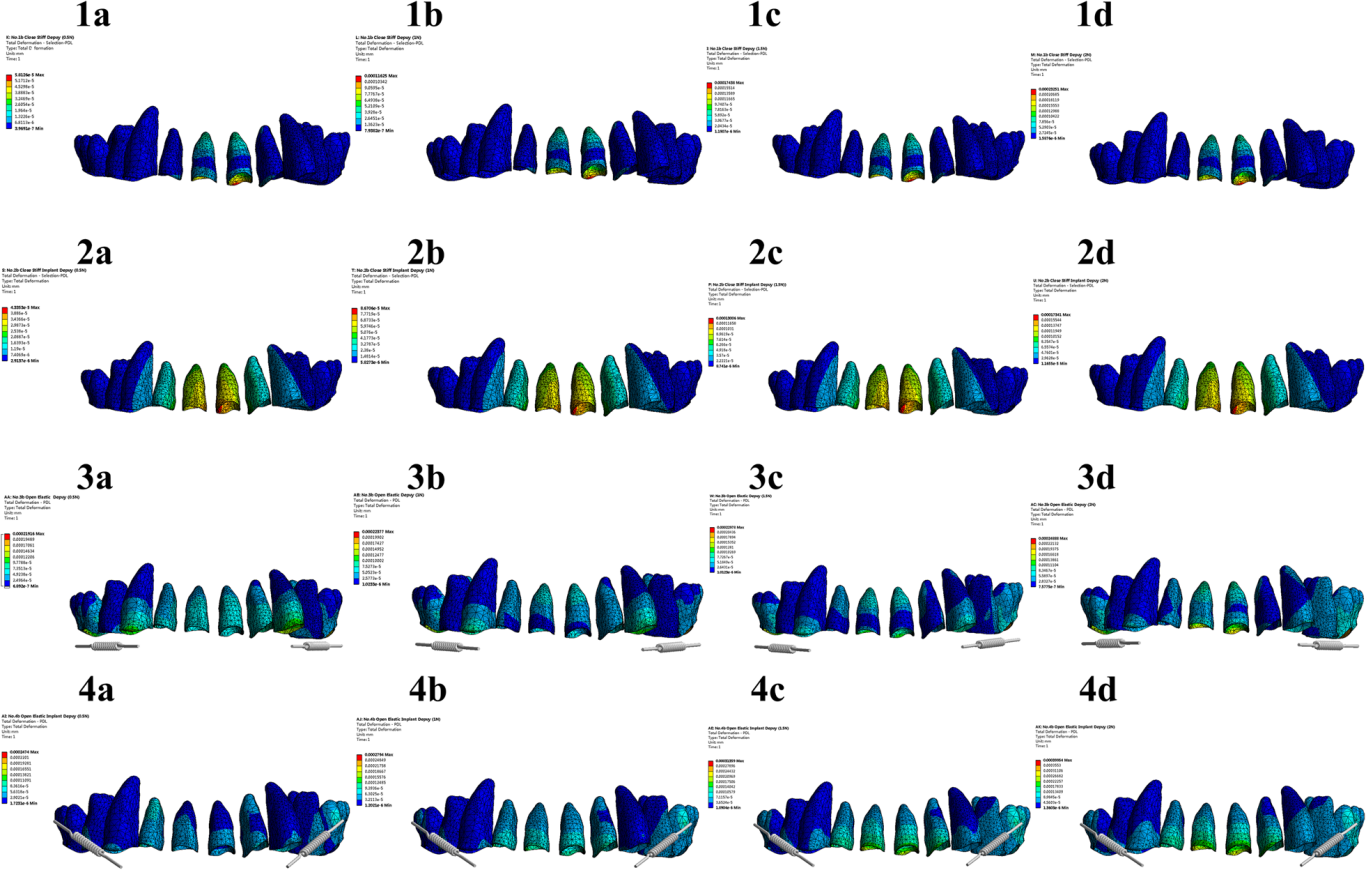
Hydrostatic stress PDLs

Based on the finite element simulation, all three inherent values of the stress tensors (σ 1, σ 2, und σ 3) and hydrostatic stress σ H = 1/3 (σ 1 + σ 2 + σ 3) were calculated in the root area, periodontal ligament, and alveolus of the incisors [25, 26]. The red regions denote the areas under compression with a stress value. Dangerous stress areas started to emerge on the PDL cervical part when the force was increased to 2 N; at the same time, most of the PDL root areas were low-stress areas; hence, resorption was more likely to occur in the cervical region of the incisors. The strain nephograms of the PDL with different movements are shown in Fig. 8. The red region denotes that the strain of the PDL was tensile (positive strain). The blue region indicates that the strain was compressive (negative strain). Increases in hydrostatic pressure and high-pressure areas (higher than 0.0047 N/mm^2^) corresponded with more severe root resorption. Values exceeding them belong to dangerous stress areas with a possibility of root resorption. The regions indicated by other colors represent low-stress areas, where the tooth would not move or move at only a low rate.

The percentages of different PDL areas, including good stress areas, dangerous stress areas, and good strain areas, resulting from changes in orthodontic force under various movements were calculated. After calculation of PDL, all of the distal (negative) to the mesial (positive) part of the incisors did not exceed the value of 0.0047 N/mm^2^. The 1c, 2d, 3c, and 4c buccolingual (or buccopalatal) axis of the incisors exceeded the value of 0.0047 N/mm^2^.

In summary, the torque force applied to the molar ligation, molar retraction, and micro-implant retraction groups should not exceed 1 N. The torque force to be applied to the micro-implant ligation group should not exceed 1.5 N. These values indicated the safe use of the four-curvature auxiliary arch (Additional file 2).

### Clinical findings

**Fig. 9:**
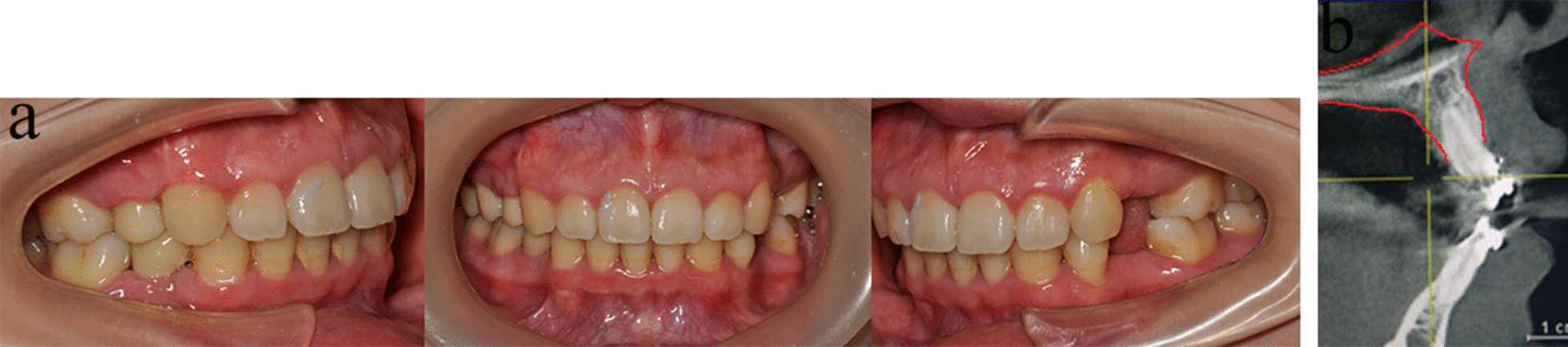
Intraoral photographs & CBCT Treatment of complete

**Fig. 10:**
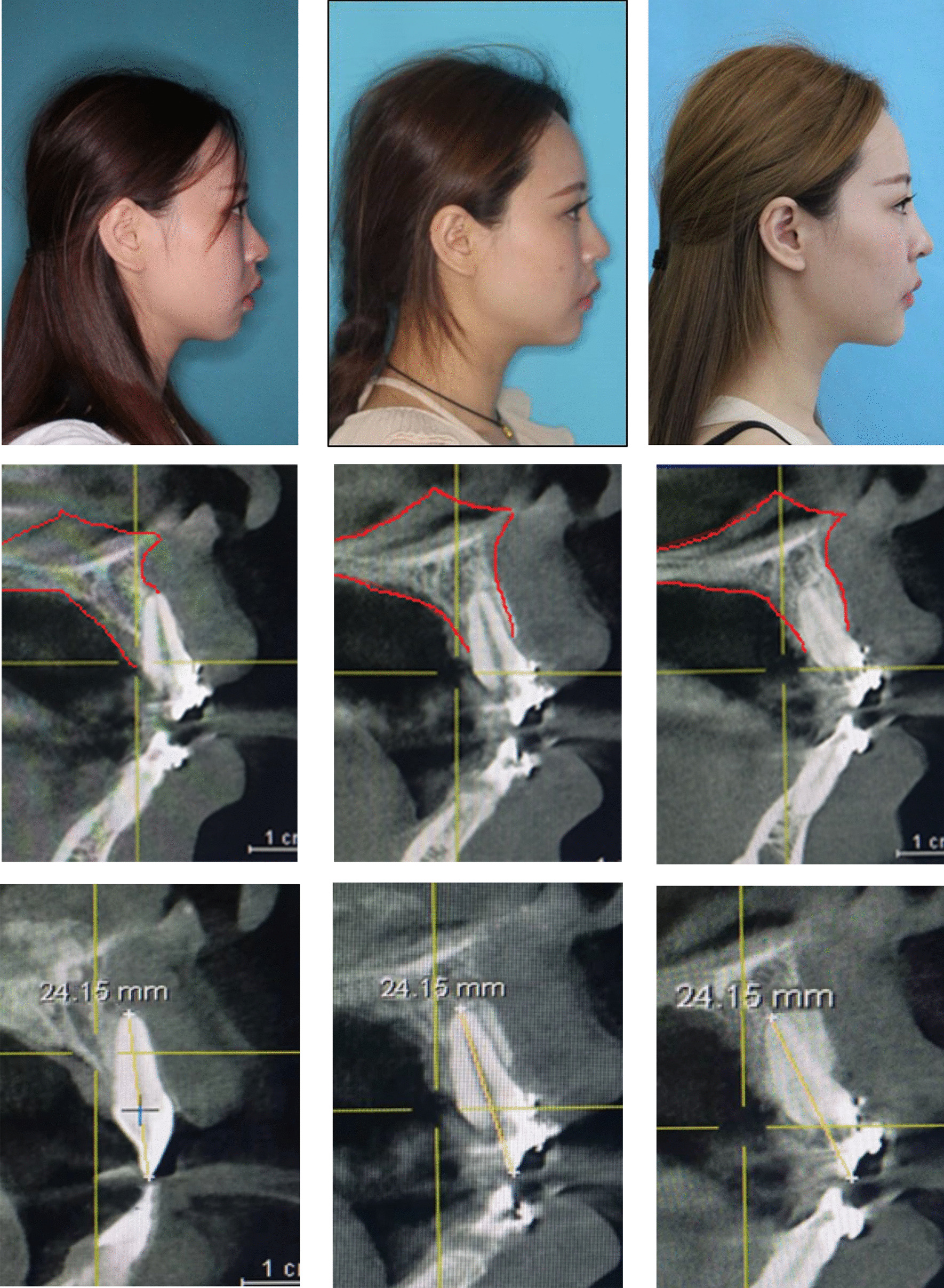
CBCT show tooth root torque change & Root length & Lateral photographs

After the treatment of the four-curvature auxiliary arch, the root of the anterior teeth completely entered the maxillary alveolar bone, and the bone fenestrations disappeared. In addition, the crown of the incisors did not have excessive buccal lateral tilt, and the whole upper and lower dentition had good occlusion. No root resorption occurred during the whole treatment. Changes in the soft and hard tissues before and after treatment were evaluated, and the treatment resulted in an ideal chin shape and good facial balance (Figs. 9 and 10) (Additional file 1).

## Discussion

Different methods have been used to control the root inclination of maxillary incisors. Al Ihmam revealed that torque control after corticotomy was good. However, in the study, torque control was not sufficient when limiting corticotomy to the vestibular side only. The use of TISADs during retraction also significantly reduced the inclination of the maxillary incisor. This result was probably due to the fact that the vector of force used for retraction approached the center of resistance of the teeth more closely than during standard retraction. However, the conclusion was simple, and evidence to support this inference was insufficient [27]. Statistical analysis showed that the use of additional components that control the root inclination resulted in a small loss of torque during retraction compared with the closing gaps after missing teeth without the use of these methods. The common additional components used in torque auxiliary arches in the clinic were the anterior root torquing auxiliary arch (ART) and gate spring. ART was not only complex by the ligation method, it would cause dental arch expansion such that the dental arch undergoes adverse changes [28, 29]. The gate spring was an analog of the Warren spring. Meanwhile, the U-loop could only improve the torque of a single tooth [30, 31]. The four-curvature auxiliary arch had a simple installation process, with four finger spikes as the force-applying part and the circular ring structure at the end as the force-applying point. This configuration made the force more concentrated and increased the efficiency of torque realization. At the same time, the use of microimplants could better stabilize the main arch wire and improve bone fenestrations and root surface exposure caused by improper torque of the incisors.

In this experiment, 0.022 inch (0.5588 × 0.7112 mm) bracket was used to place 0.019*0.025 (0.483 × 0.635 mm) SS wire in the groove, so 0.001 inch had a clearance angle of 3.77°. In total, 0.003 inches created a clearance angle of approximately 12°. The resulting gap angle changed the torque angle of the incisor during the stress process (incisal and occlusal), and the surface could not be kept stationary, resulting in incisal and occlusal surface displacement in the opposite direction [27, 32]. Although the incisal and occlusal surfaces were displaced due to uncontrollable factors in the experiment, the data showed that the displacement of the root was remarkably larger than that of the incisor and occlusal surface displacement. Thus, the root of the incisors entered the maxillary. The results showed that the long axis of the incisors was not tilted and considerably moved, and the root control effect was obvious [10, 32].

A previous study has reported that a force of 2.6 × 10^−2^ MPa N/mm^2^ was the maximum stress value that may be applied to the periodontal membrane, and this limit was safe and effective in orthodontic treatment [33]. Any other amount of force may result in periodontal ischemia, leading to irreversible necrosis of the surrounding tissue. In the present study, the tooth stress response was represented by different colors [34] [35]. The stress value of the periodontal membrane was represented by a blue box when the value was < 2.6 × 10^–2^ N/mm^2^, and a red box with gradually deepened color. This color coding supported an intuitive interpretation of the stress response distribution. The tooth displacements are shown in different colors. The colors gradually changed from blue to different colors. This color variation could intuitively explain the distribution of the experimental results [36, 37].

When the torque force was applied to the maxillary incisors, the stress to the periodontal membrane of each part was concentrated at the cervical side of the labial side [38]. In the molar ligation group and the molar retraction group, the buccal tube and microimplant were set to contact the hook. In the micro-implant ligation group and the micro-implant retraction group, the tooth extraction space adduction traction value was 1.15 N. Given that the only spring element in FEA in ANSYS software was set as an expression, the models of adduction tooth extraction space traction of the molar retraction group and the micro-implant retraction group were displayed as a tension spring. Overall, this design was close to the clinical reality and attempted to simulate the clinical therapeutic effect. All the models were simulated according to common clinical treatment states. The micro-implant ligation group could better stabilize the anchorage and exert greater torque force.

The present study involved 16 conditions, and the results showed that in the other 3 groups, the four-curvature auxiliary arch torque force may not exceed 1 N when the auxiliary arch was used. When the arch was used with absolute anchorage, given no tooth extraction space, the recommended force may not exceed 1.5 N [31]. In clinical application, the safety of the auxiliary arch could be judged according to this result. In all groups, torque force values could not exceed 2 N. As the application of torque forces increased, the periodontal membrane stress at the other maxillary teeth, except the incisors, did not exceed the maximum periodontal membrane stress of 2.6 × 10^−2^ N/mm^2^. Therefore, the effect of the four-curvature auxiliary arch was obvious, and the root torque control movement was carried out under the condition of stabilizing the main bow wire at different states. At the same time, the application of microimplants could better assist the auxiliary arch in exerting torque. This study can provide a better basis for clinical application.

The case patient had anterior tooth tilting laterally to the tongue, missing eight premolars, and incomplete closure of the tooth extraction space. Initially, the root of the incisors was located outside the labial cortex of the maxillary alveolar bone. After the use of a four-curvature auxiliary arch, the root was adjusted to the center of the maxillary alveolar bone. No root resorption occurred during the whole treatment. Changes in the soft and hard tissues before and after treatment were evaluated, finally obtaining an ideal chin shape and good facial balance. Microimplants provided independent absolute anchorage during treatment. This report showed that ideal root control results could be achieved in orthodontic treatment by using microimplants in combination with the four-curvature auxiliary arch device.

The highest stresses were observed on the root, followed by the alveolar bone, and finally in the PDL; this finding could due to their differences in mechanical properties. The thickness of the PDL is not uniform in all teeth under the natural condition, which is a limitation of the 3D finite element. In this regard, we used PDL with a uniform thickness of 0.2 mm for all teeth. Mc Guiness et al. used a 3D finite element model of a human maxillary canine and examined a series of forces applied to the teeth. The highest stress concentration in the PDL was found at the cervical margin. Therefore, the stress was concentrated at the cervical margin [16, 17, 19].

The slot dimensions, bracket bonding heights, play between the slot and wire, method of ligation, wire corner radii, and slot deformation on loading were considered to affect the expression of strength during orthodontic treatment [15, 39]. Our further research will refine these details. Although the four-curvature auxiliary arch in this study produced desirable results, more attention should be paid to its aesthetics and self-cleaning problems in future research. In addition, dynamic finite elements could more accurately and comprehensively reflect the effect of tooth movement, which will become our focus in the future. Finally, 3D printing was used to customize personalized auxiliary arch to further improve the clinical application [28, 40] (Additional files 1 and 2).

## Conclusion

The following conclusions could be drawn:

1. For the incisor, the maximum stress of the periodontal membrane occurred at the cervical side of the buccal side. All groups were root movement controllers such that the root of the tooth moved without buccal inclination movement at the cut end of the crown, and more effective torque movement was achieved. The use of the four-curvature auxiliary arch in the incisors did not affect the periodontal health and displacement of the molars and did not change the dental arch morphology.
2. During the application of the auxiliary arch, ligation was the best strategy to stabilize the shape of the entire dental arch. The torque movement of the root could be better realized by using a microimplant and the four-curvature auxiliary arch.
3. In this experiment, the more unit nodes were divided, the closer the property and parameter changes of the material were to the real situation. This modeling method could better simulate the complex oral environment and make the result closer to the real oral environment system.
4. In the experiment, the most easily absorbed position in the application of four-curvature auxiliary arch is the buccal cervical margin.

## Supplementary Information

**Additional file 1.** Clinical application case details.

**Additional file 2.** Finite element raw data.

## Data Availability

All data generated or analysed during this study are included in this published article [and its Additional files].
