# Correction: Efficacy of a four-curvature auxiliary arch at preventing maxillary central incisor linguoclination during orthodontic treatment: a finite element analysis

**DOI:** 10.1186/s12903-023-02947-7

**Published:** 2023-04-18

**Authors:** Ping-Zhu Yang, Li-Yun Bai, He-Xuan Zhang, Wen-Jun Zhao, Yu liu, Xiu-Jie Wen, Rui Liu

**Affiliations:** 1 Department of Stomatology, Daping Hospital, Third Military Medical University (Army Medical University), Chongqing, 400042 China; 2 Department of Orthodontics, School of Dentistry, Southwest Medical University, Luzhou, 646000 China; 3 ChuangNeng Technology (ChongQing) Co. LTD, Chongqing, 400042 China; 4 State Key Laboratory of Trauma, Burns and Combined Injury, Wound Trauma Medical CenterDaping Hospital, Third Military Medical University (Army Medical University), , Chongqing, 400042 China

**Correction: BMC Oral Health 23, 144 (2023)**

**https://doi.org/10.1186/s12903-023-02833-2**

In this article [1] the affiliation details for Rui Liu and Xiu-Jie Wen were interchanged. The correct affiliations are listed in this correction.

The affiliation of author Rui Liu is Department of Stomatology, Daping Hospital, Third Military Medical University (Army Medical University), Chongqing, 400042, China.

The affiliation of author Xiu-Jie Wen is Department of Orthodontics, School of Dentistry, Southwest Medical University, Luzhou 646000, China.

The original article has been corrected.
