# Supplementary material for: Efficacy of a four-curvature auxiliary arch at preventing maxillary central incisor linguoclination during orthodontic treatment: a finite element analysis
Source: BMC Oral Health. 2023 Mar 11;23:144. doi: 10.1186/s12903-023-02833-2 (PMC10007732; doi:10.1186/s12903-023-02833-2)
Supplement: Supplementary file 1 — Additional file 1. Clinical application case details. [file 12903_2023_2833_MOESM1_ESM.docx]

***Application to clinical cases***

All clinical procedures were approved by the Ethical Committee of Army Medical University and performed in accordance with the applicable guidelines. On July 8, 2015, a 24-year-old female was transferred to the stomatology department of the Military Special Medical Center, requiring a correction to the incisor protrusion. The patient presented without any history of systemic disease, infectious disease, or drug allergy. The patient had received orthodontic treatment in a local dental clinic 1 year before, during which eight premolars were removed. After the treatment failed, she was referred to our hospital and asked to be re-treated. The failure of anterior root control resulted in the anterior tongue tilt, likely causing the incisor tongue inclination, precluding the closing of the tooth extraction gap. The patient was transferred to our clinic to correct this outcome. The treatment was organized as follows.

1. Torque control of the incisors required improvement due to the over-exposure of the anterior root to the bone cortex and given the defect in dentition; a non-tooth extraction fixed straight wire arch treatment was proposed.

2. Due to the absence of A45B45C45D25, which is a tooth extraction gap, so each of the four areas in the later stage premolars will be repaired.

3. After aligning the maxillary teeth, mandibular fixation and correction were performed, using a maxillary flat bite plate. C7D7 was included in orthodontic treatment to correct the incorrect occlusion of A7C7, and to guide the mandible forward to improve the relationship between deep overbite and deep cover.

4. The maxillary micro-implant anchorage stabilize the main bow wire. The maxillary four-curvature auxiliary arch corrected the torque of the anterior teeth as well as the position of the incisor root in the middle of the alveolar bone, improving the soft tissue profile of the anterior teeth, including their relationship to the [mandible](javascript:;).(Fig. 1,2)


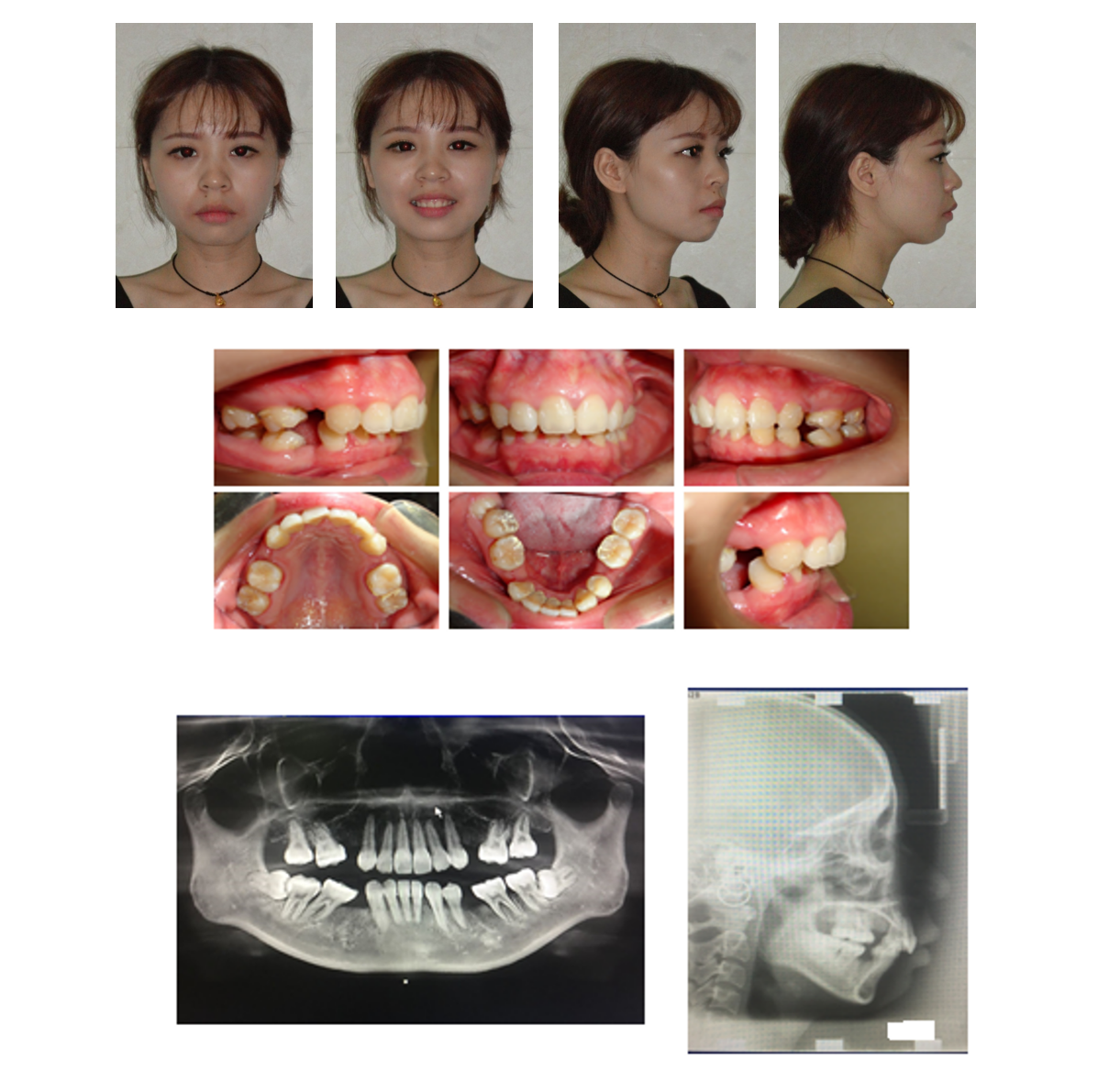


Fig 1 Photographs and radiographs from our hospital after referral


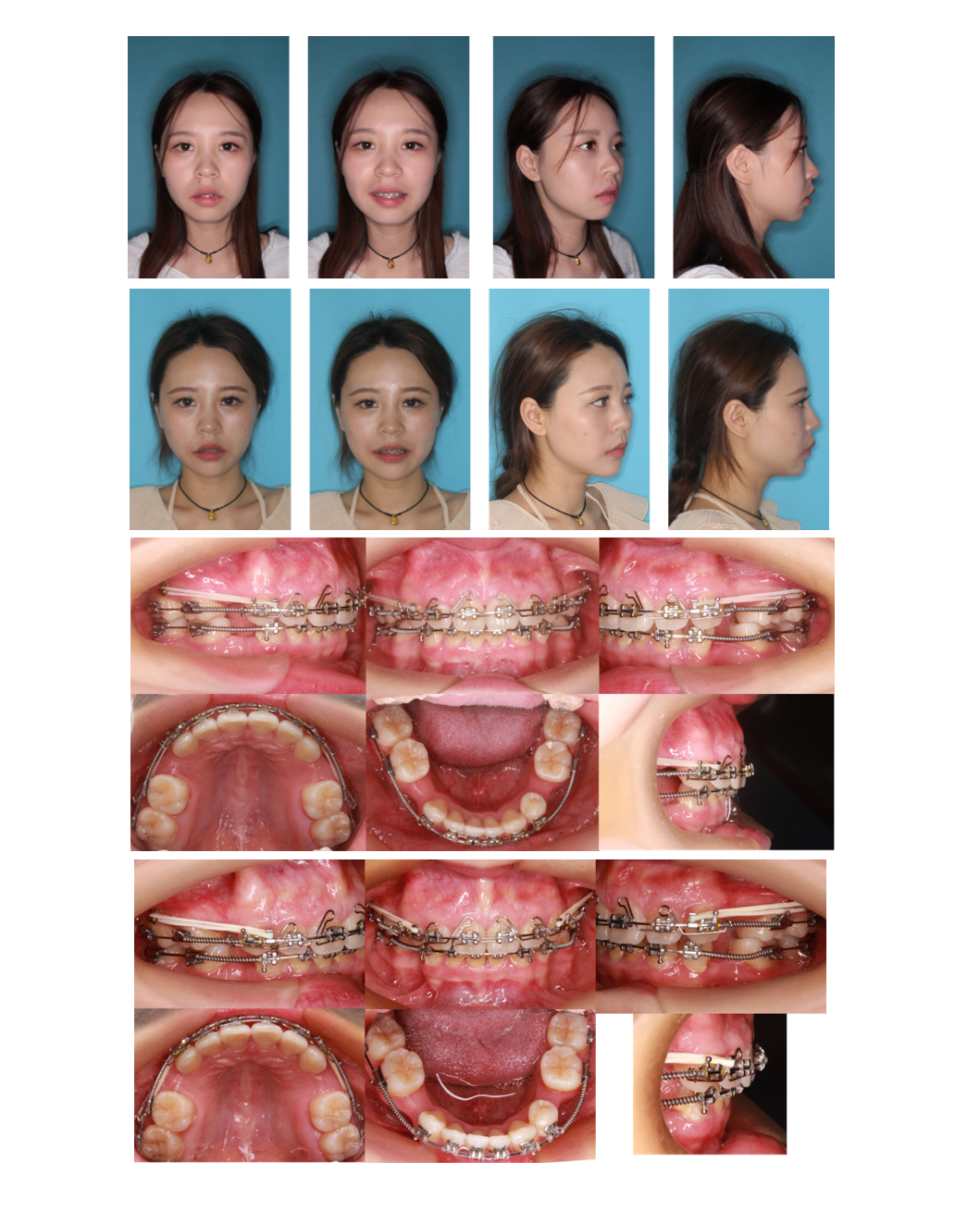


Fig 2 four-curvature auxiliary arch in the use of the process

***Efficacy of the four-curvature auxiliary arch***

The four-curvature auxiliary arch was used to rotate the anterior teeth, and the torque correction effect of the anterior teeth was noticeable. The use of the four-curvature auxiliary arch improved the torque of the front teeth and reduced the risk of root exposure to soft tissue, which effectively improved the soft tissue profile. After the correction of the four-curvature auxiliary arch, the root of the anterior teeth entered the central position of the maxilla from the outside of the bone cortex to avoid the occurrence fenestrations of bone make the cortical bone continuous. The four-curvature auxiliary arch did not cause the absorption of the anterior tooth root, reducing the risk of root resorption; these findings suggest this approach may be safely used in orthodontic treatment to improve the torque of the anterior teeth. (Fig 3)


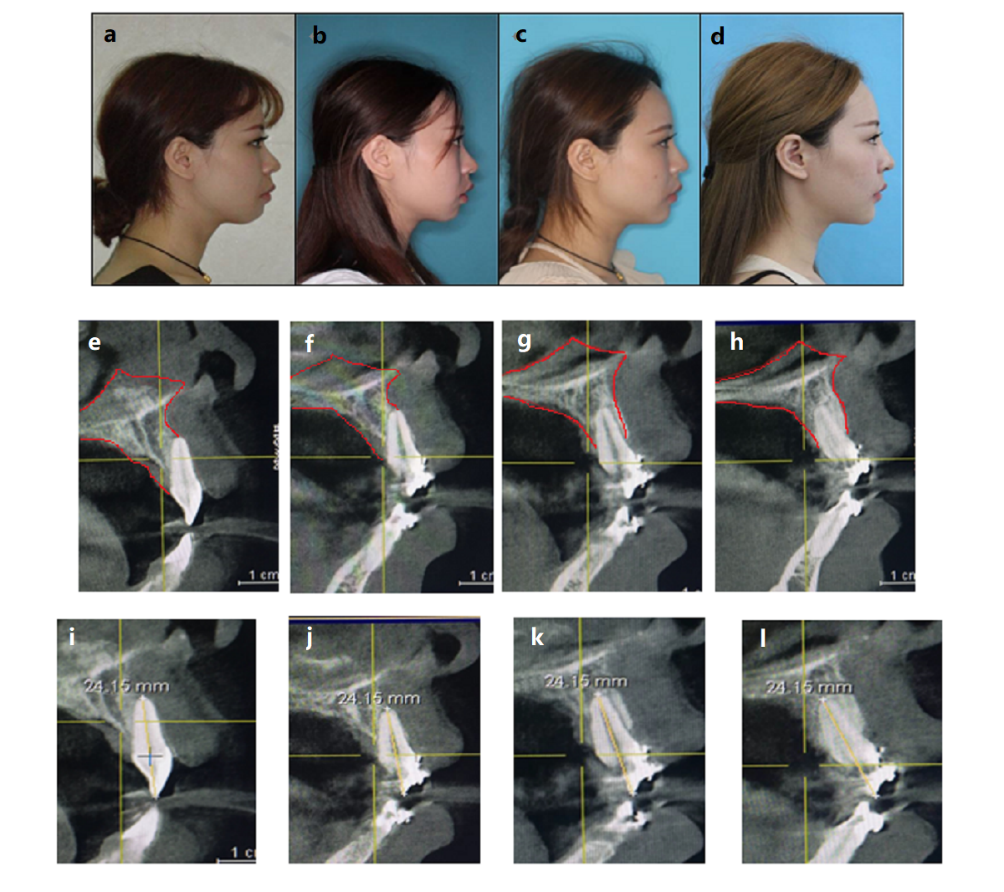


Fig 3 CBCT scans show torque change of the front teeth with no significant root resorption in the anterior teeth

Table 1 Lateral radiograph comparison

The case patient initially presented at the stomatology department with anterior tooth tilting laterally to the tongue, missing eight premolars, and incomplete closure of the tooth extraction gap. During treatment, the torque of the incisors teeth laterally inclining to the tongue was significantly improved after the use of miniature implants and the four-curvature auxiliary arch; the impact of the four-curvature auxiliary arch was easily observed. Initially, the root of the incisor was located outside the labial cortex of the maxillary alveolar bone. However, it was adjusted to the center of the maxillary alveolar bone with the use of the four-curvature auxiliary arch. No root resorption occurred during the whole treatment process. The treatment resulted in an ideal chin shape and good facial balance. Changes to soft and hard tissues before and after treatment were evaluated. The micro-implant provided independent absolute anchorage during treatment, which supported the biomechanics of orthodontics. The present report shows that the use of a micro-implant combined with the four-curvature auxiliary arch device may help achieve desirable outcomes in orthodontic treatment, including the correction of poor or excessive torque loss in the maxillary incisors. (Fig 4,Table 1)


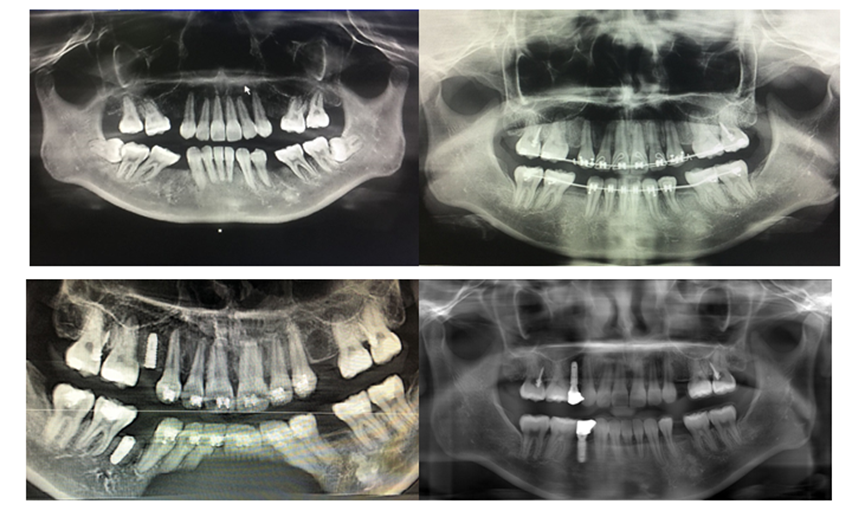


**Fig. 4 Radiographs (Before - In the treatment - After - Return visit)**
