# Supplementary material for: Efficacy of a four-curvature auxiliary arch at preventing maxillary central incisor linguoclination during orthodontic treatment: a finite element analysis
Source: BMC Oral Health. 2023 Mar 11;23:144. doi: 10.1186/s12903-023-02833-2 (PMC10007732; doi:10.1186/s12903-023-02833-2)
Supplement: Supplementary file 2 — Additional file 2. Finite element raw data. [file 12903_2023_2833_MOESM2_ESM.docx]

**All data generated or analysed during this study are included in this published article [and its supplementary information files.**

**Supplementary Fig S1-S16** **is the data generated by tooth displacement after 3d finite element modeling,** **The data in the figure show the displacement, von Mises stress, and hydrostatic stress PDLs of all the components. changes of different parts of teeth under the action of the self-made four-curvature.**


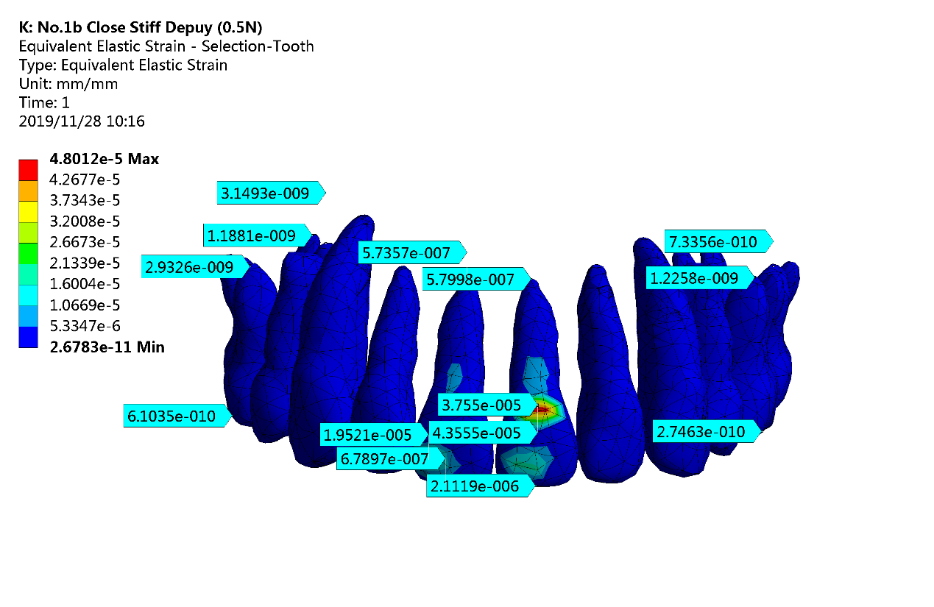


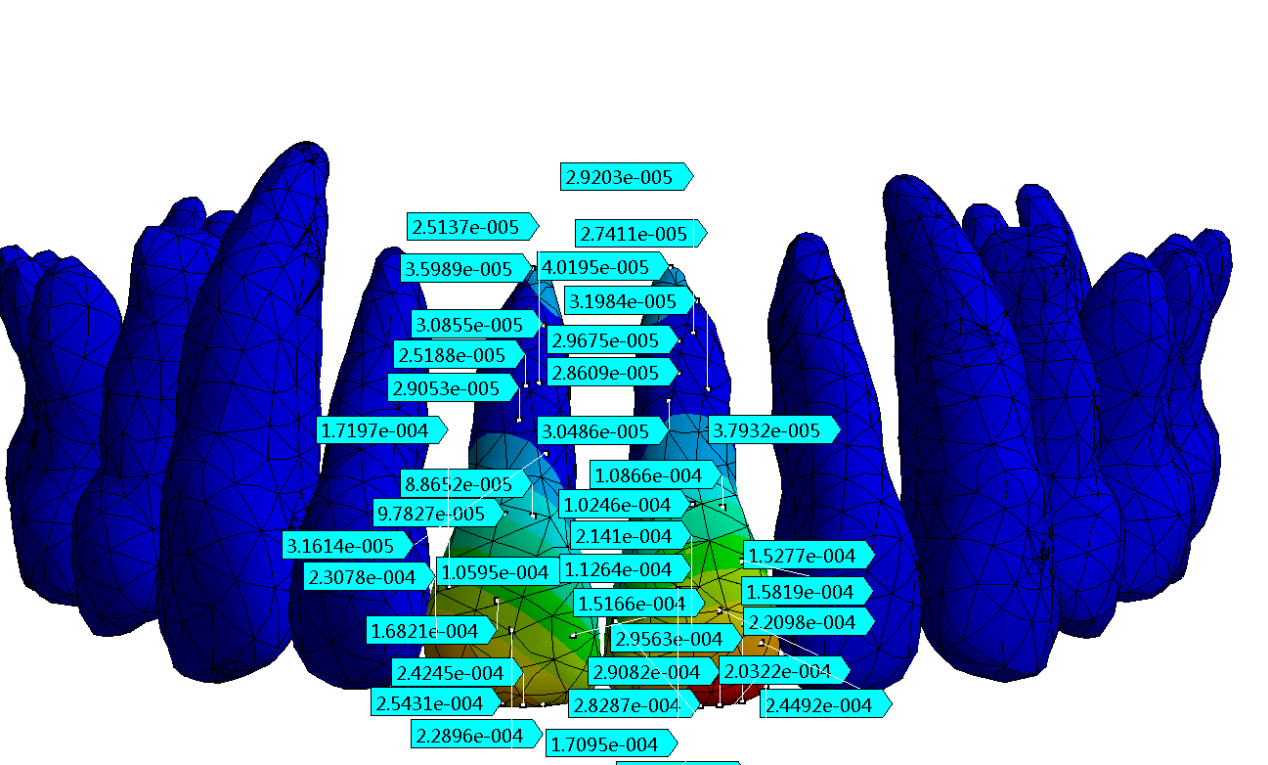

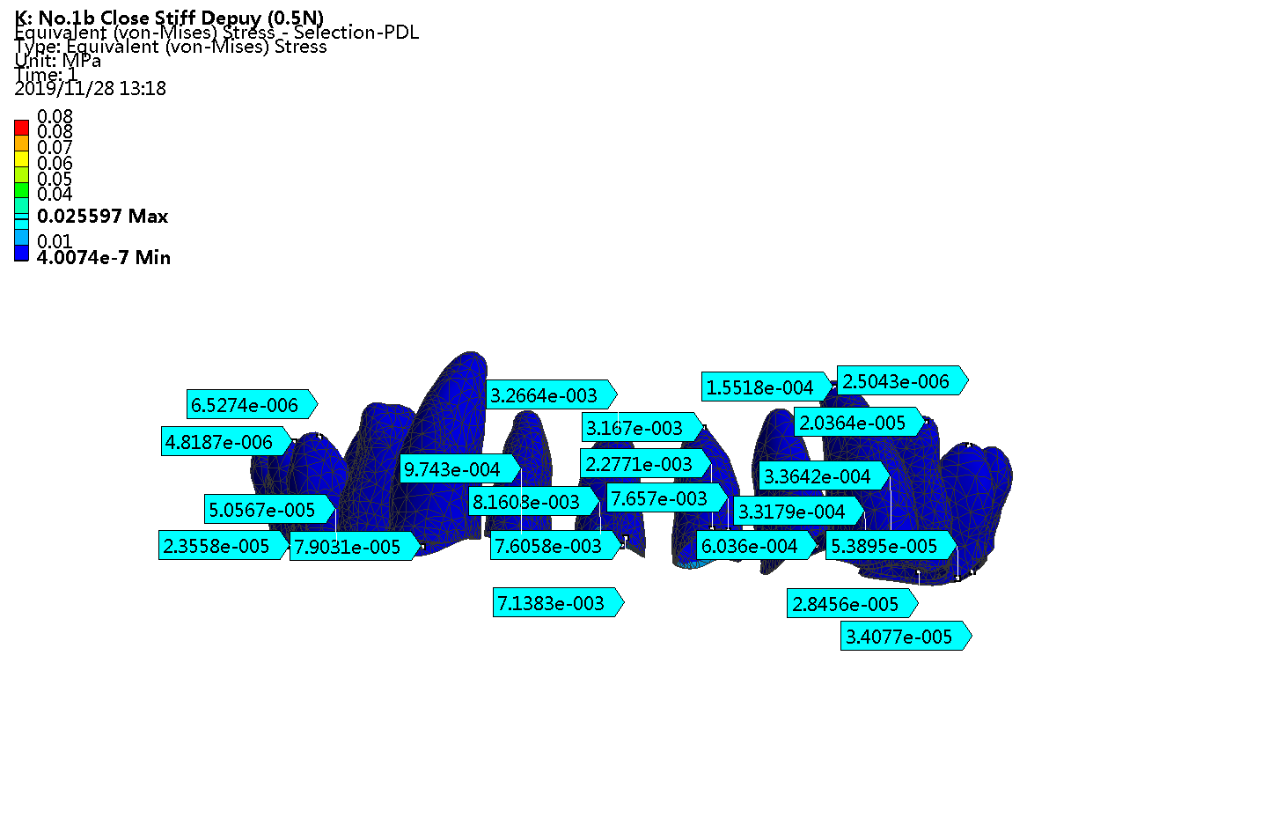

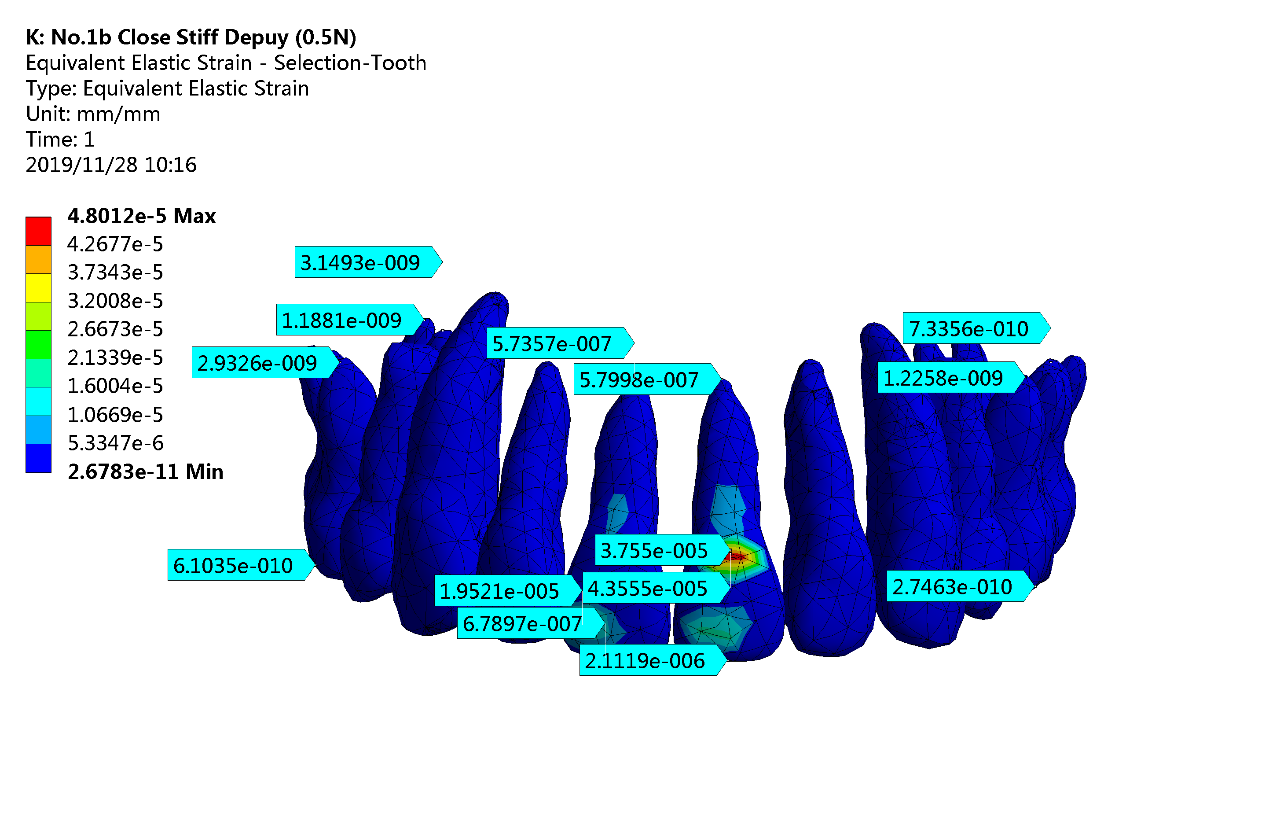


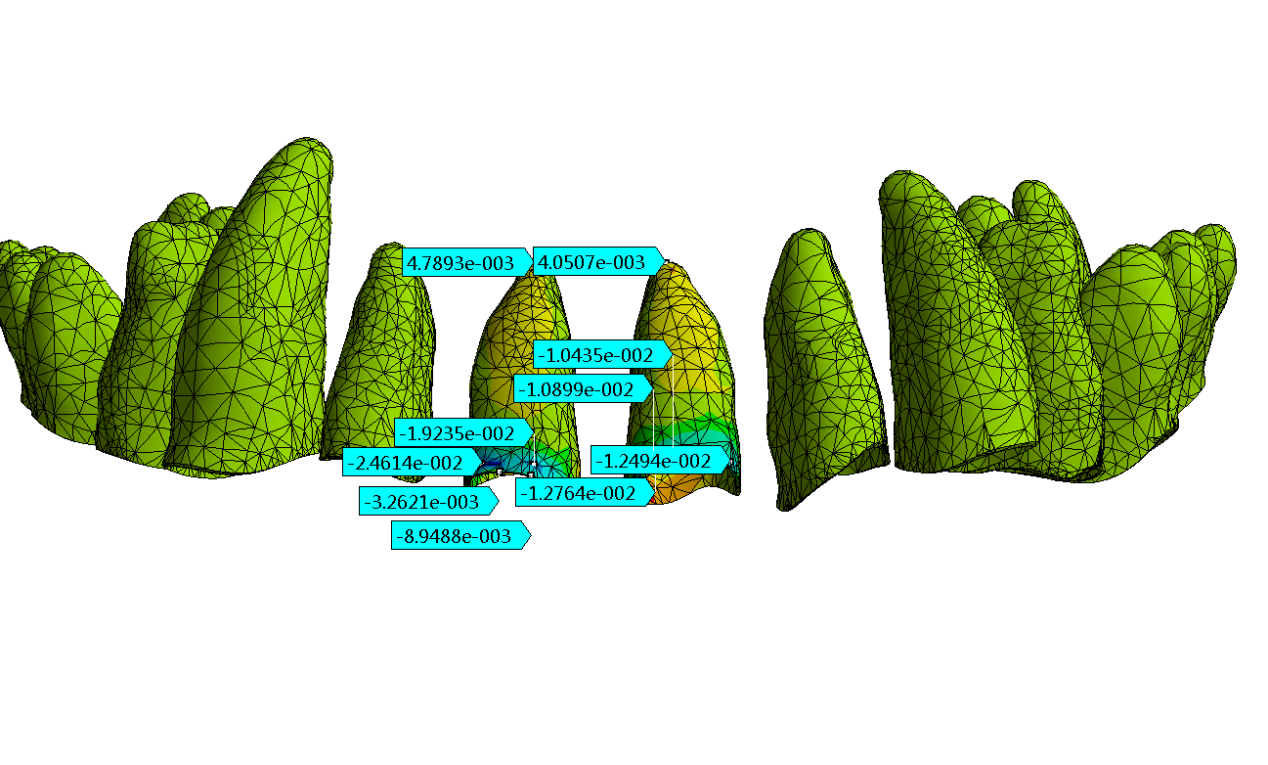


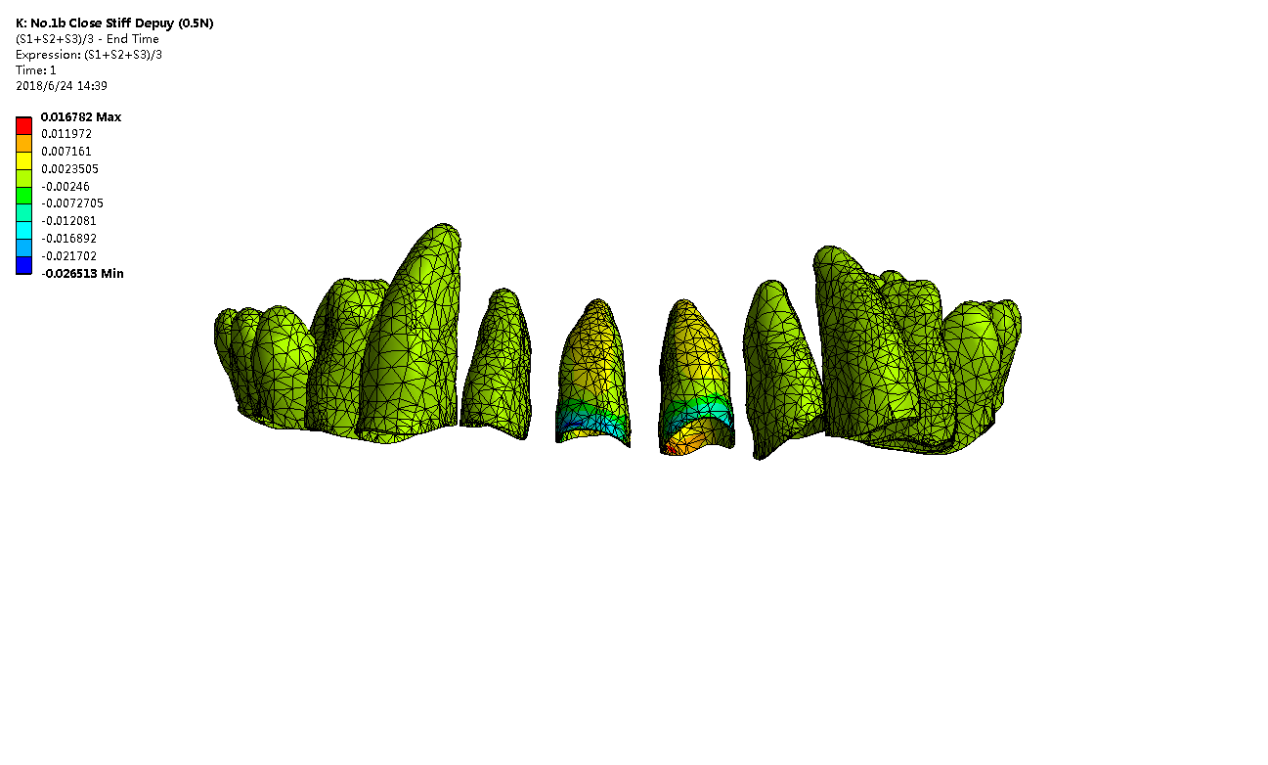

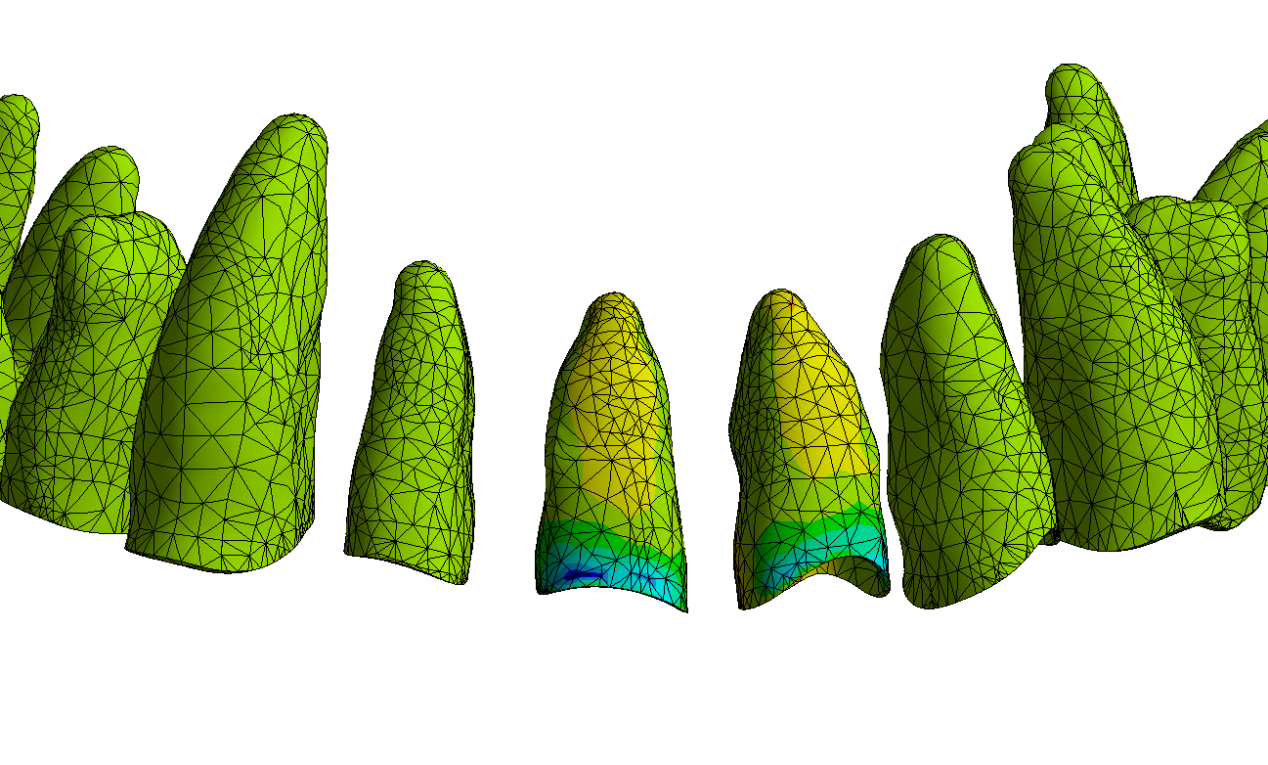

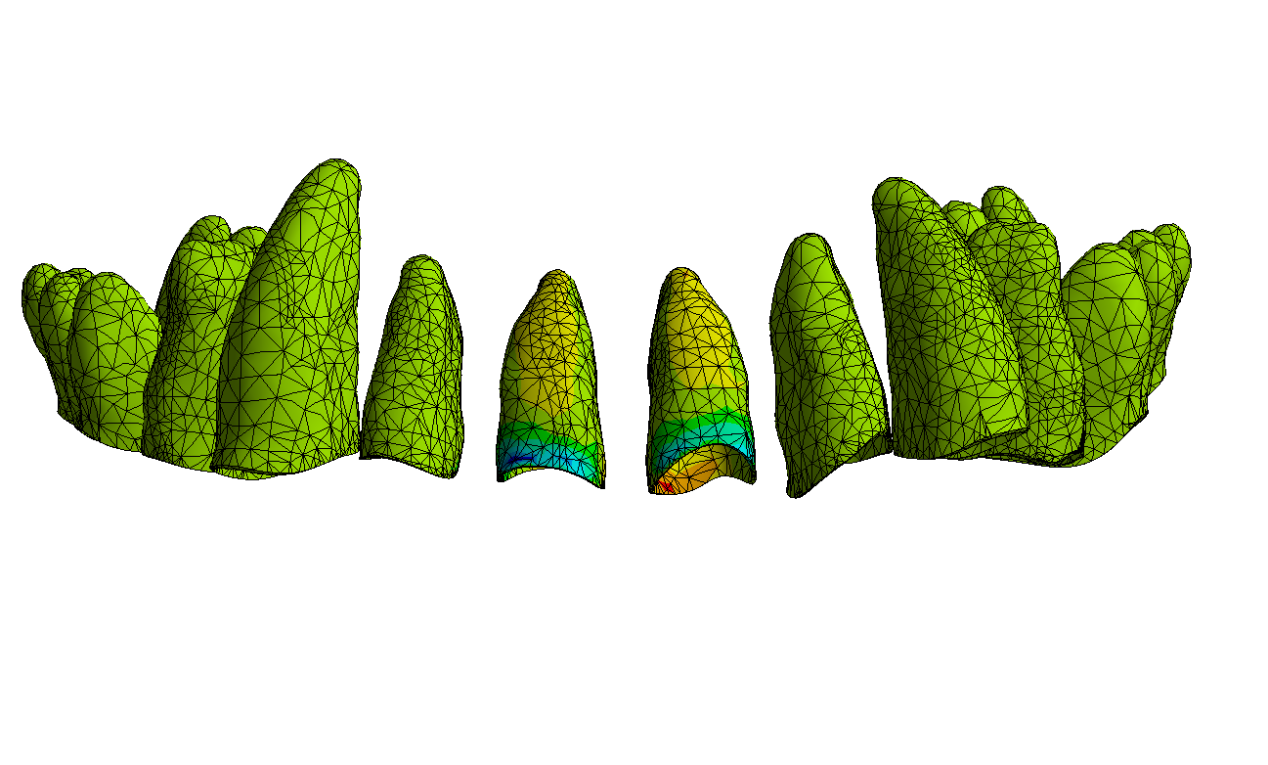


**Supplementary Fig S1: 1a group**


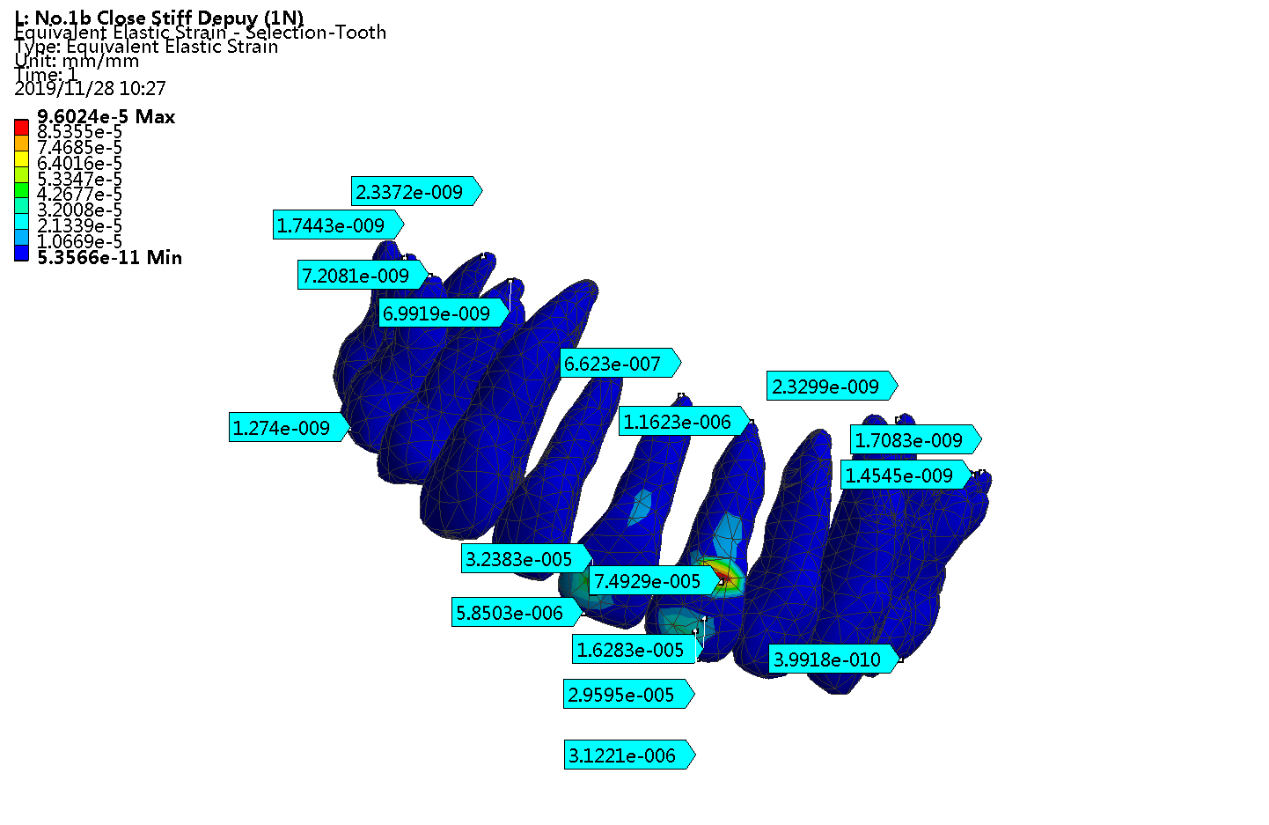

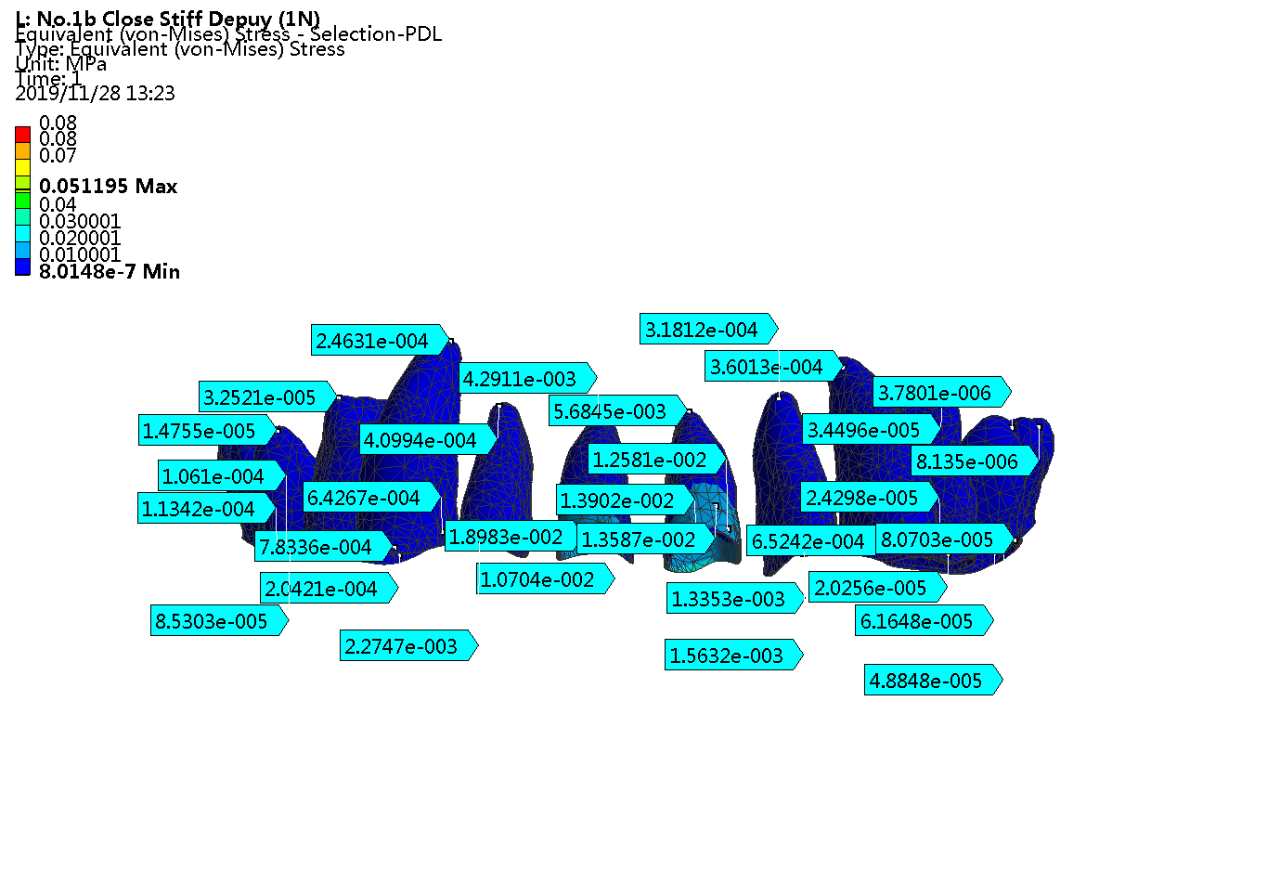

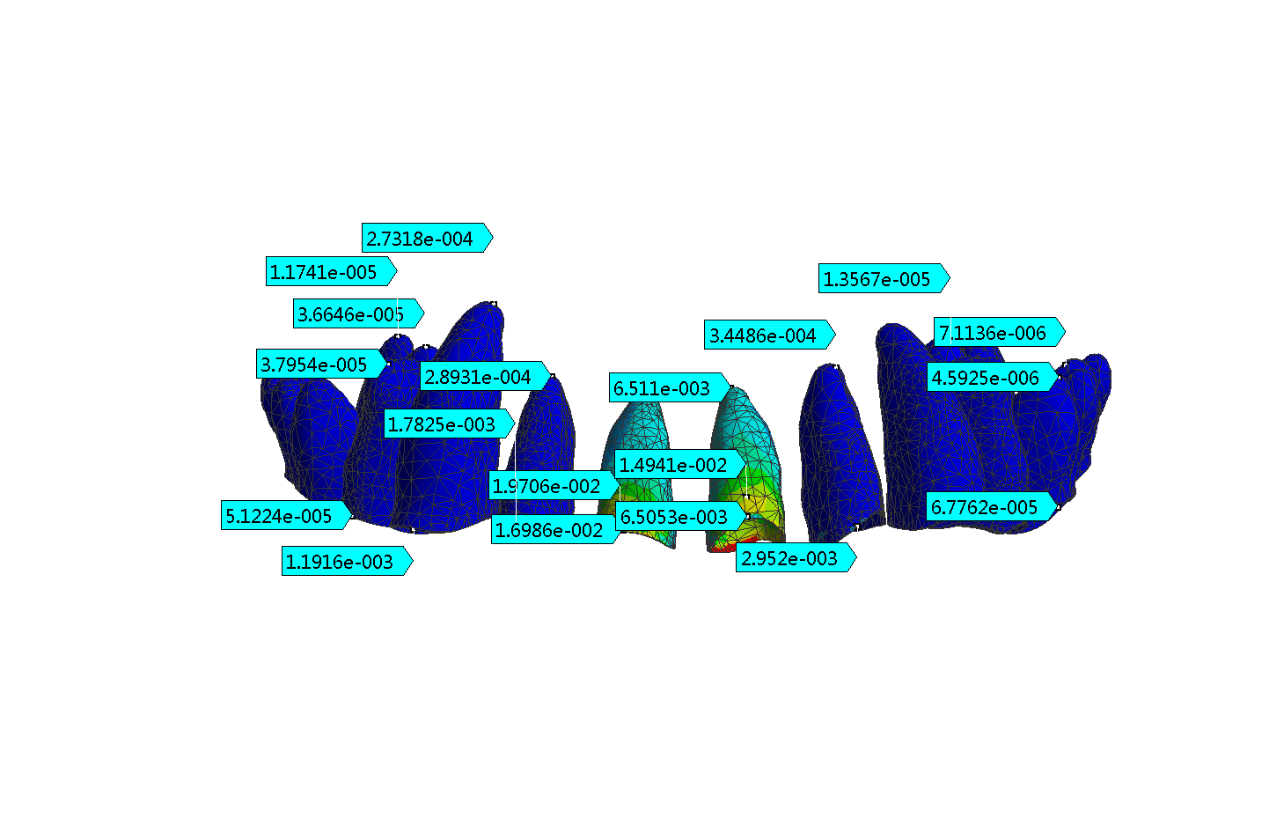

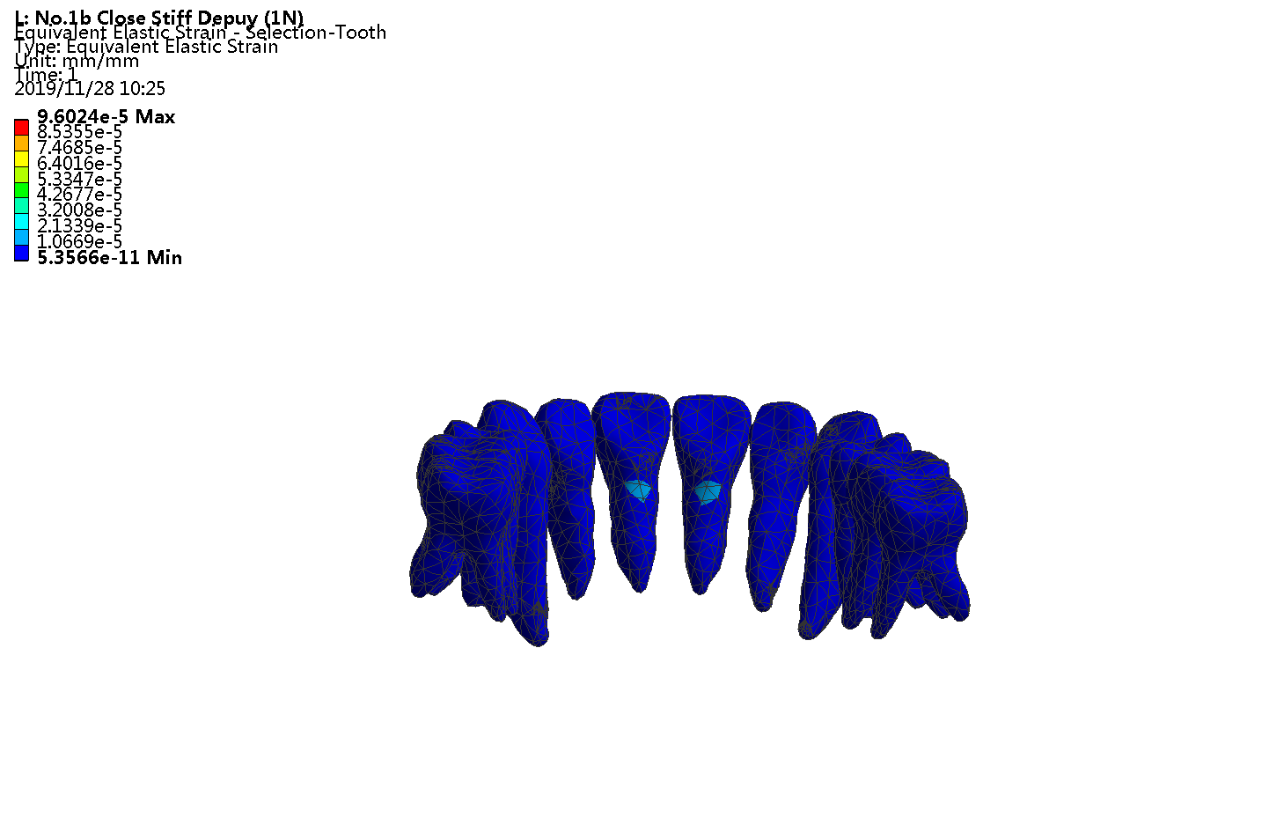


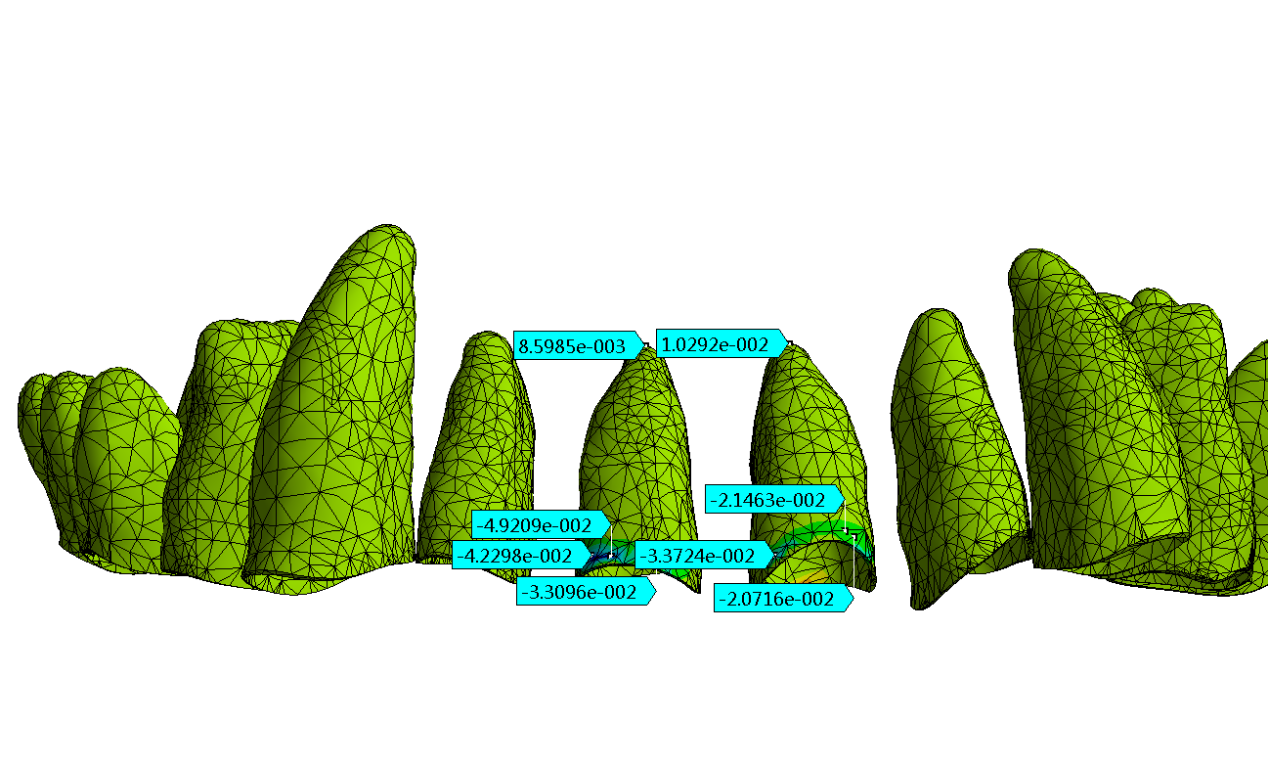

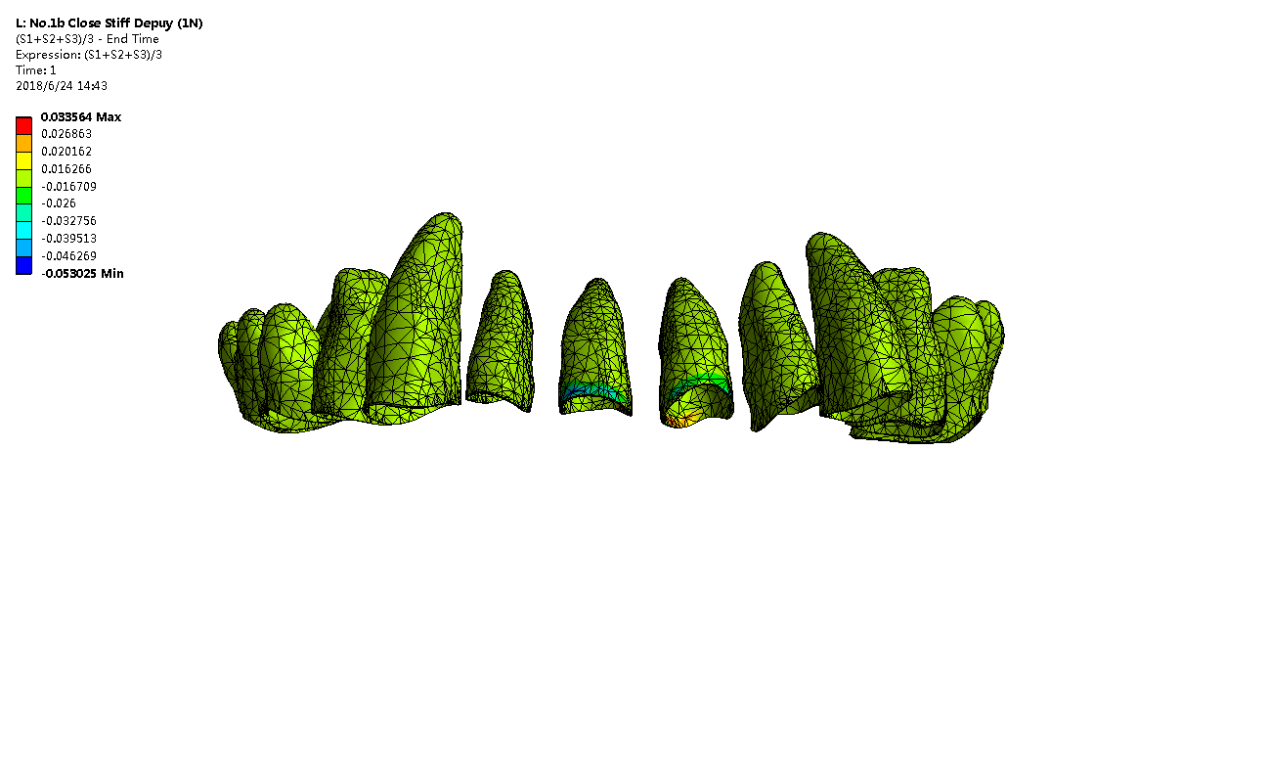

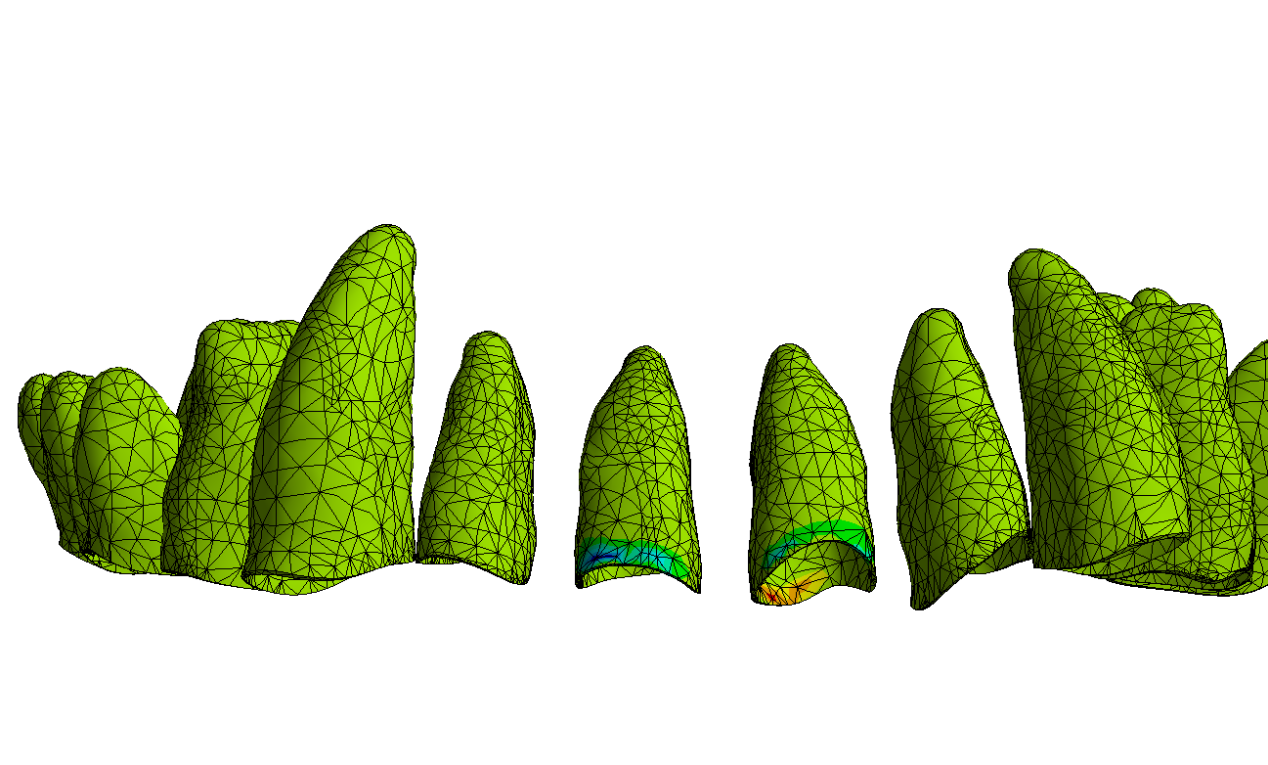

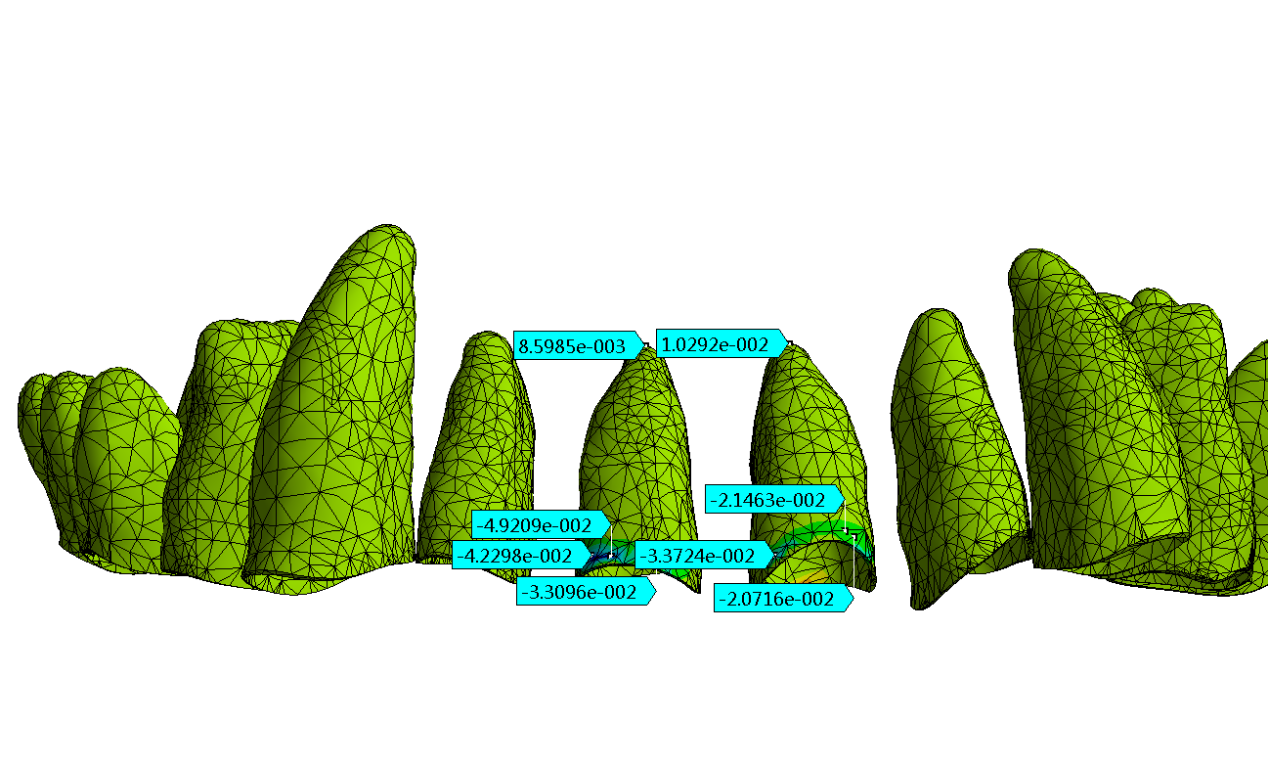


**Supplementary Fig S2: 1b group**


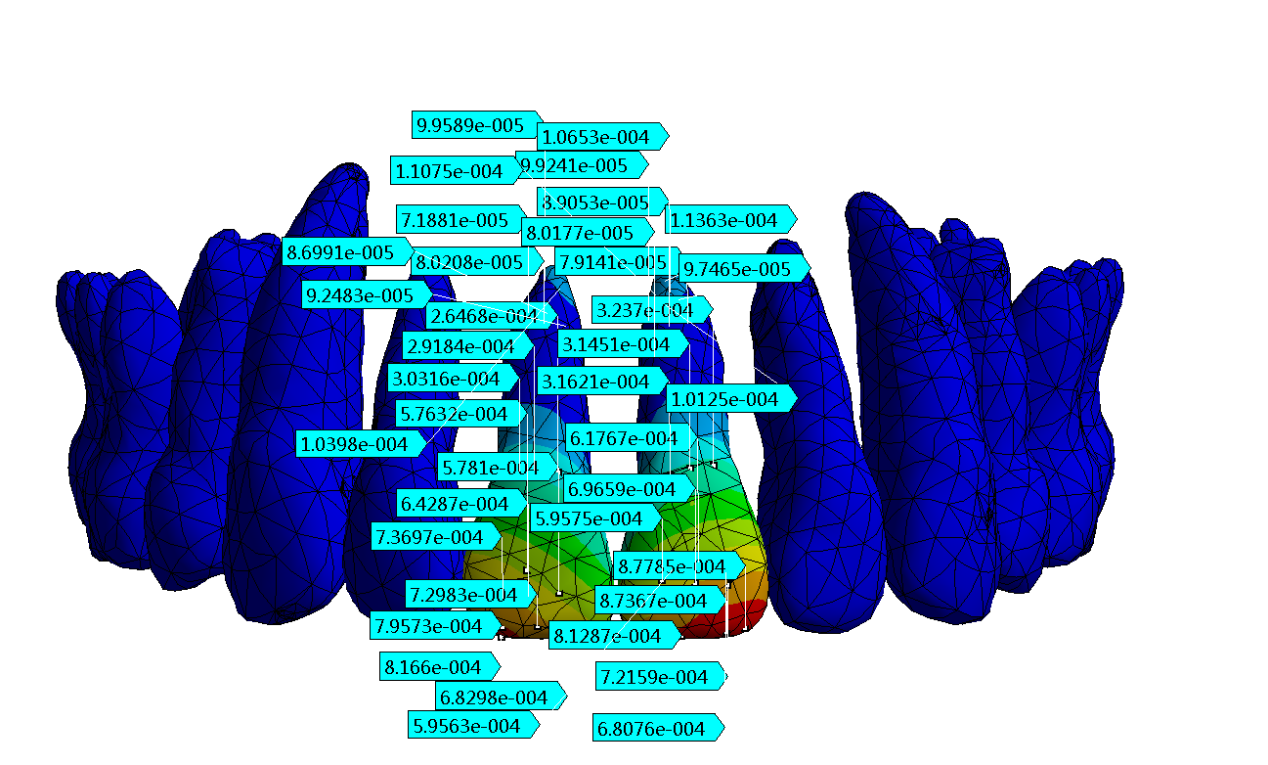

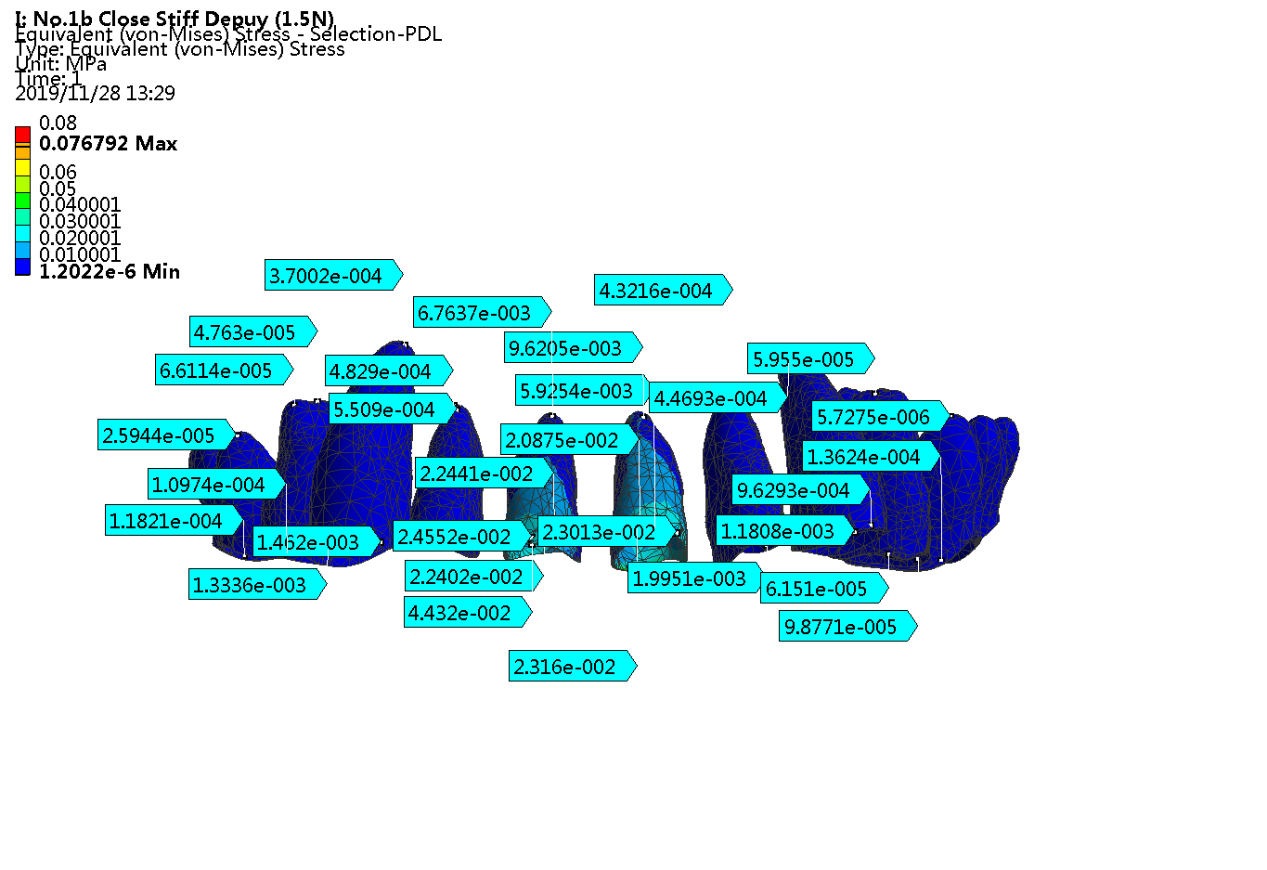

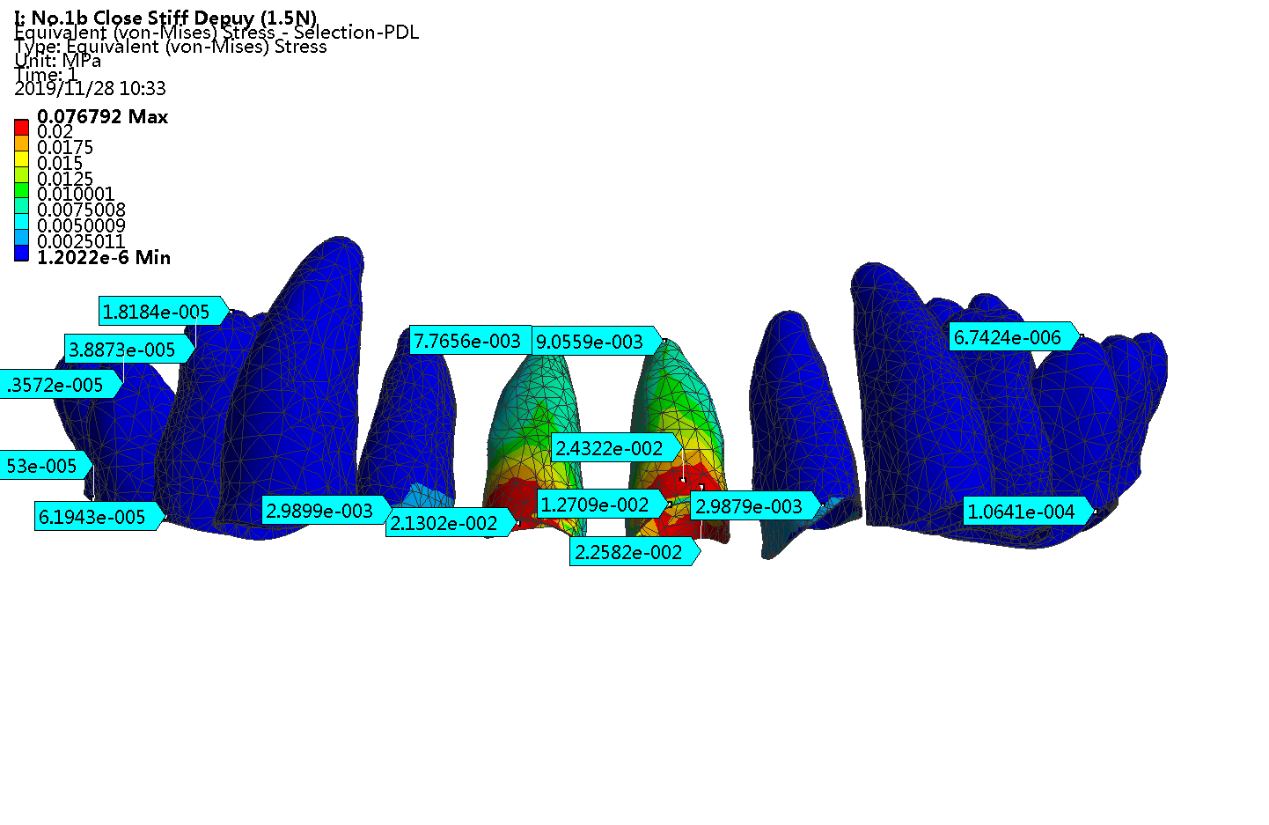

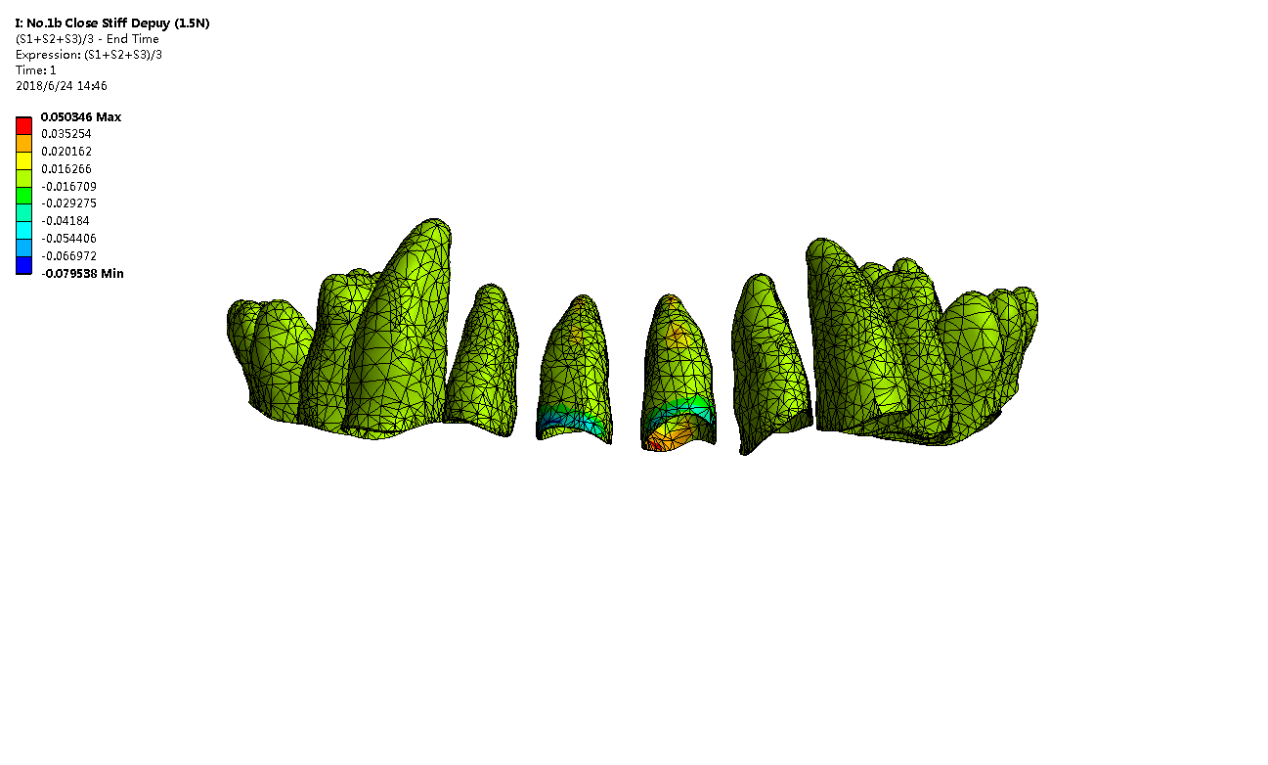

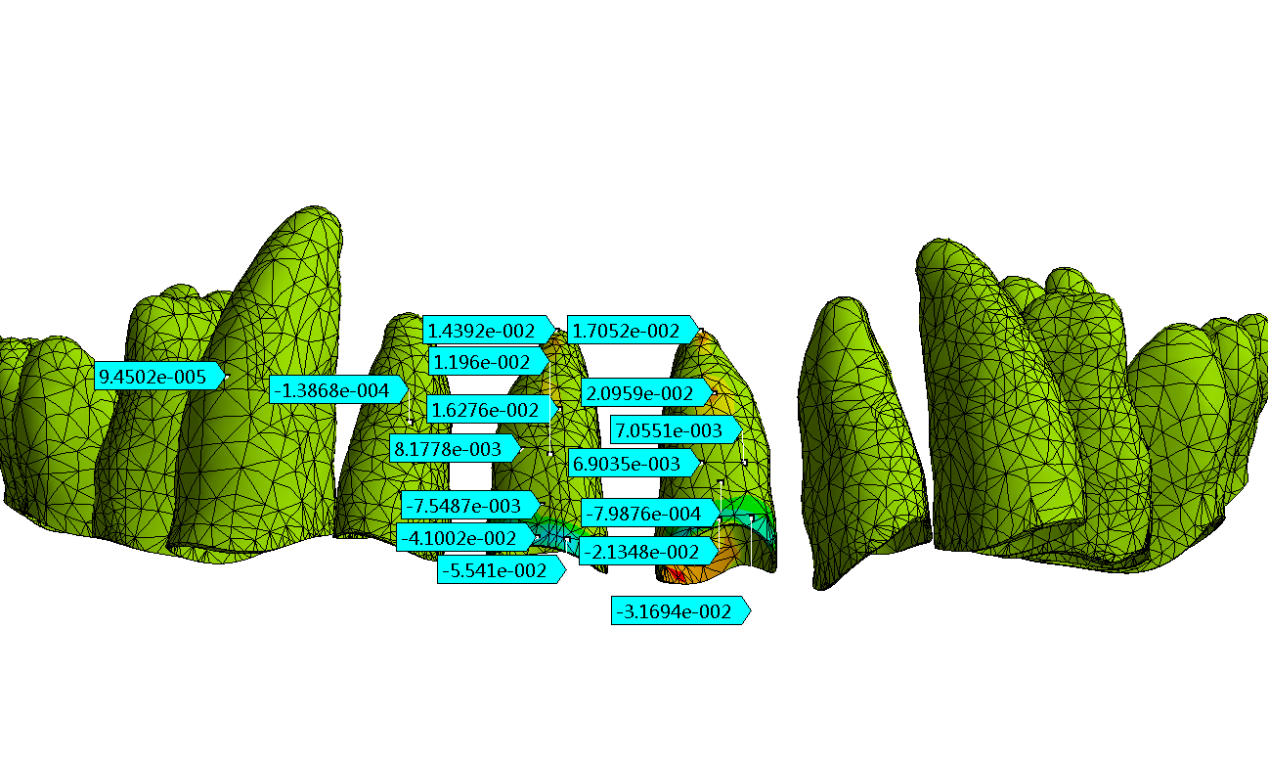

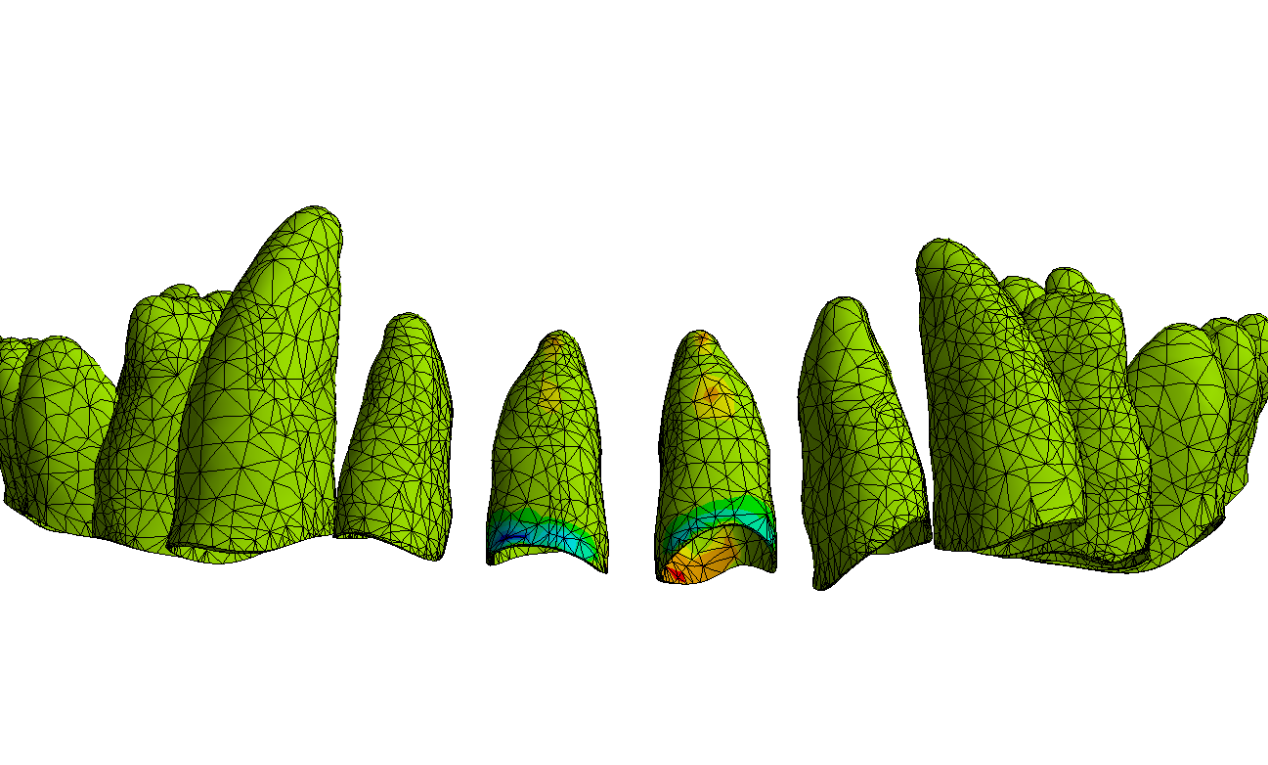


**Supplementary Fig S3: 1c group**


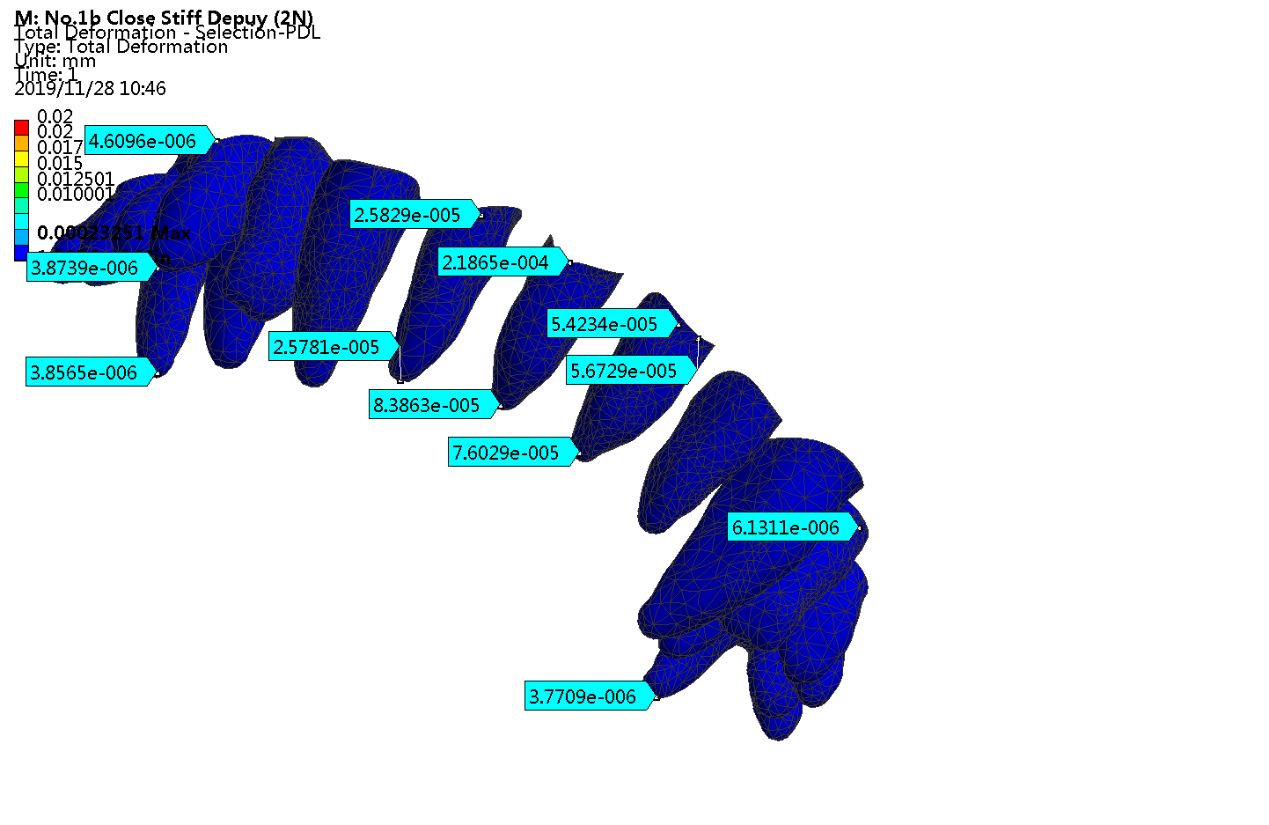

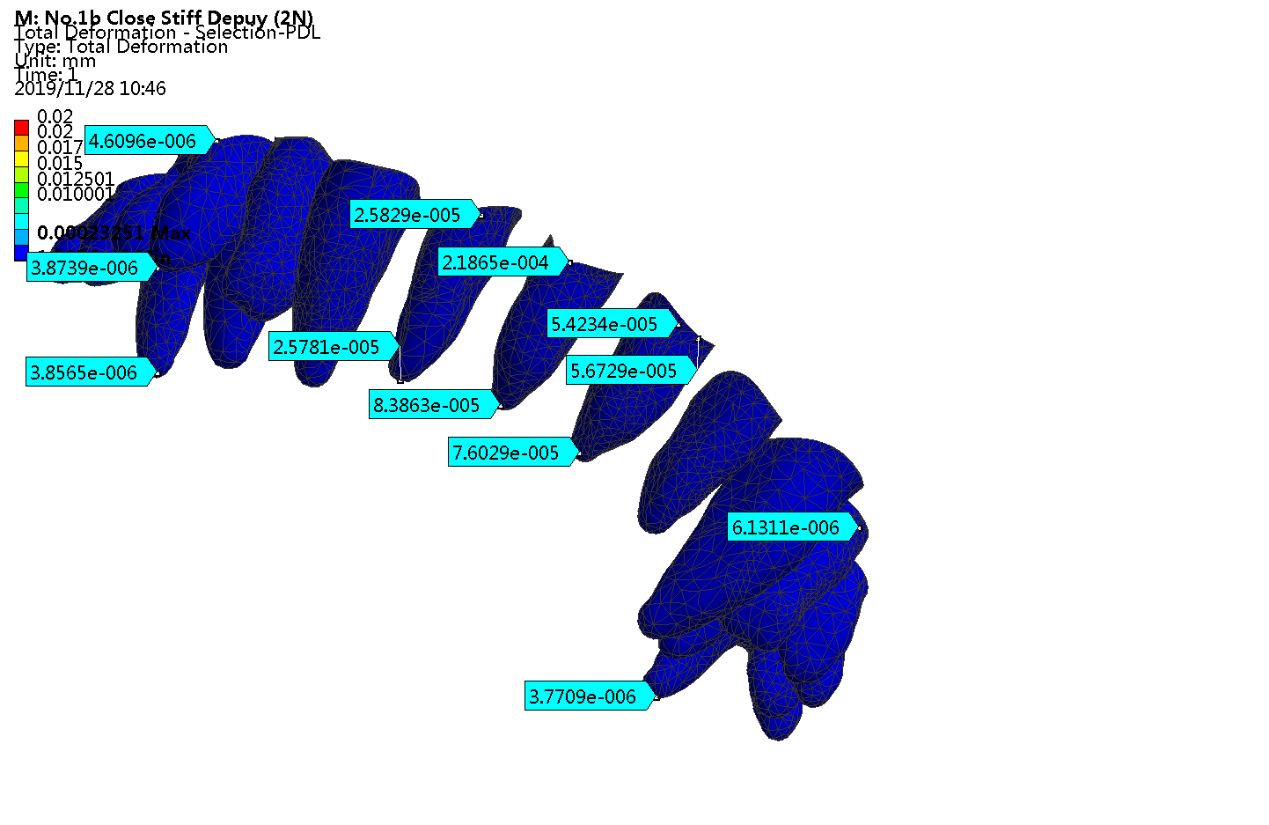

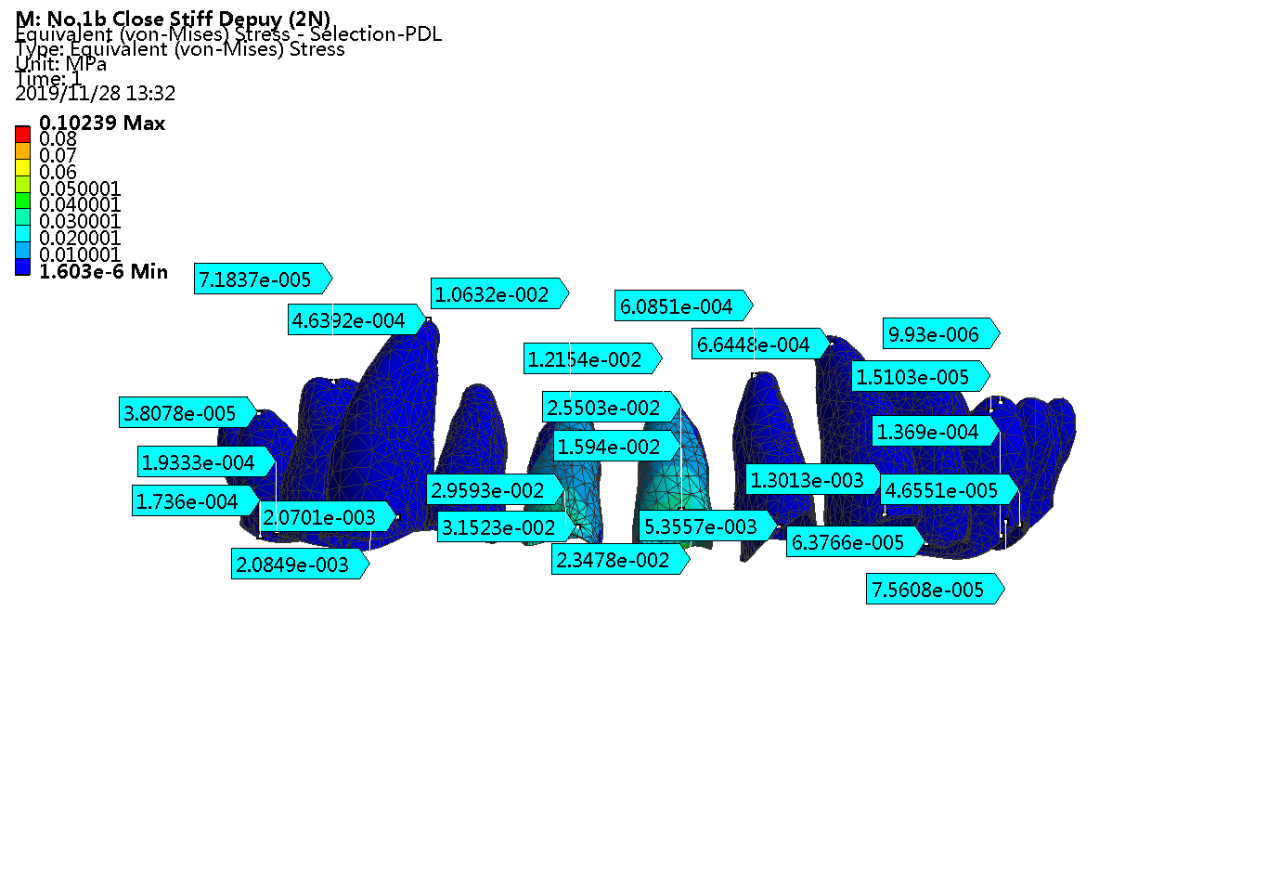

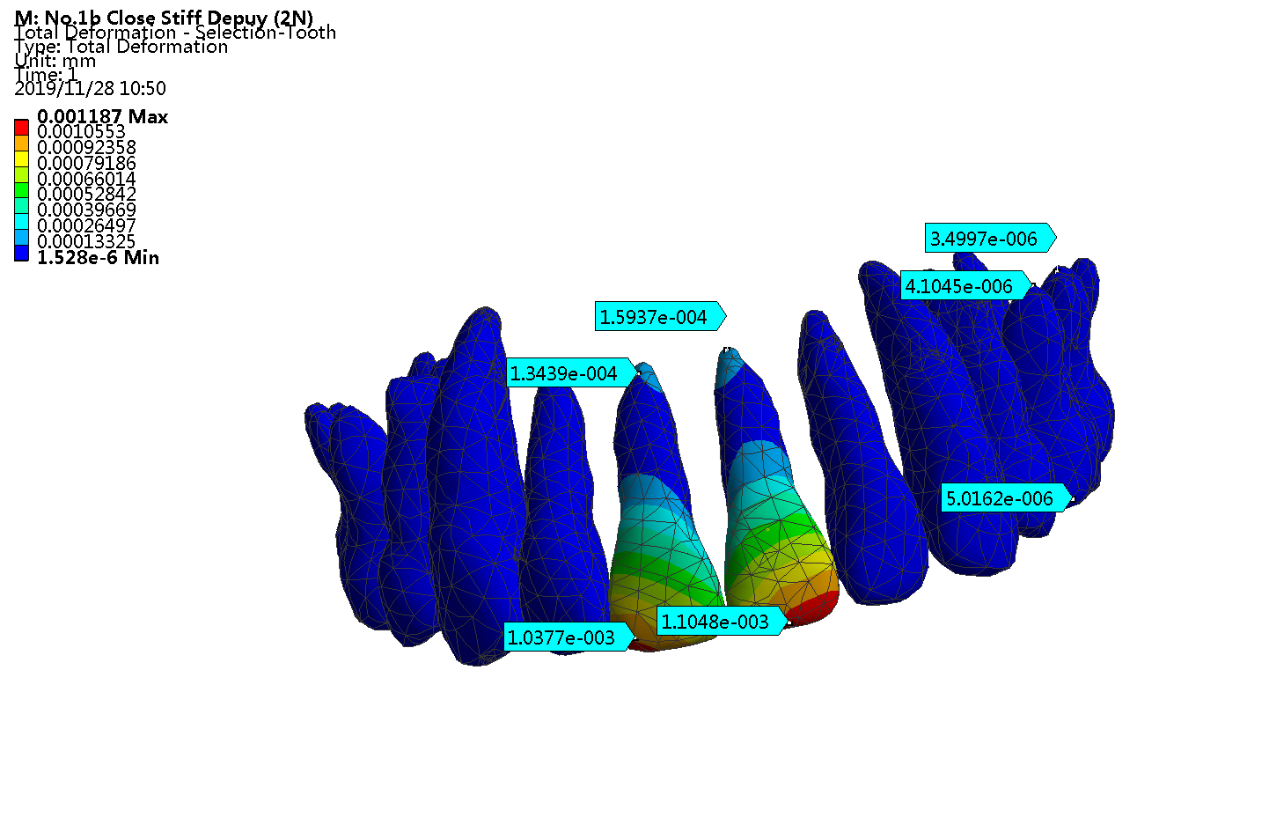

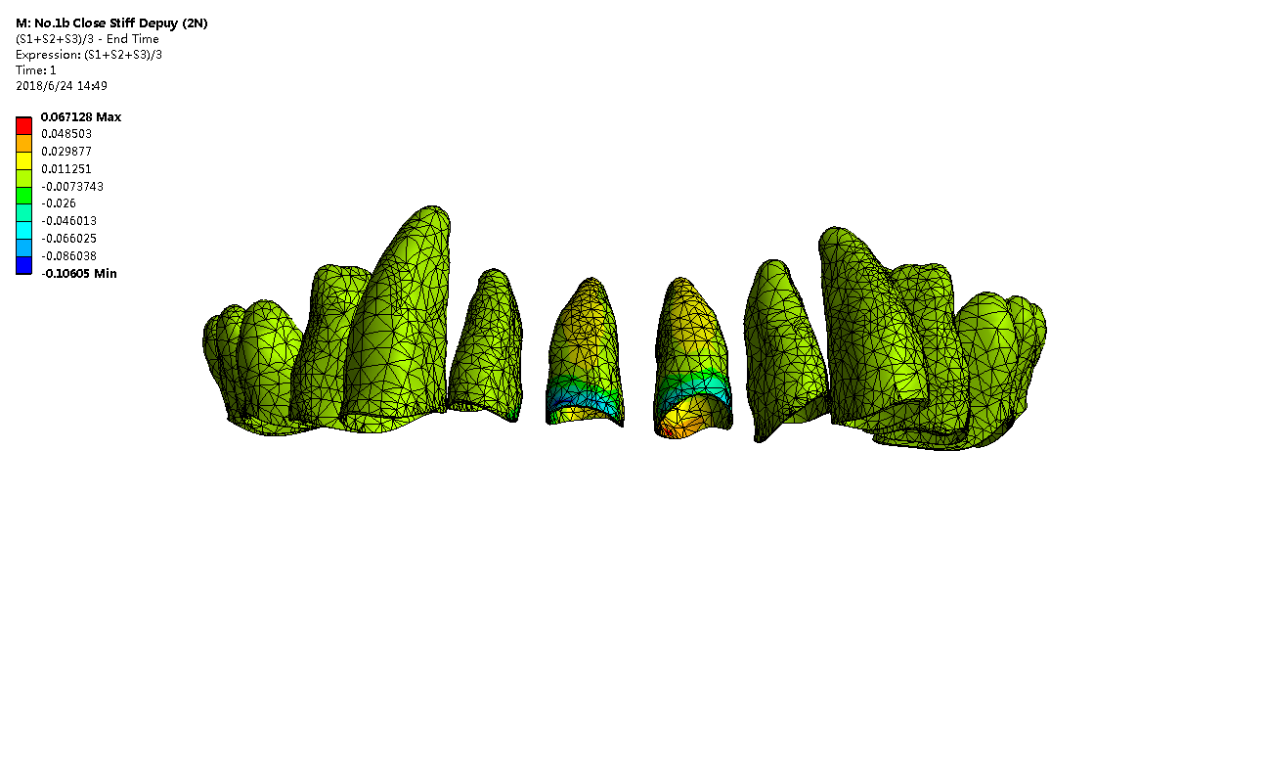

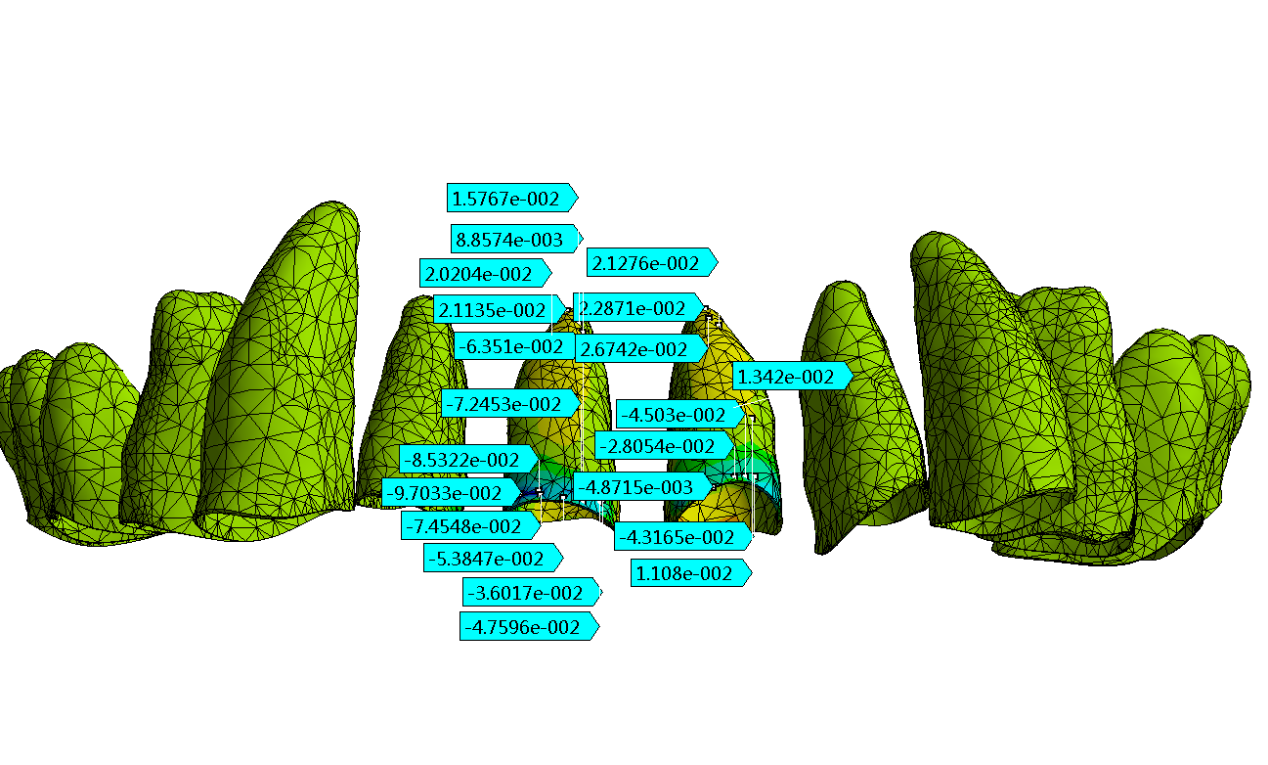

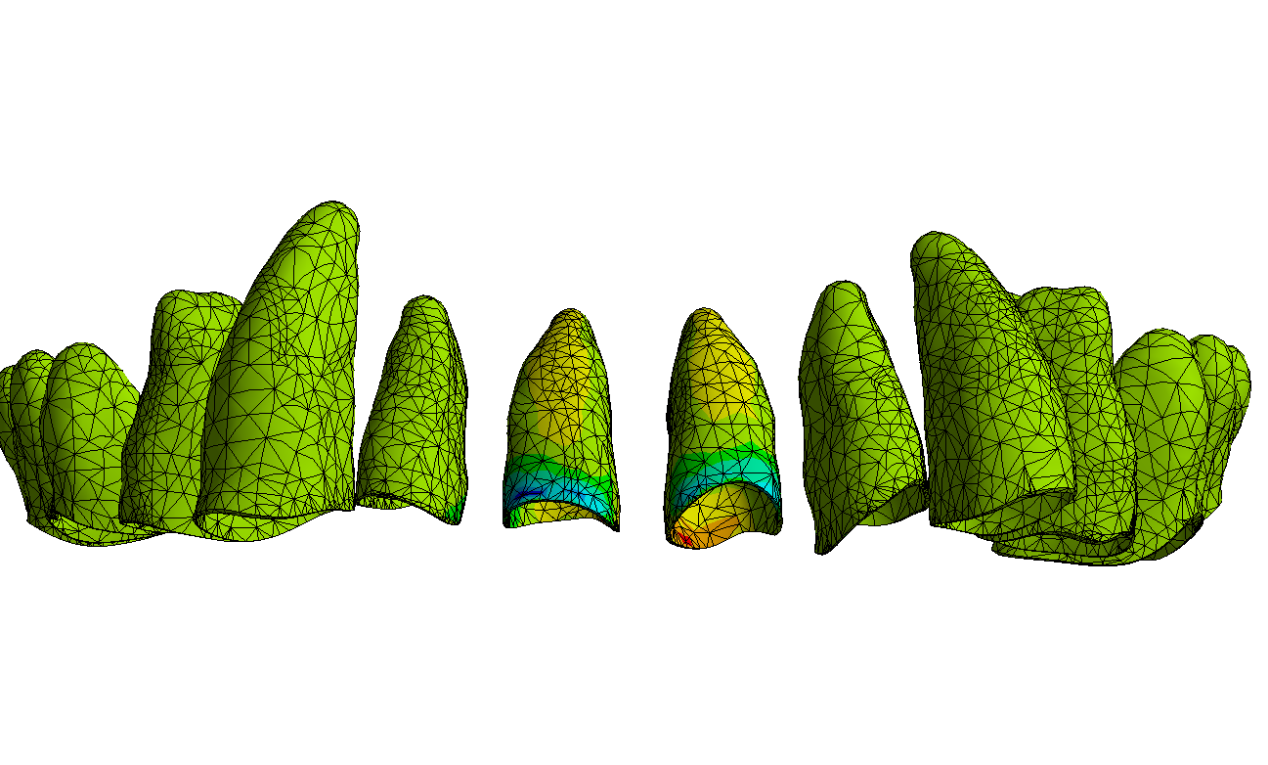

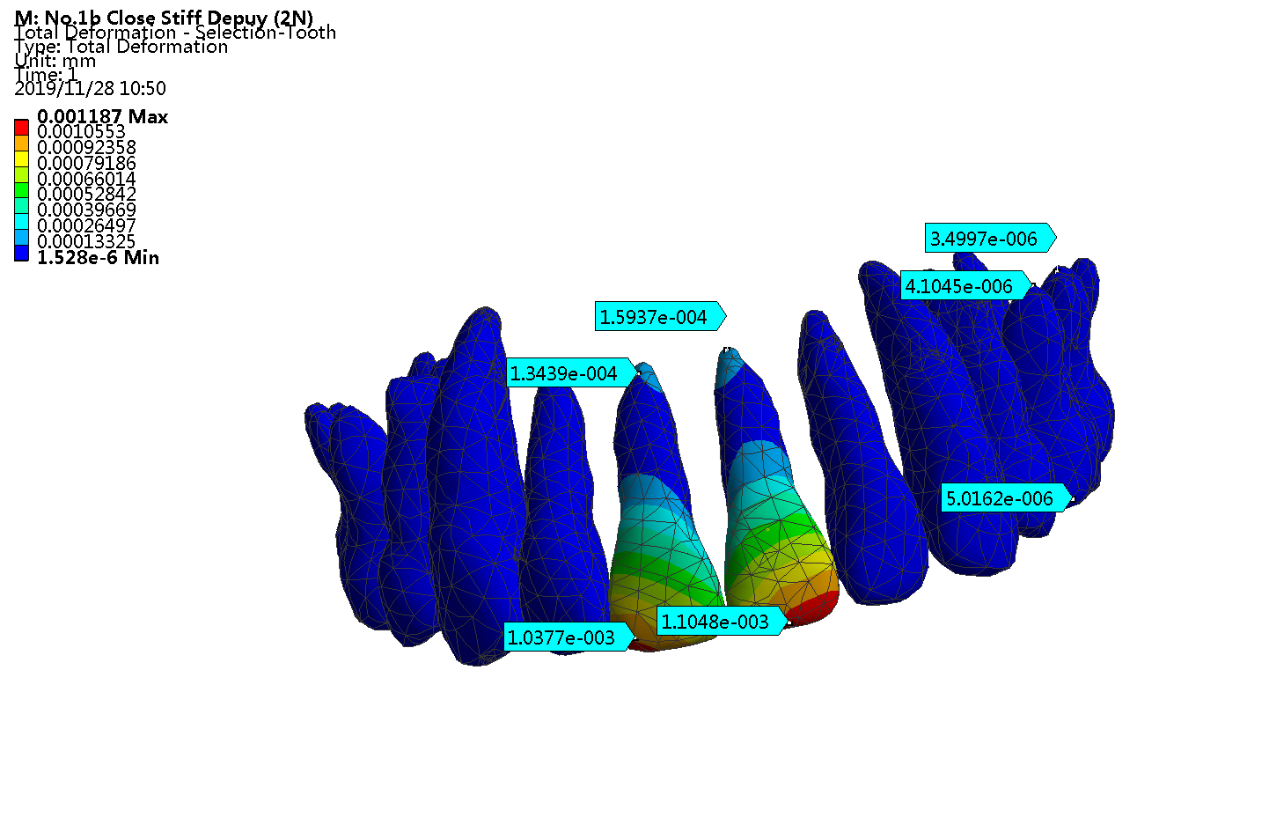
**Supplementary Fig S4: 1d group**

**2 group**


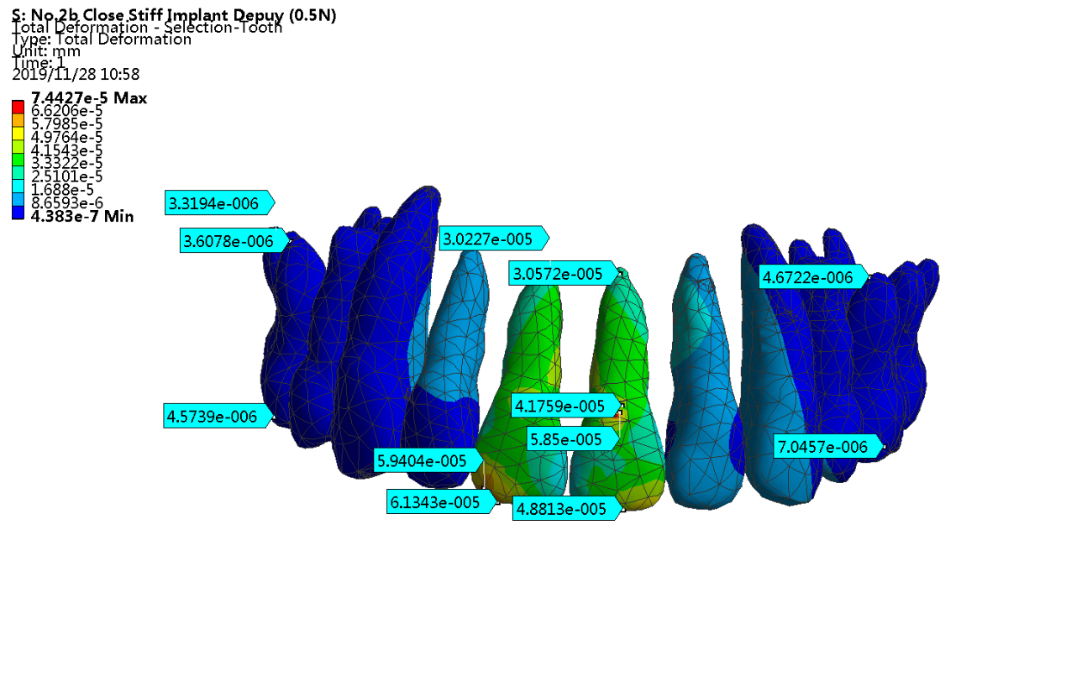

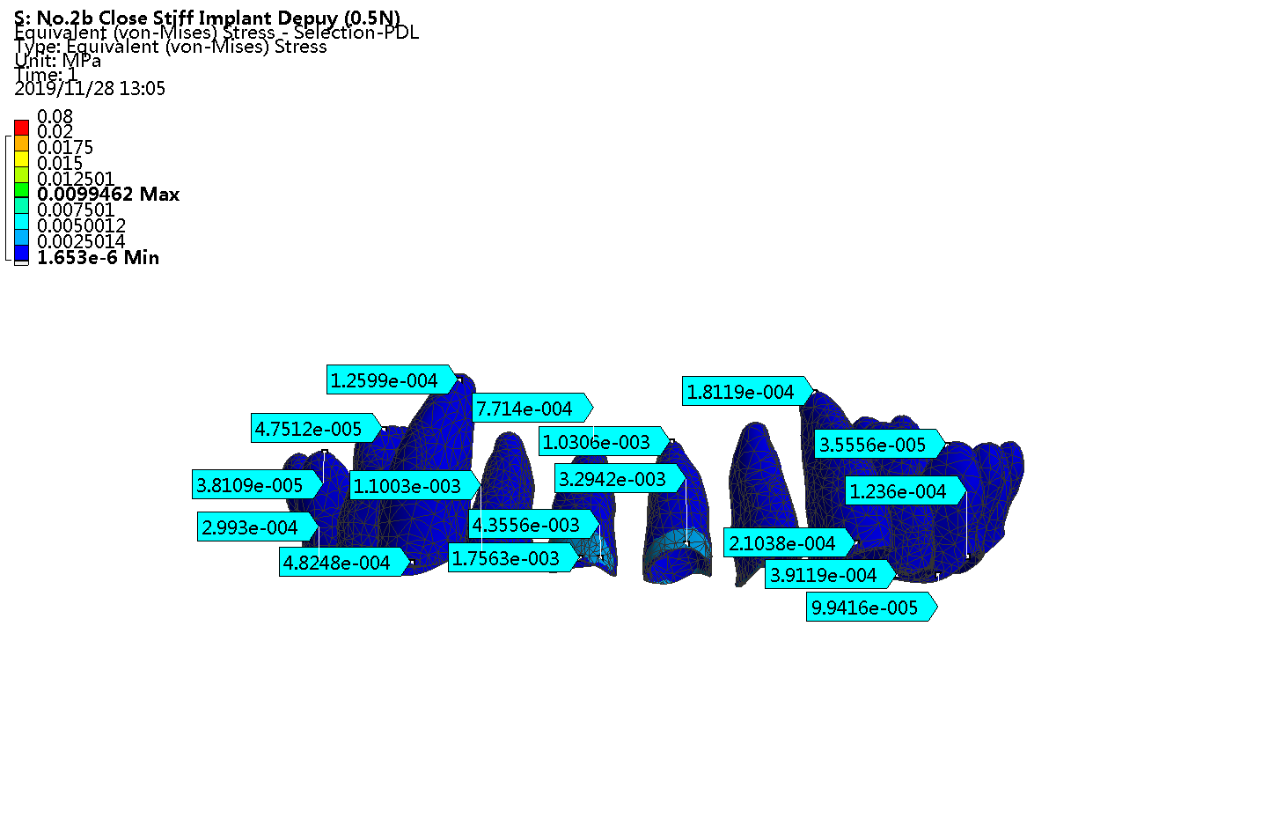

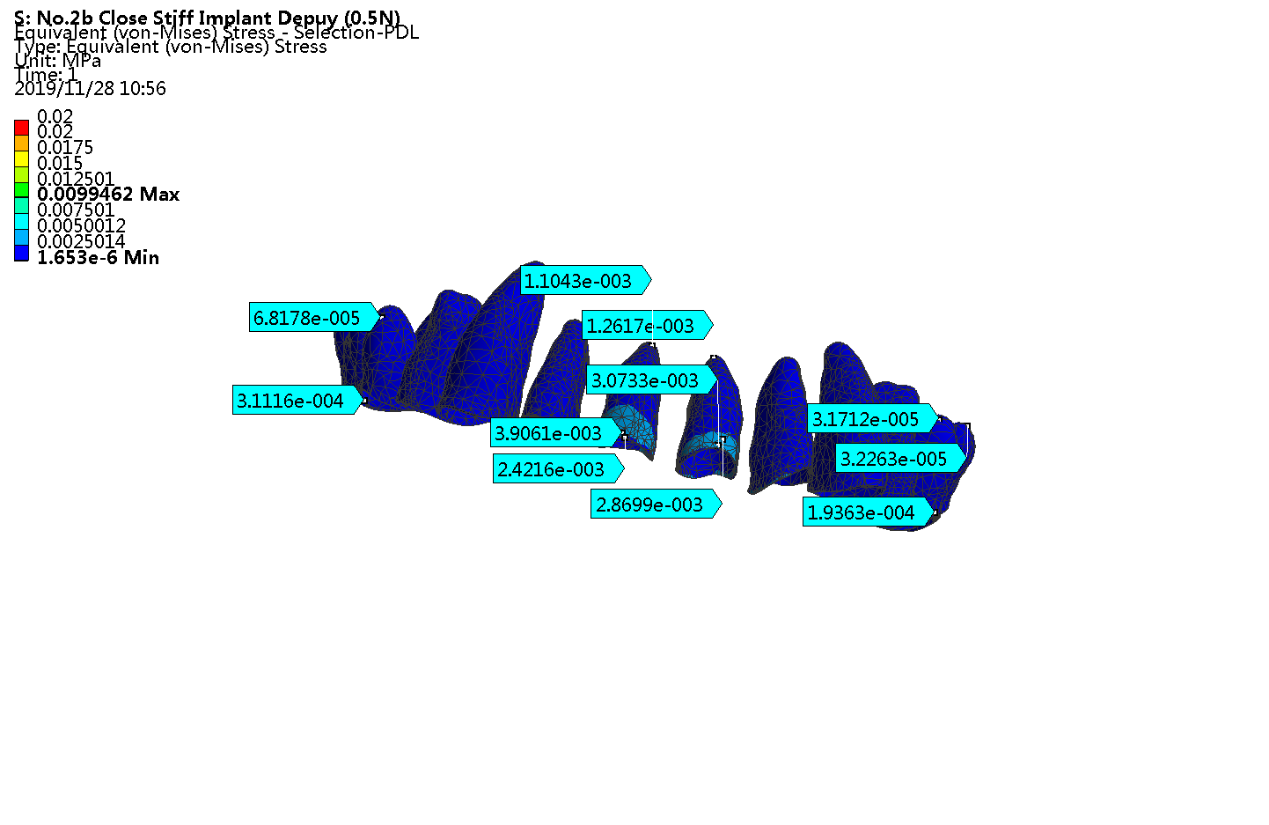

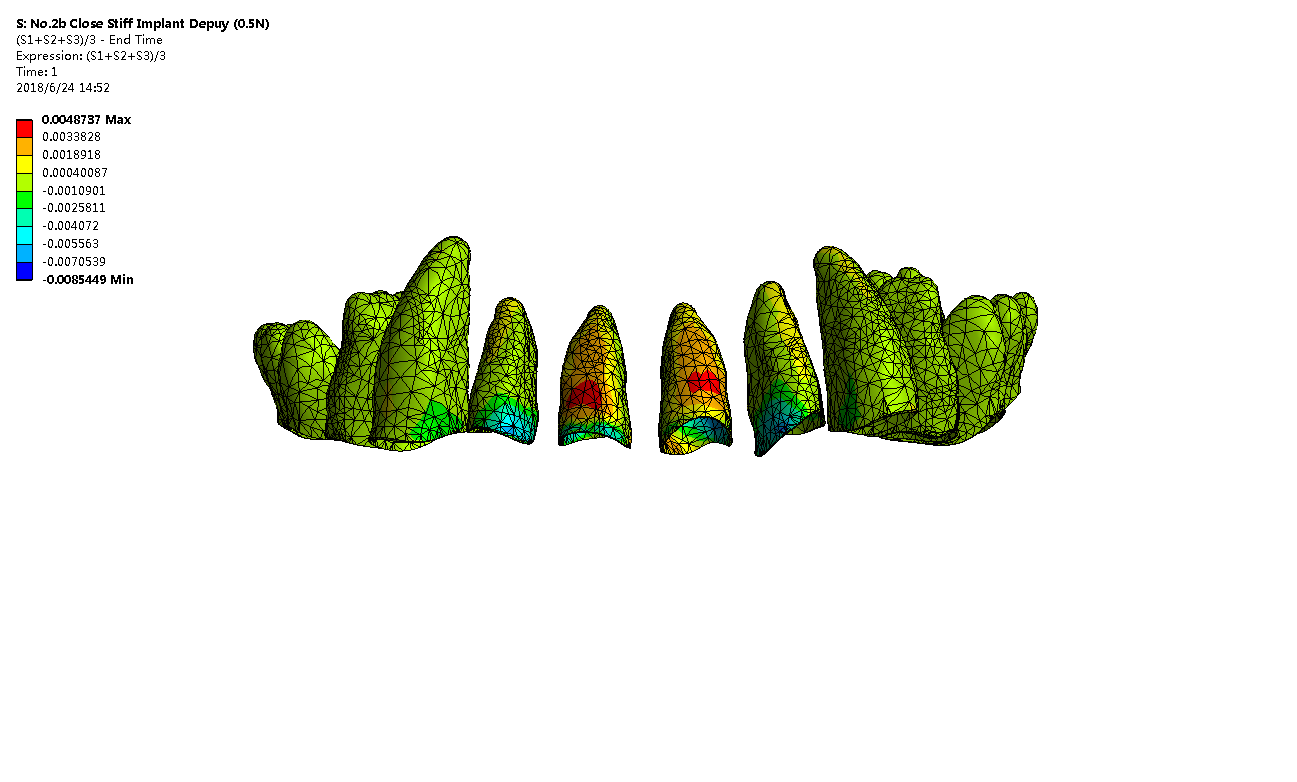

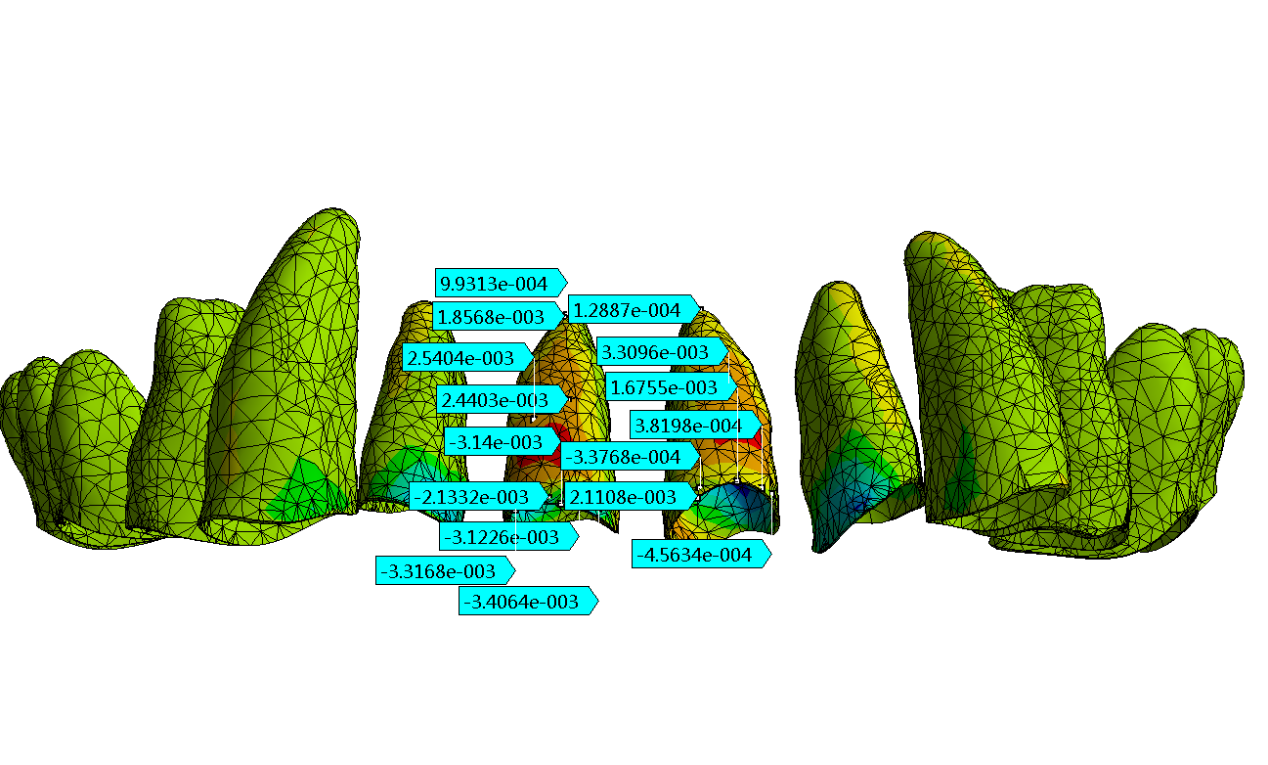

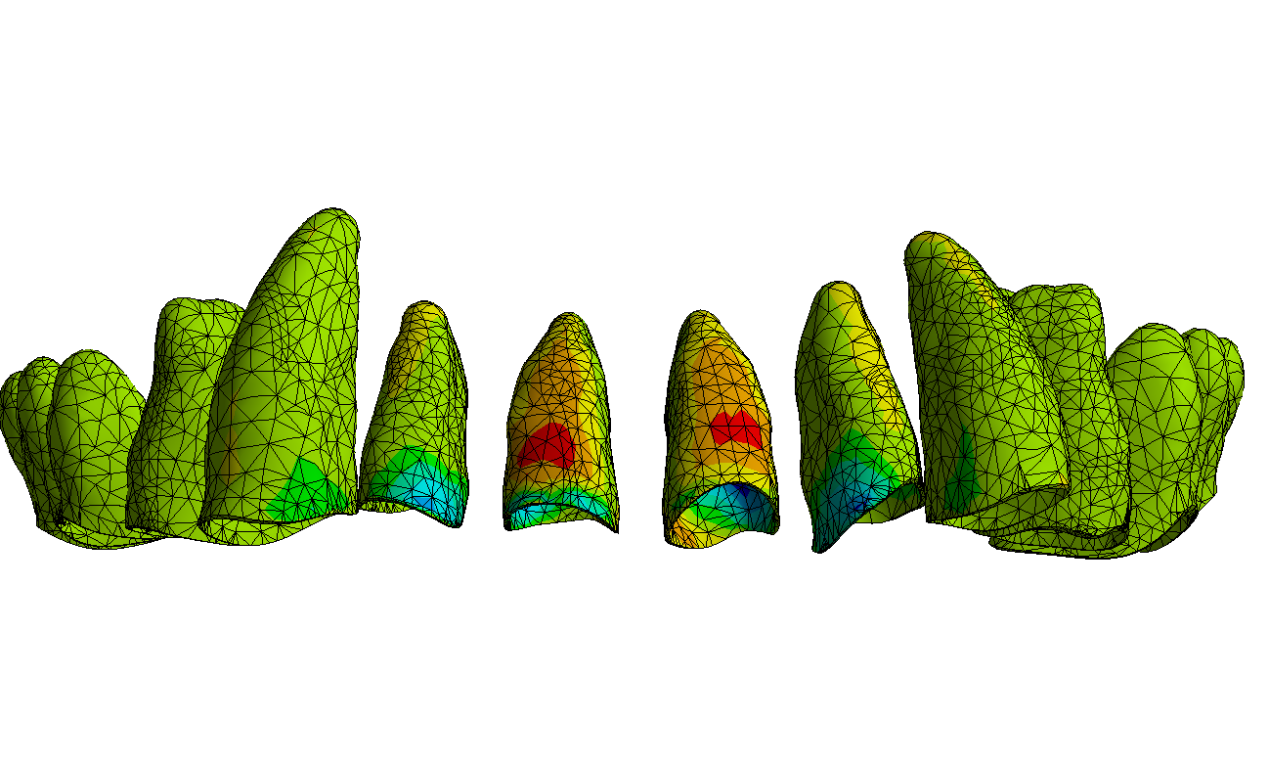

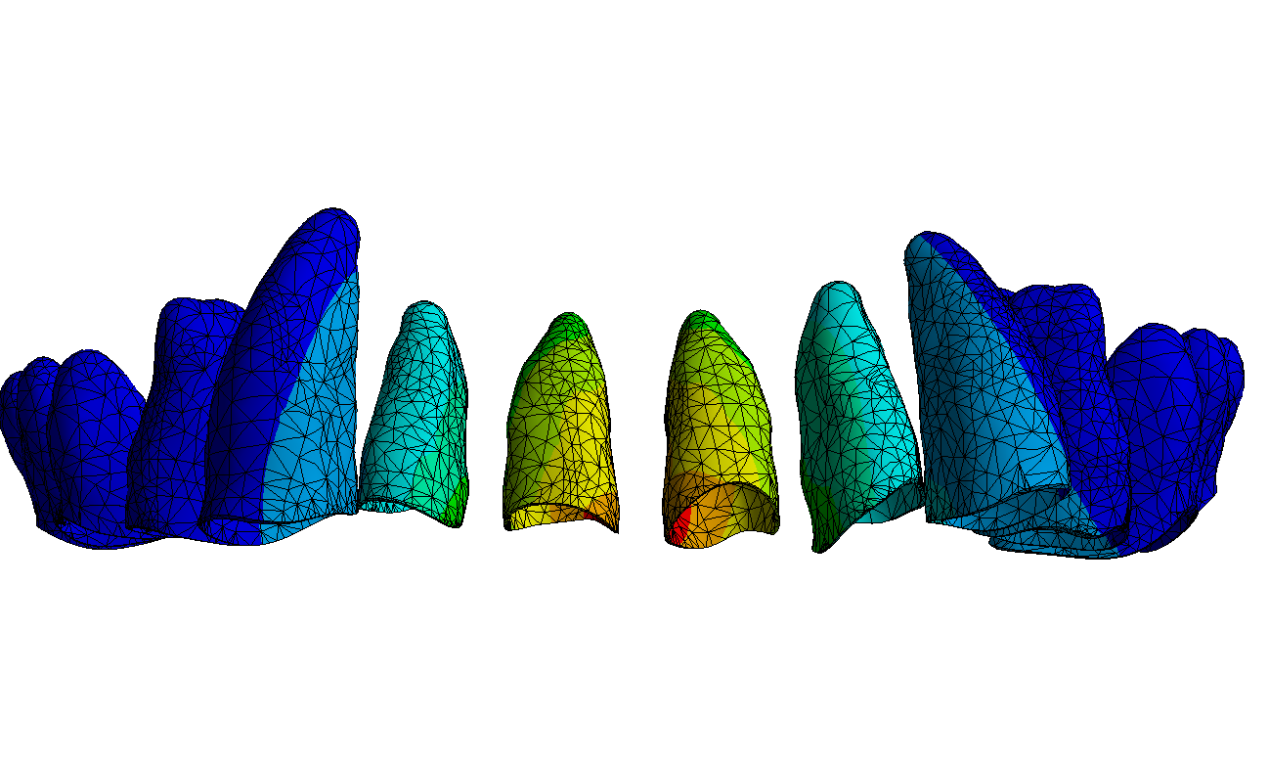


**Supplementary Fig S5: 2a group**


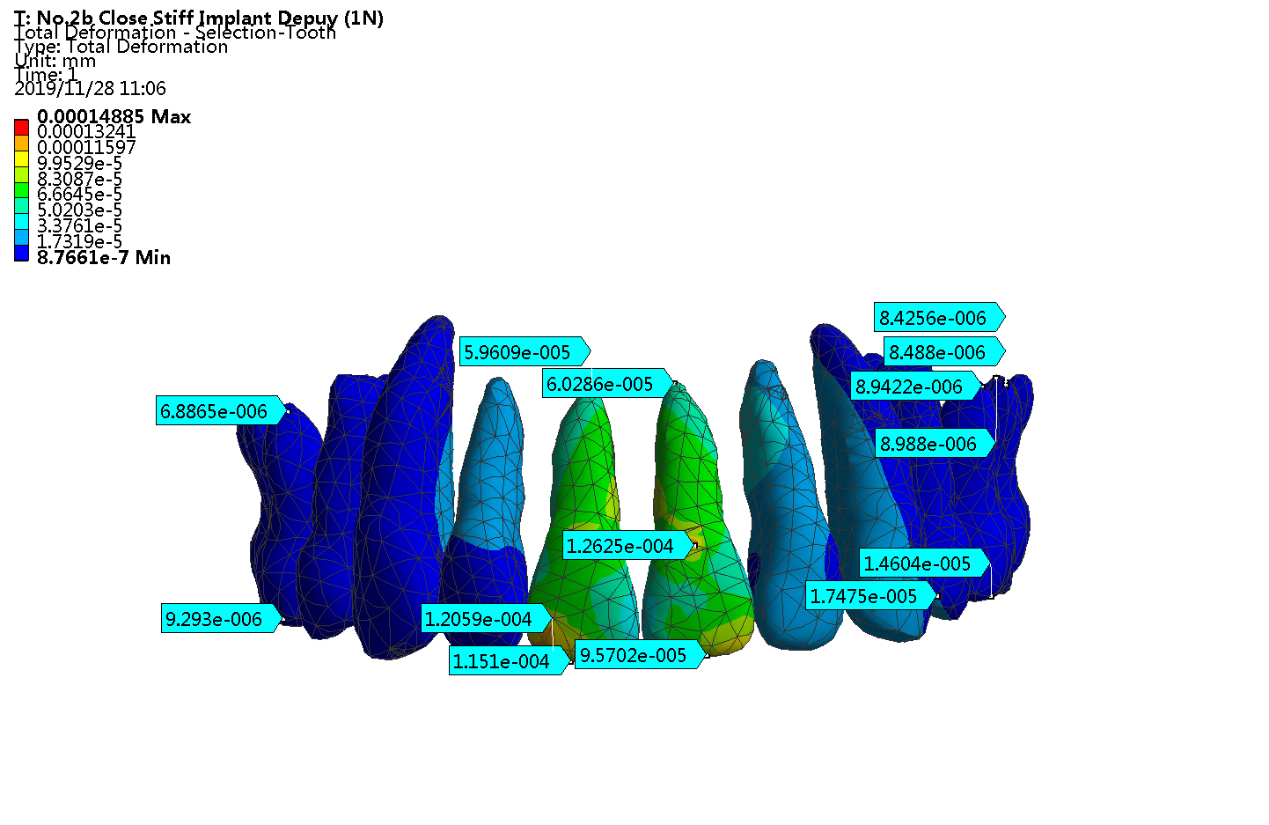

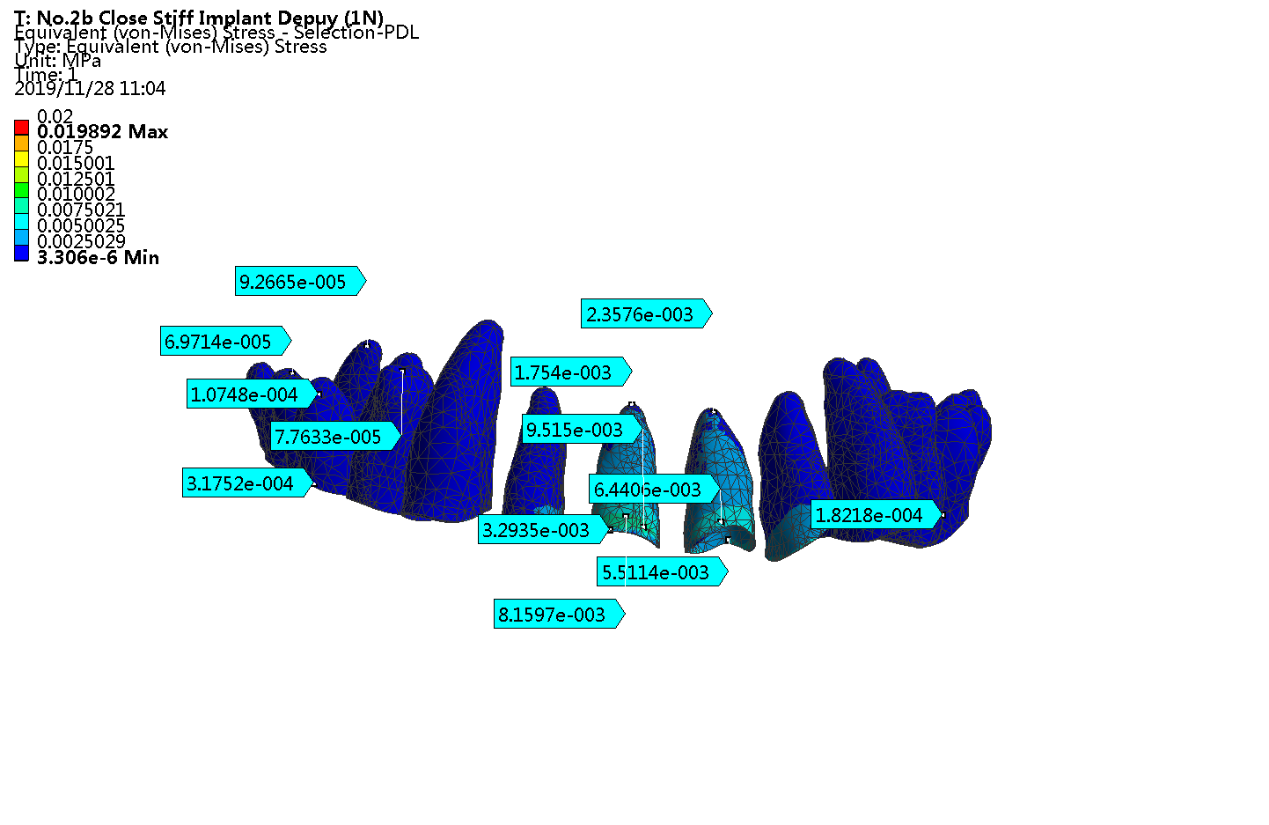

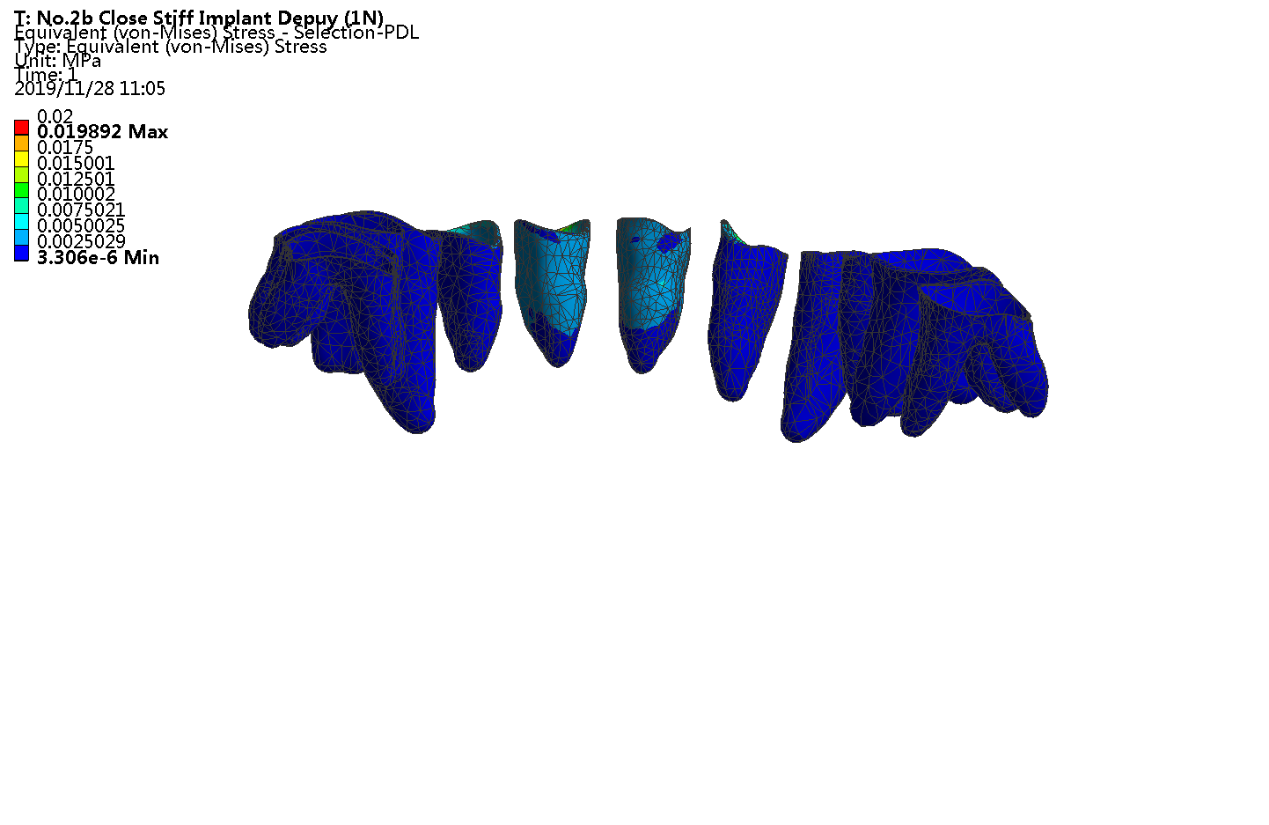

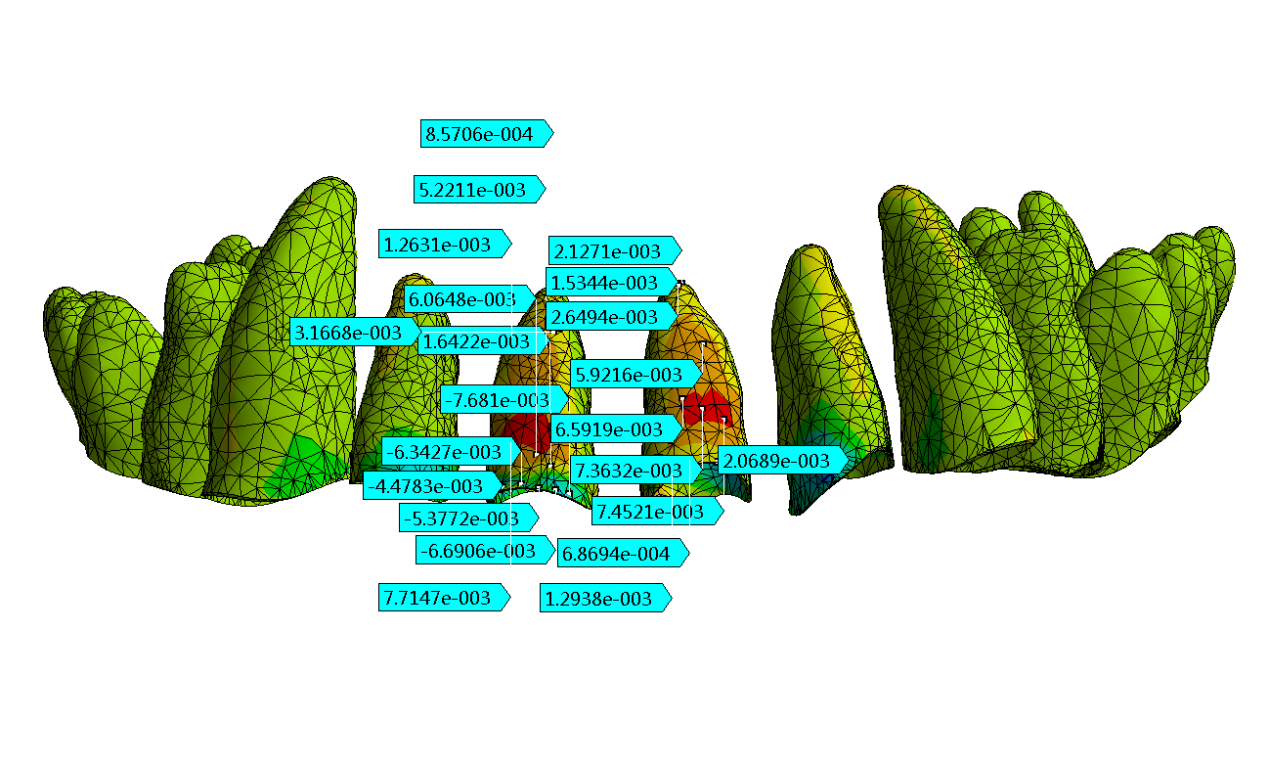

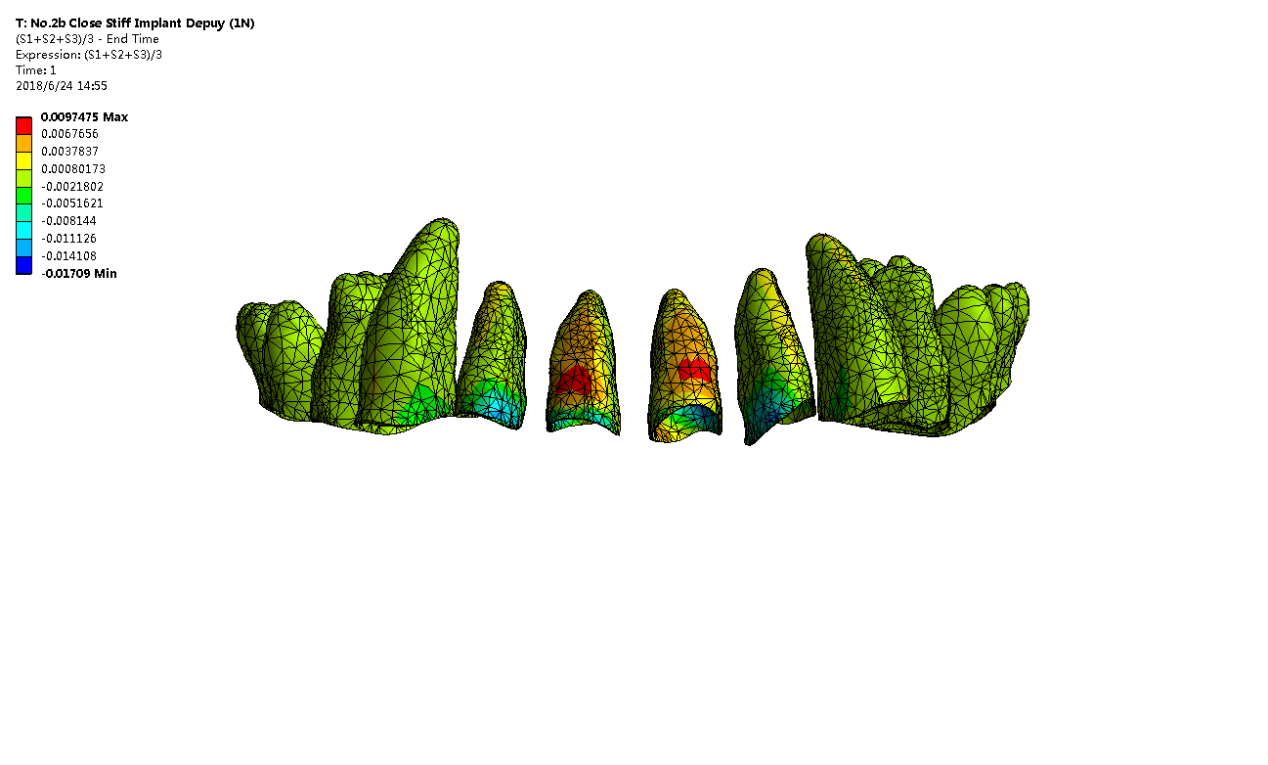

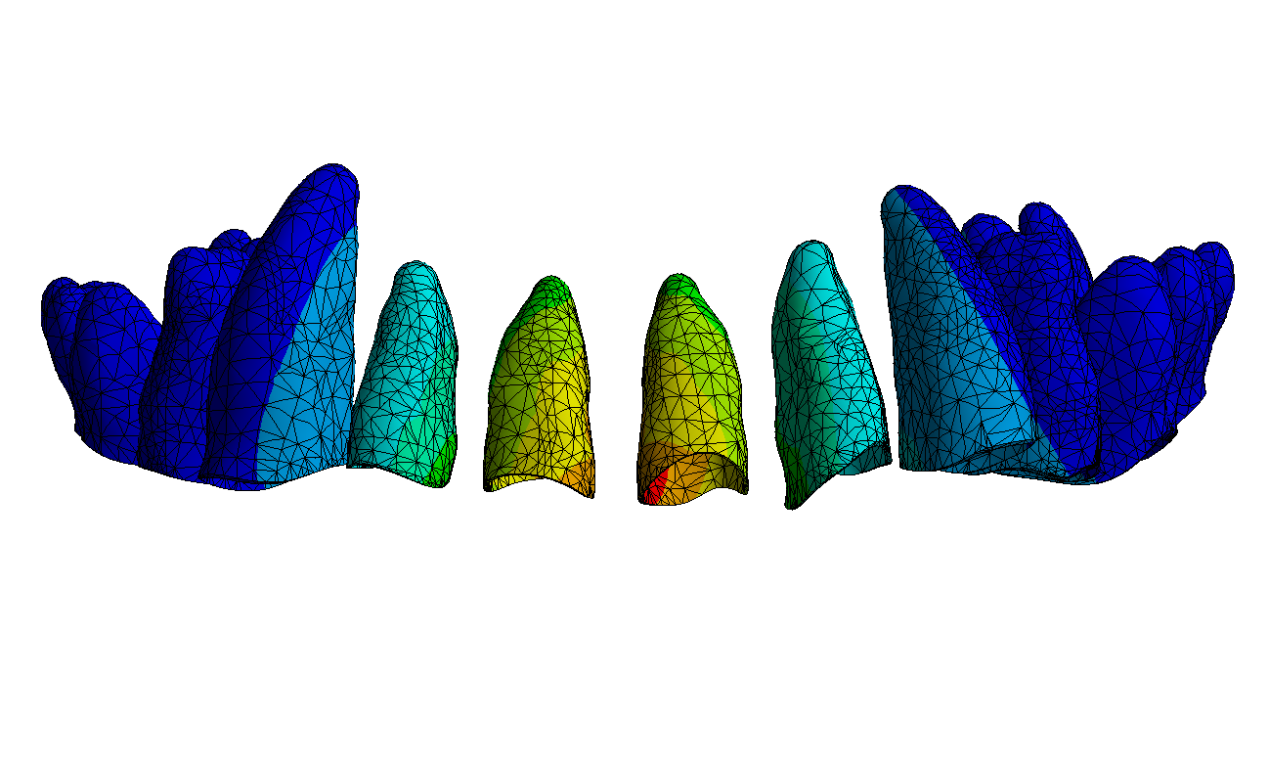


**Supplementary Figs S6: 2b group**


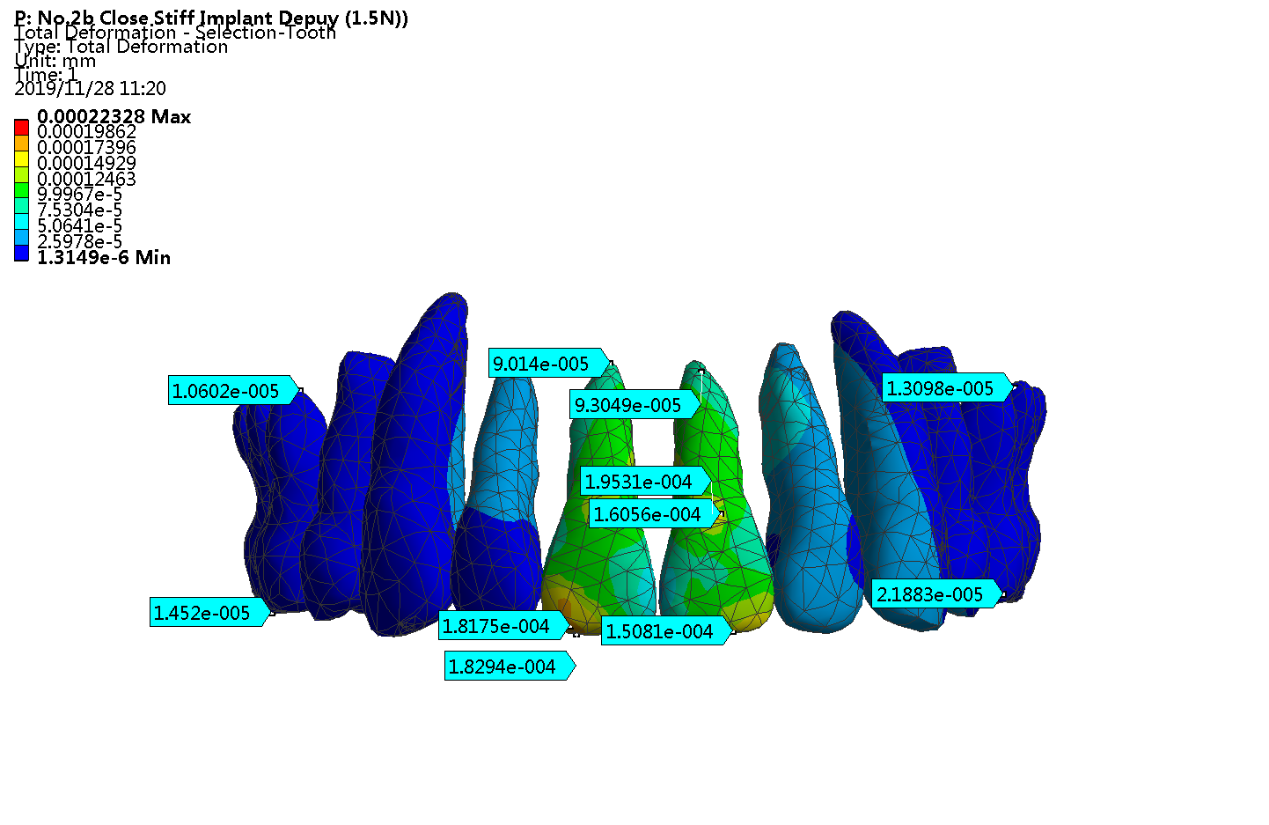

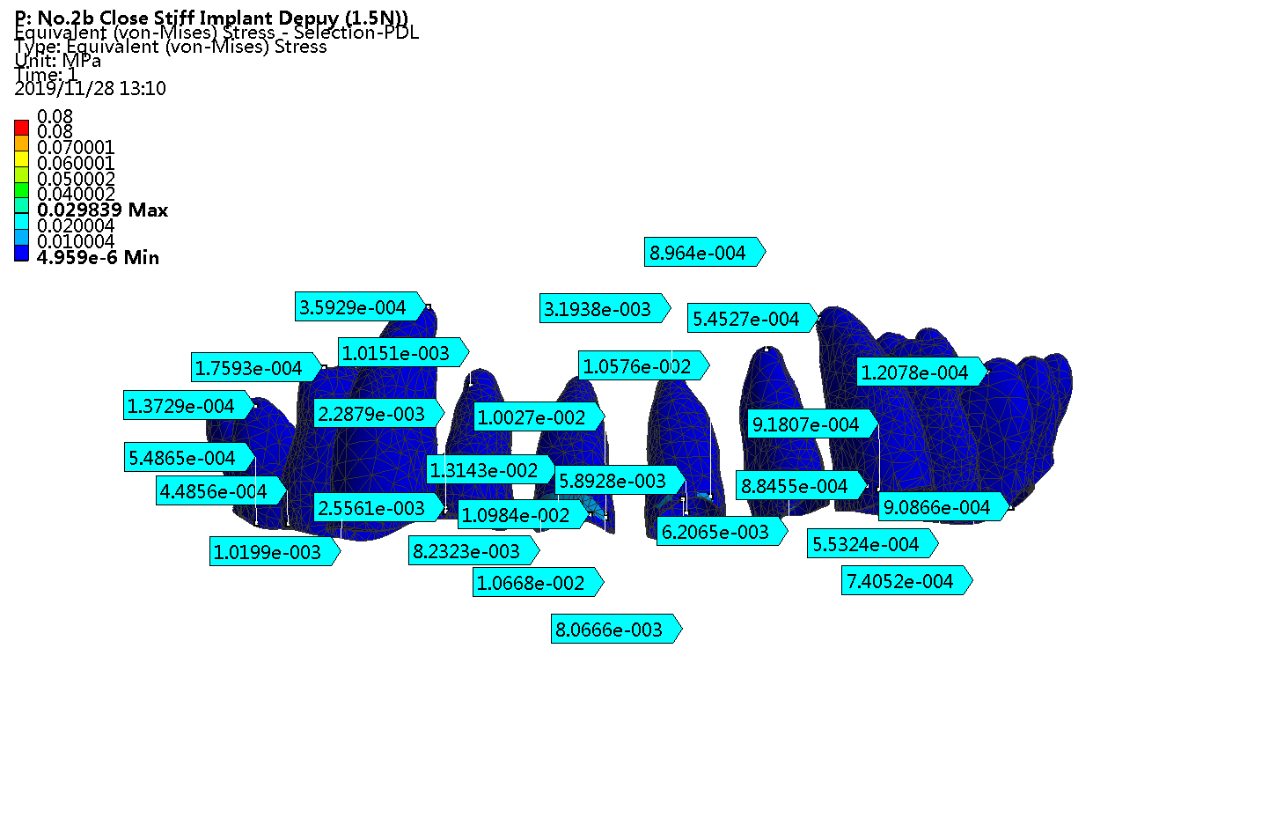

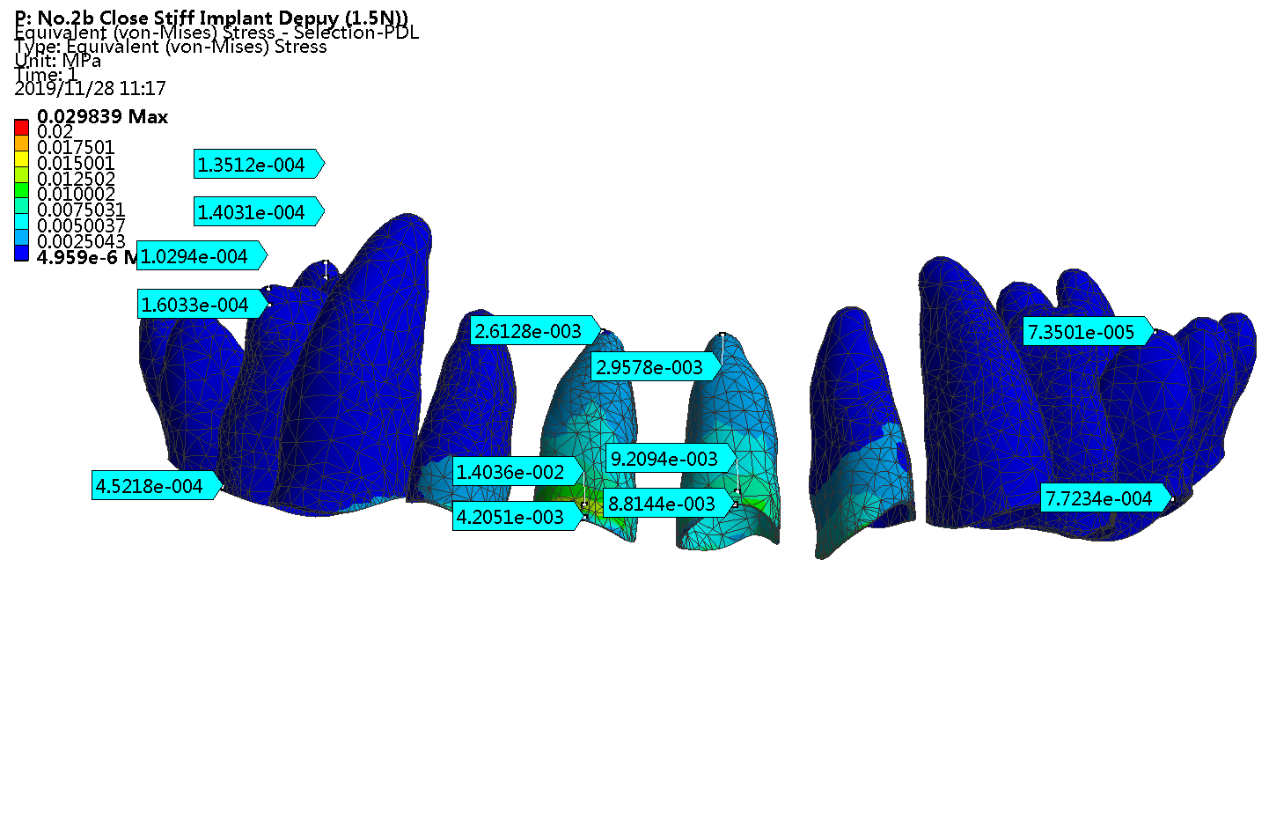

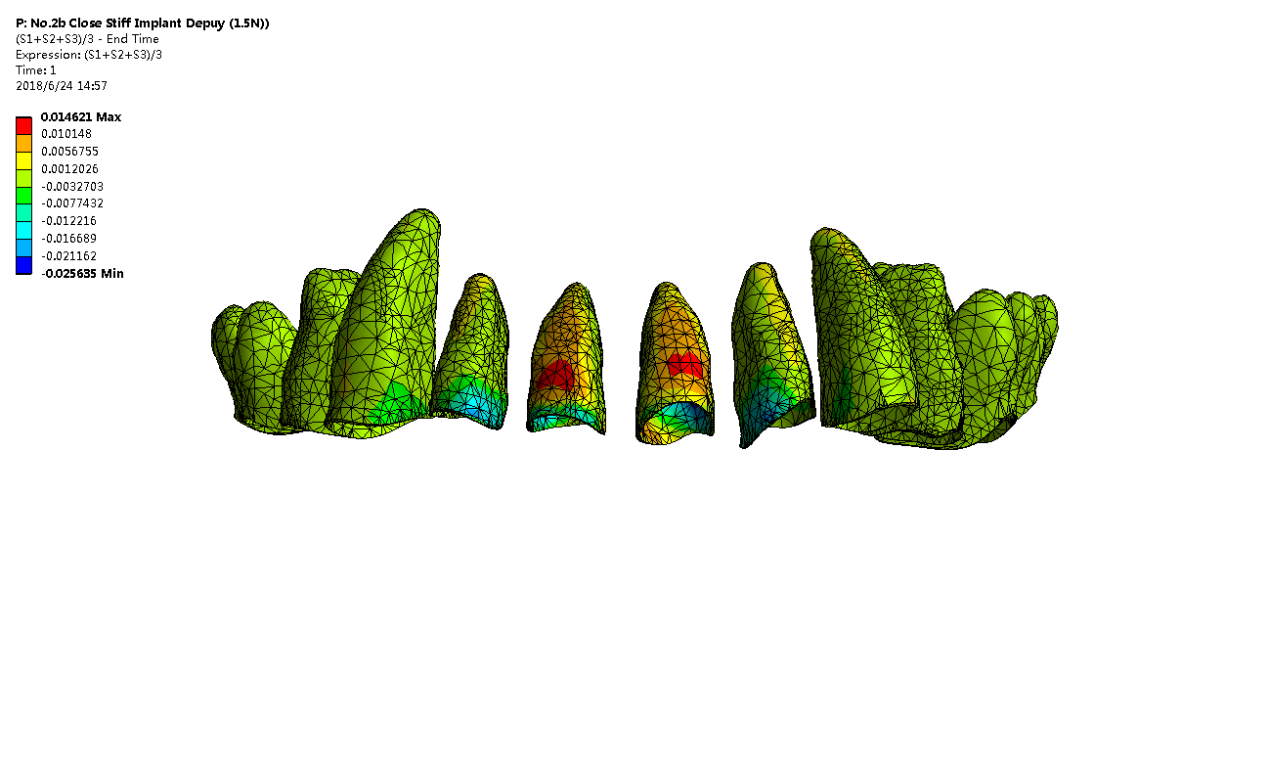

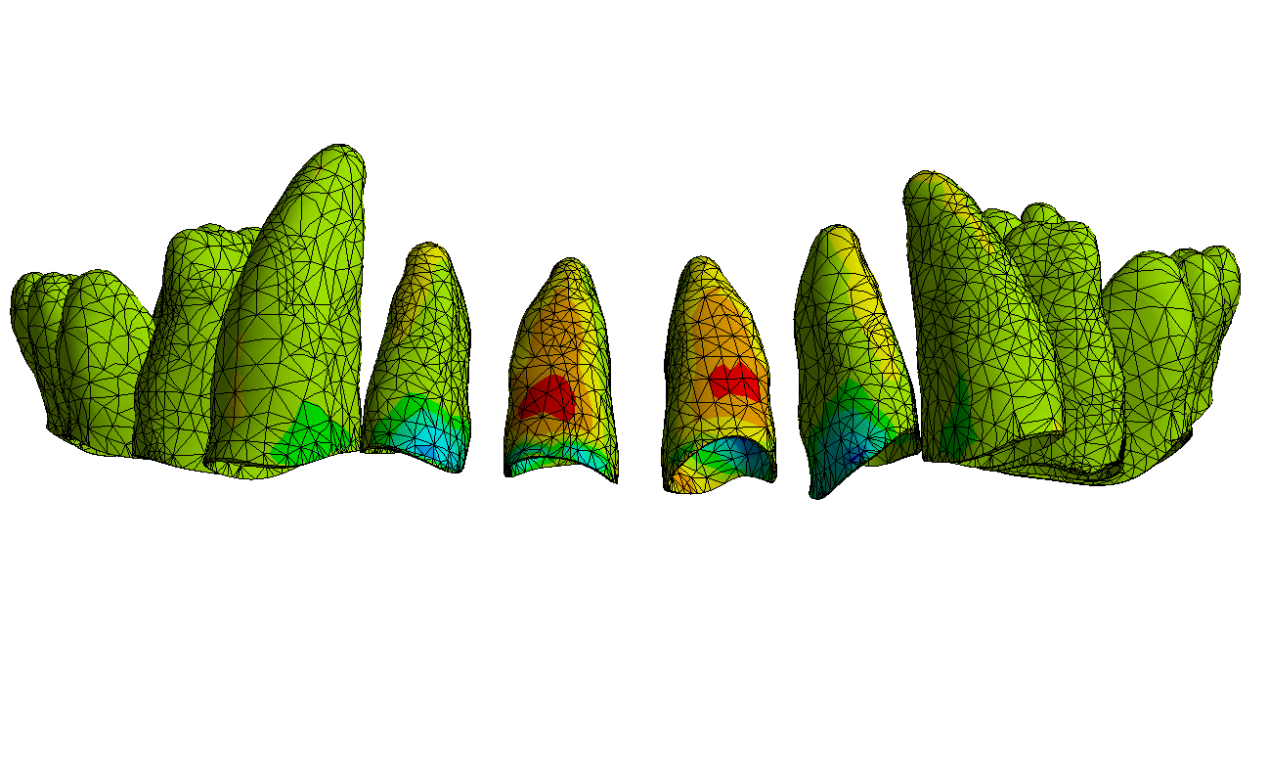


**Supplementary Figs S7: 2c group**


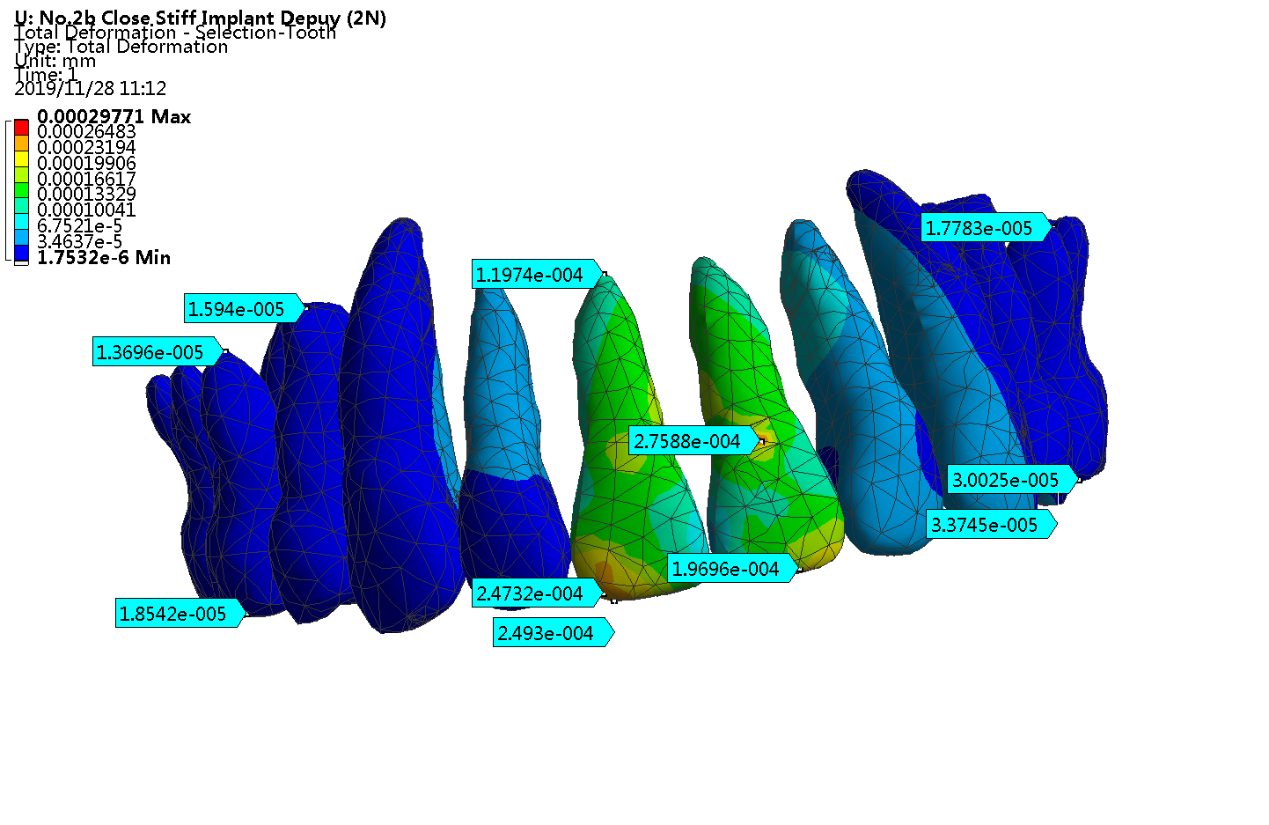

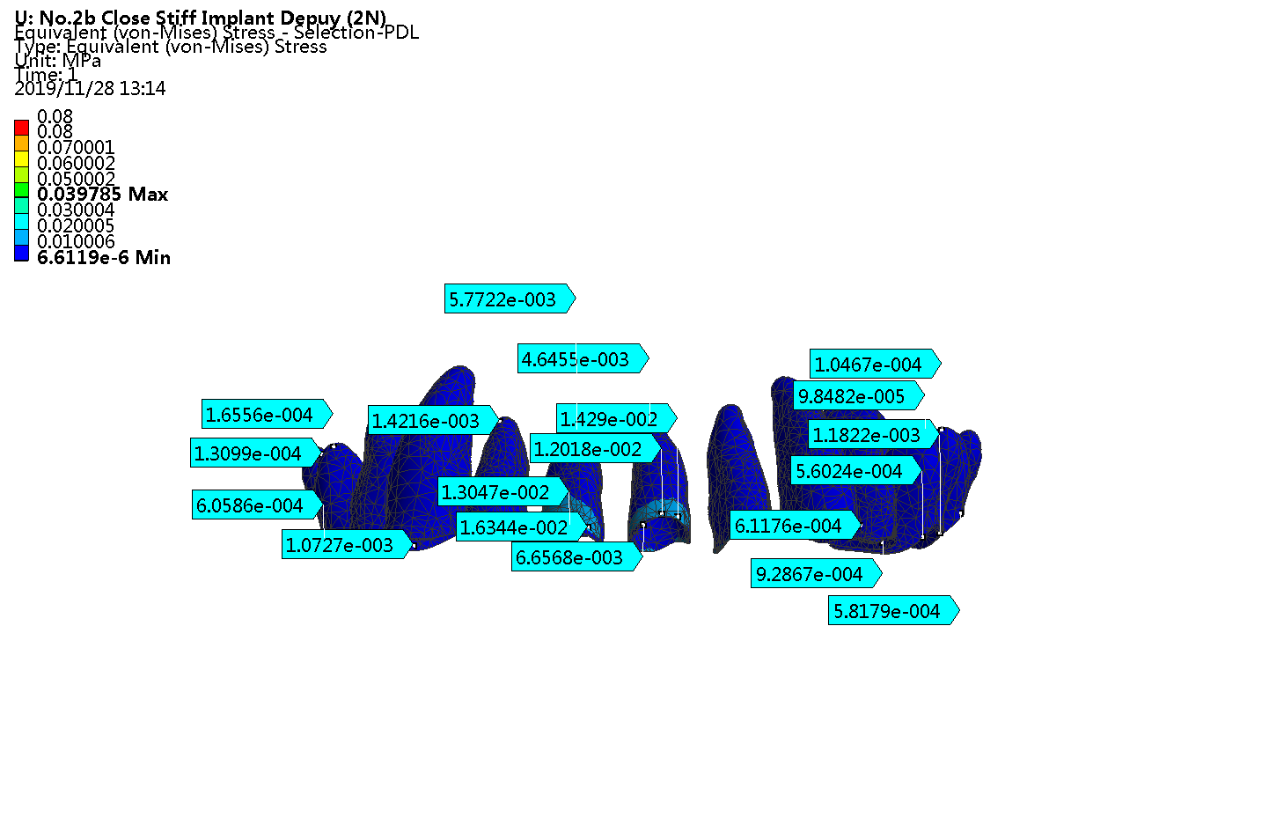

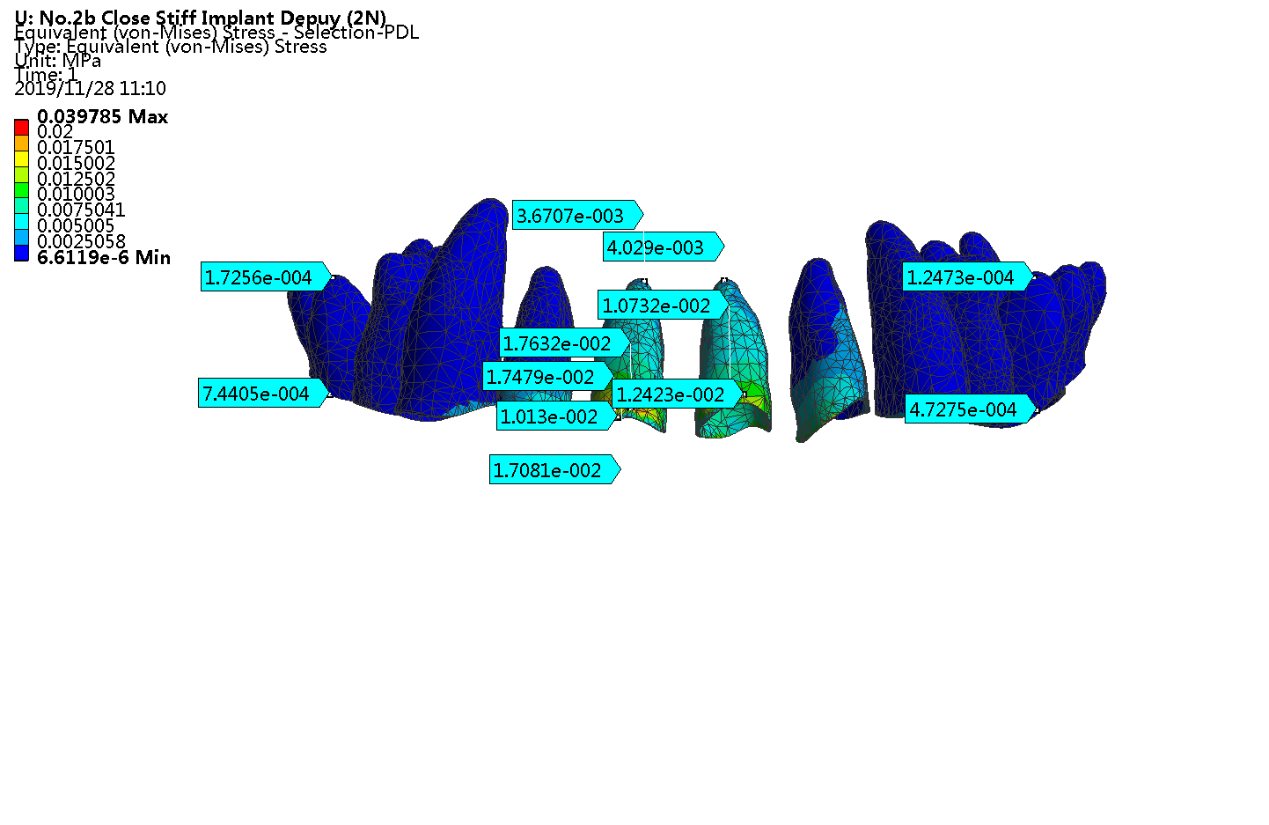

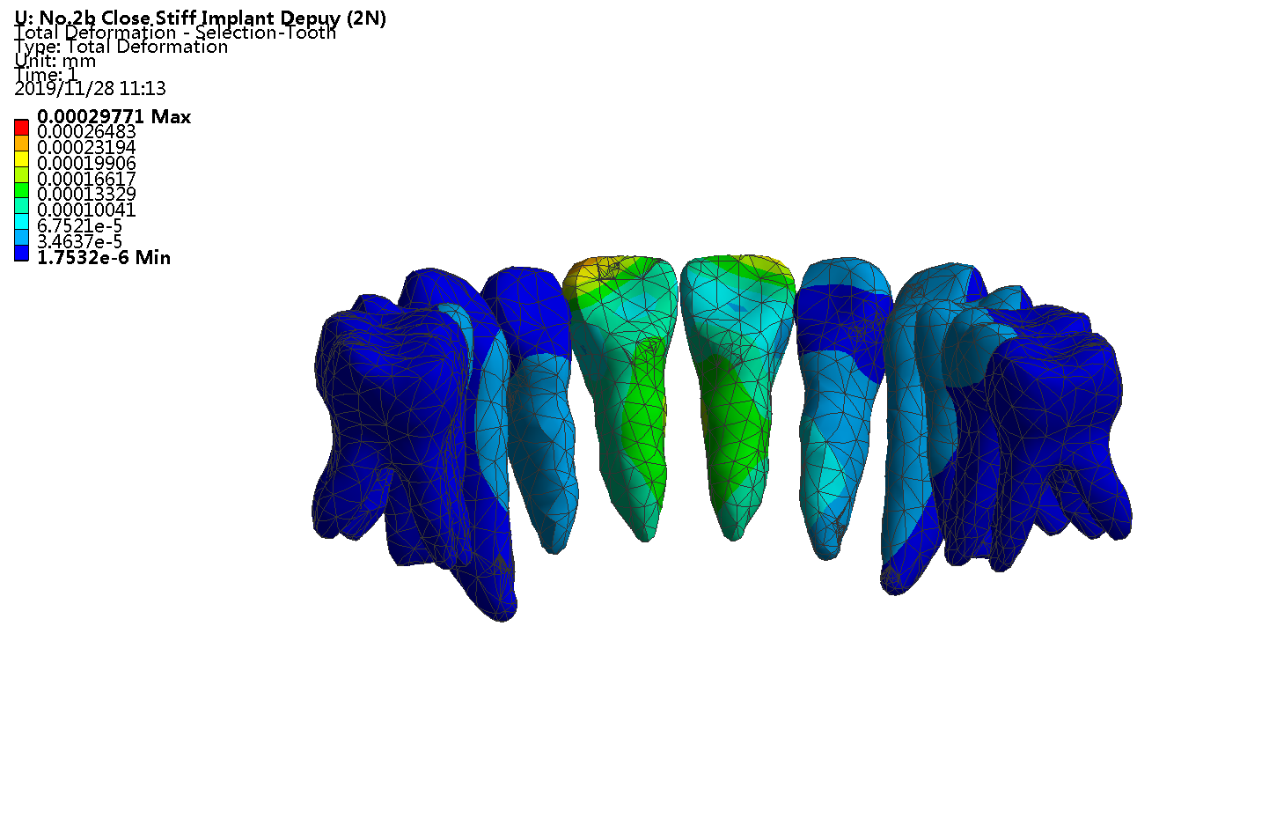

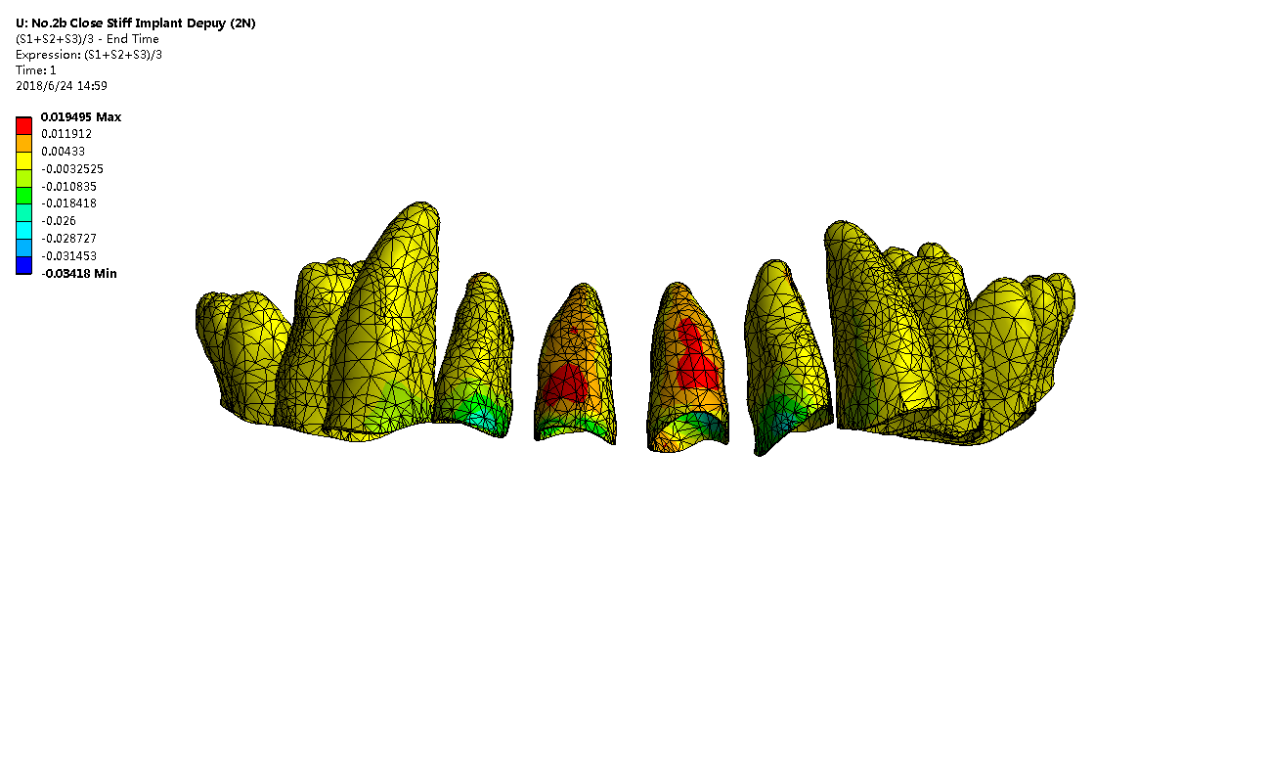

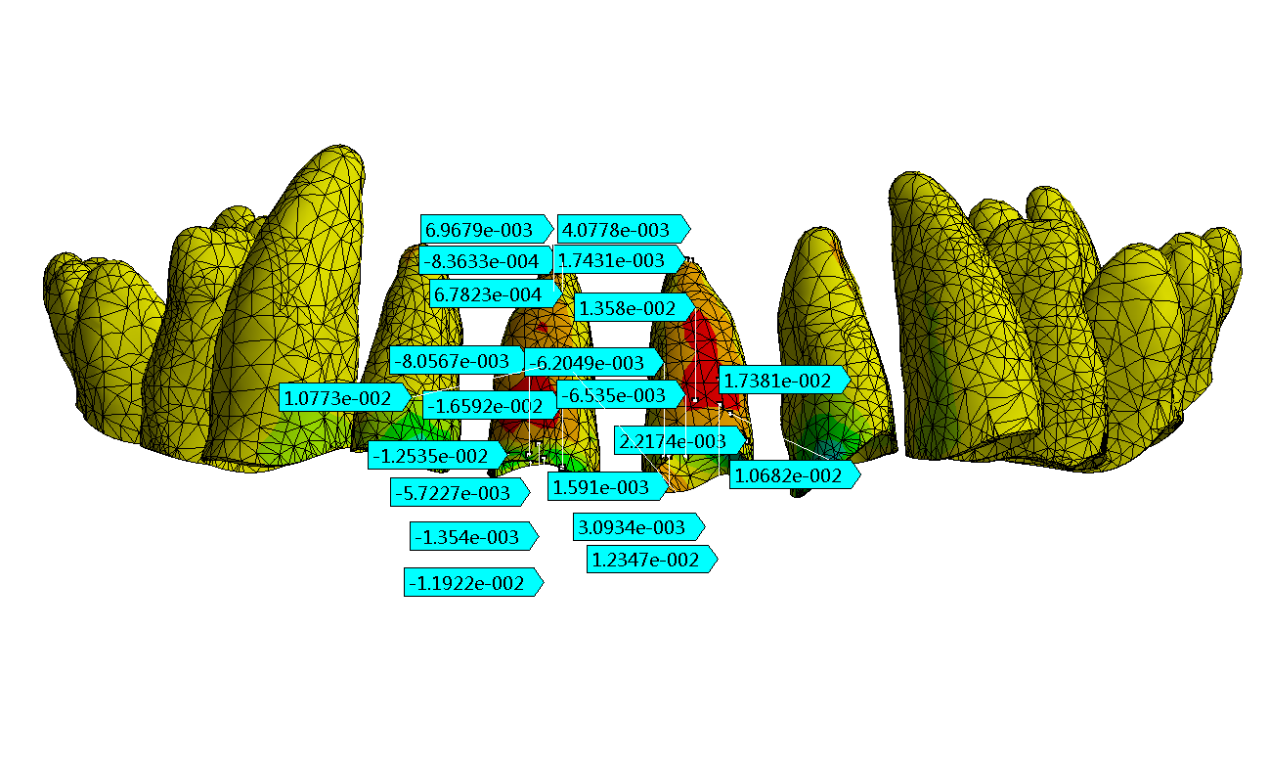

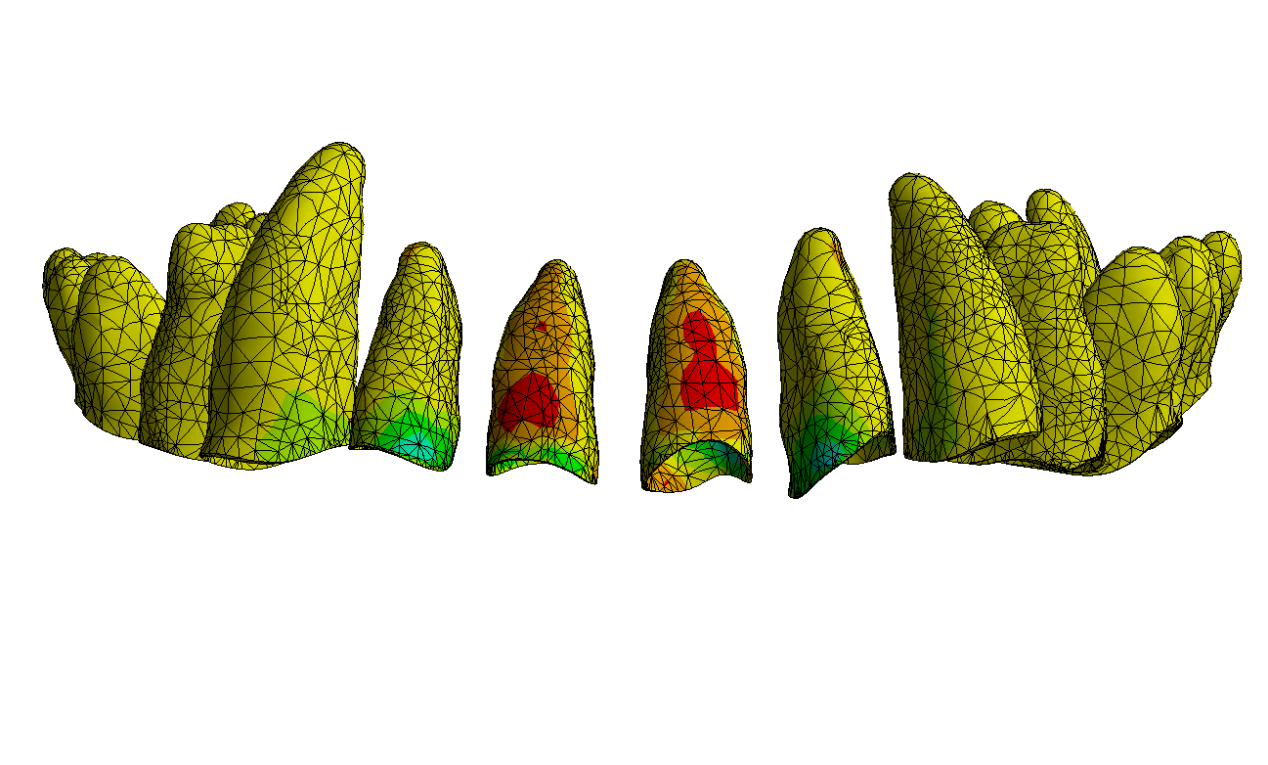

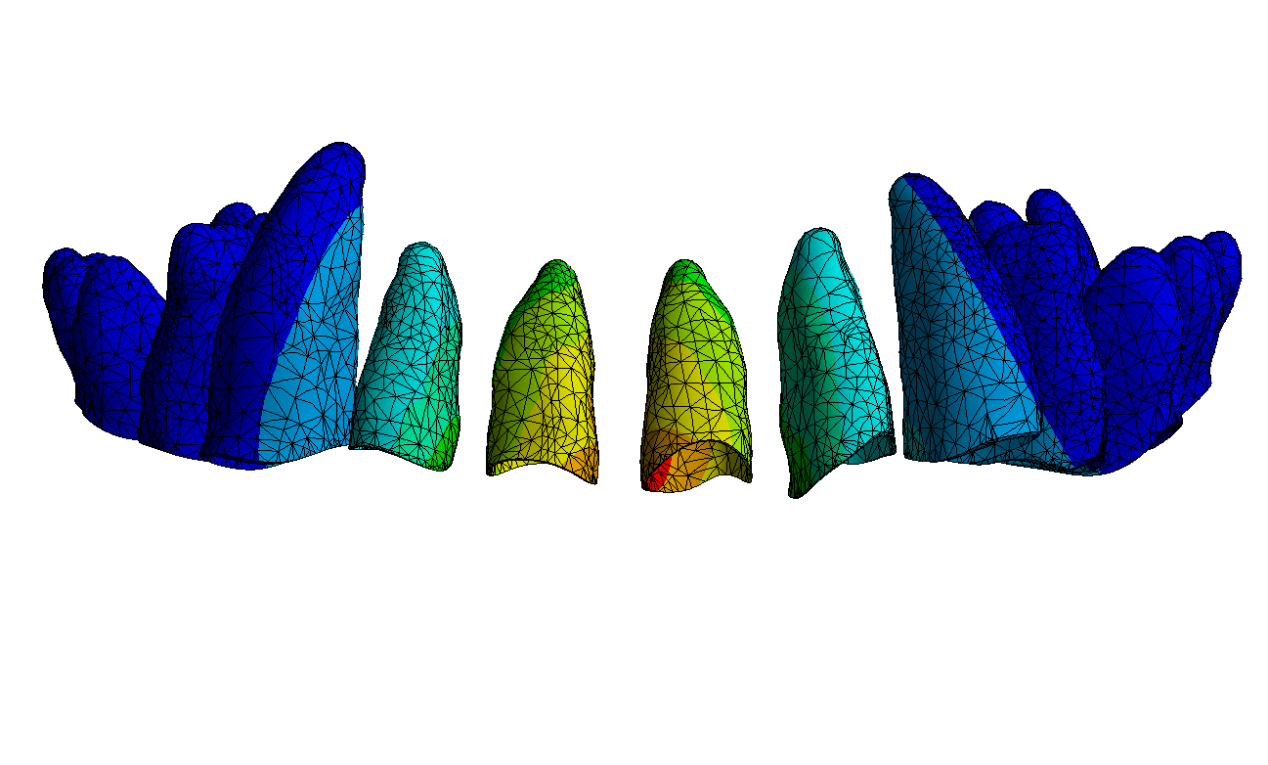


**Supplementary Figs S8: 2d group**

**3 group:**


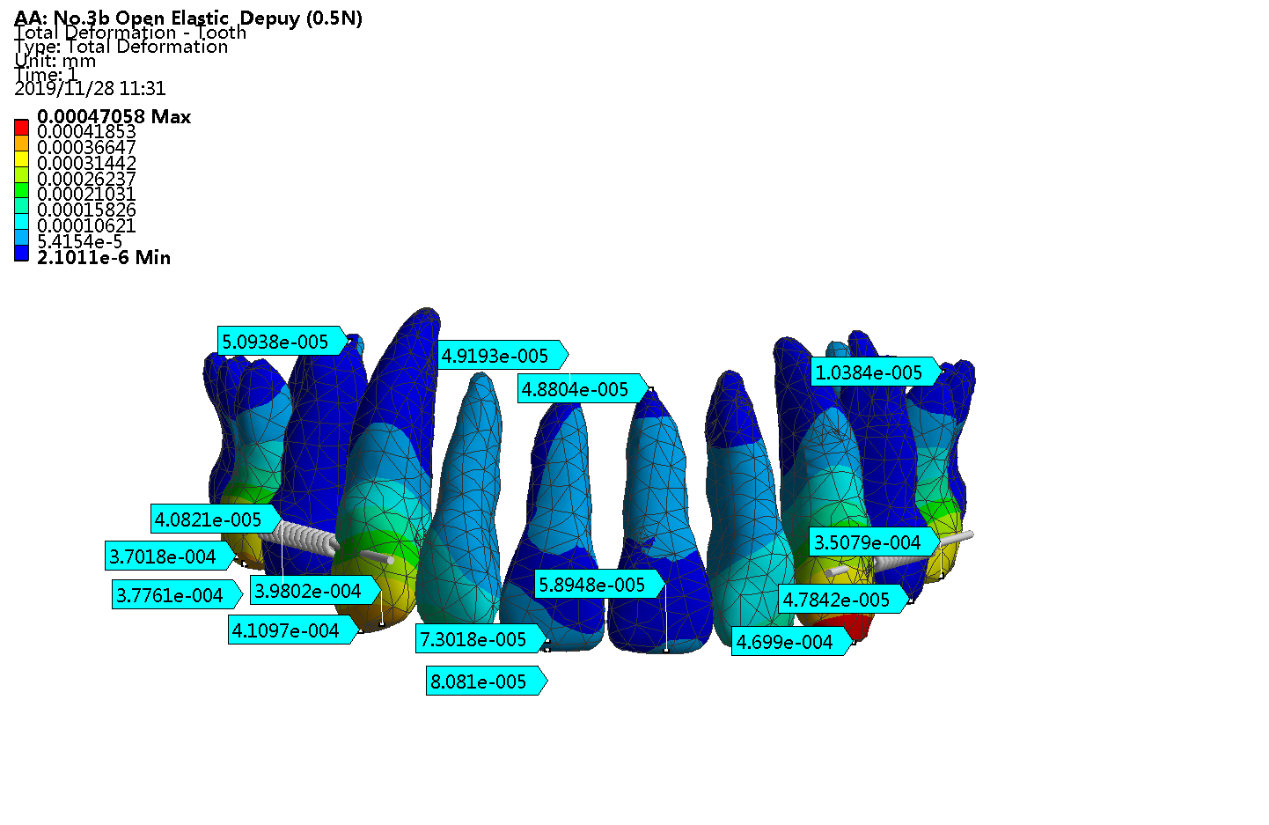

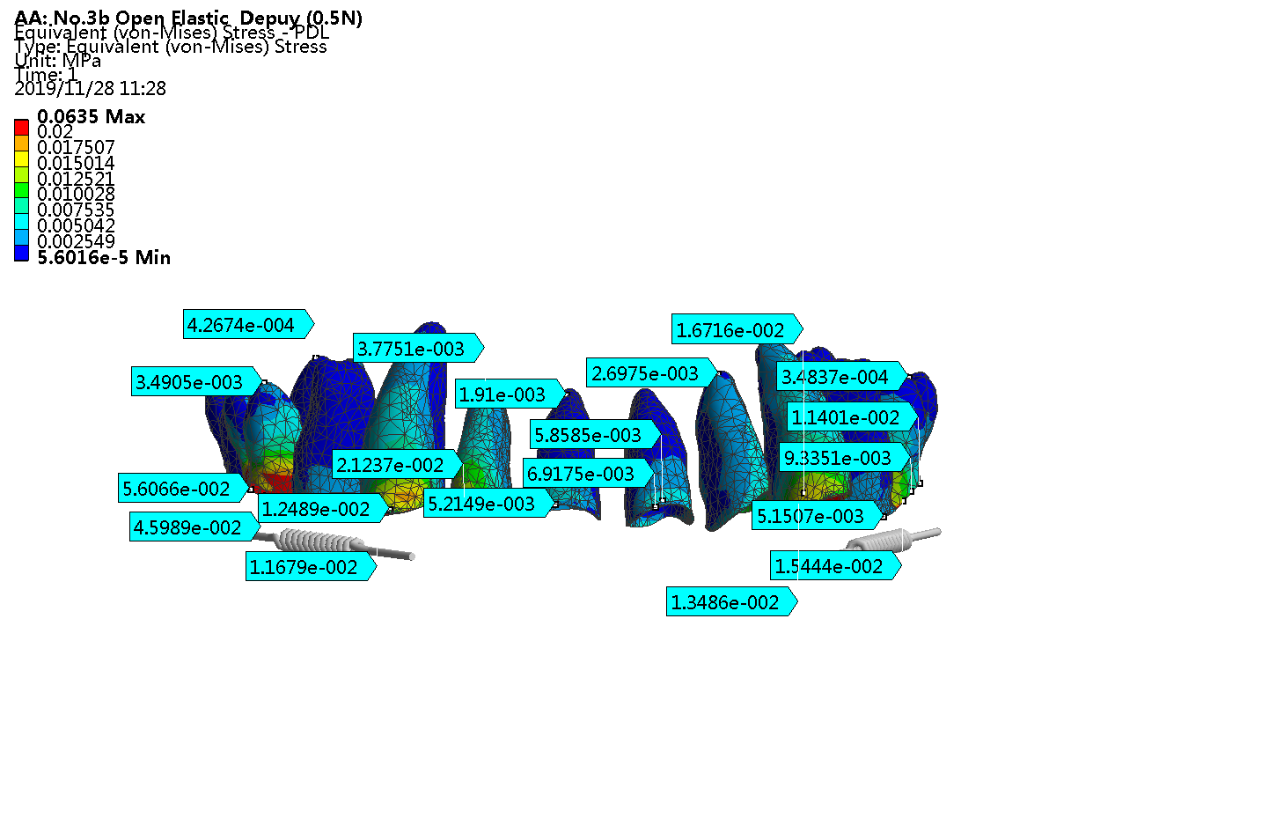

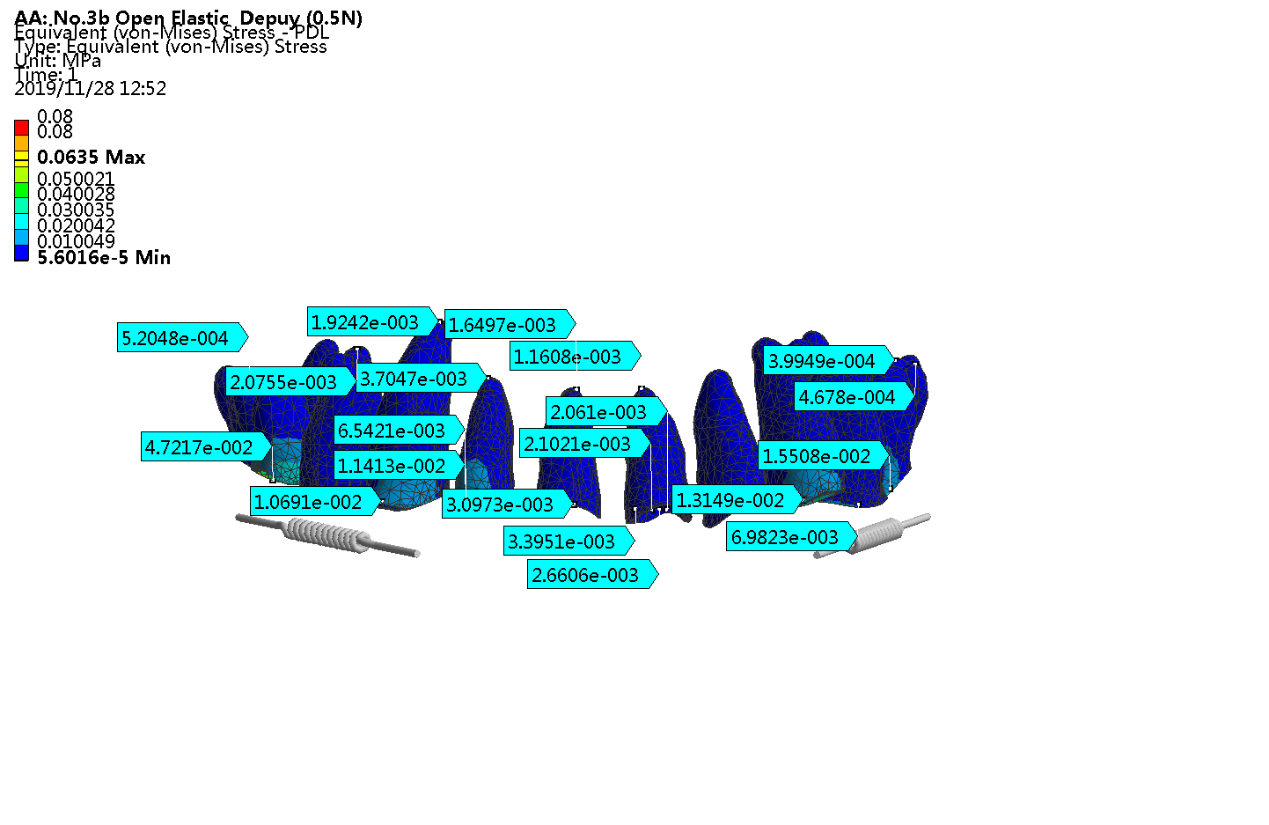

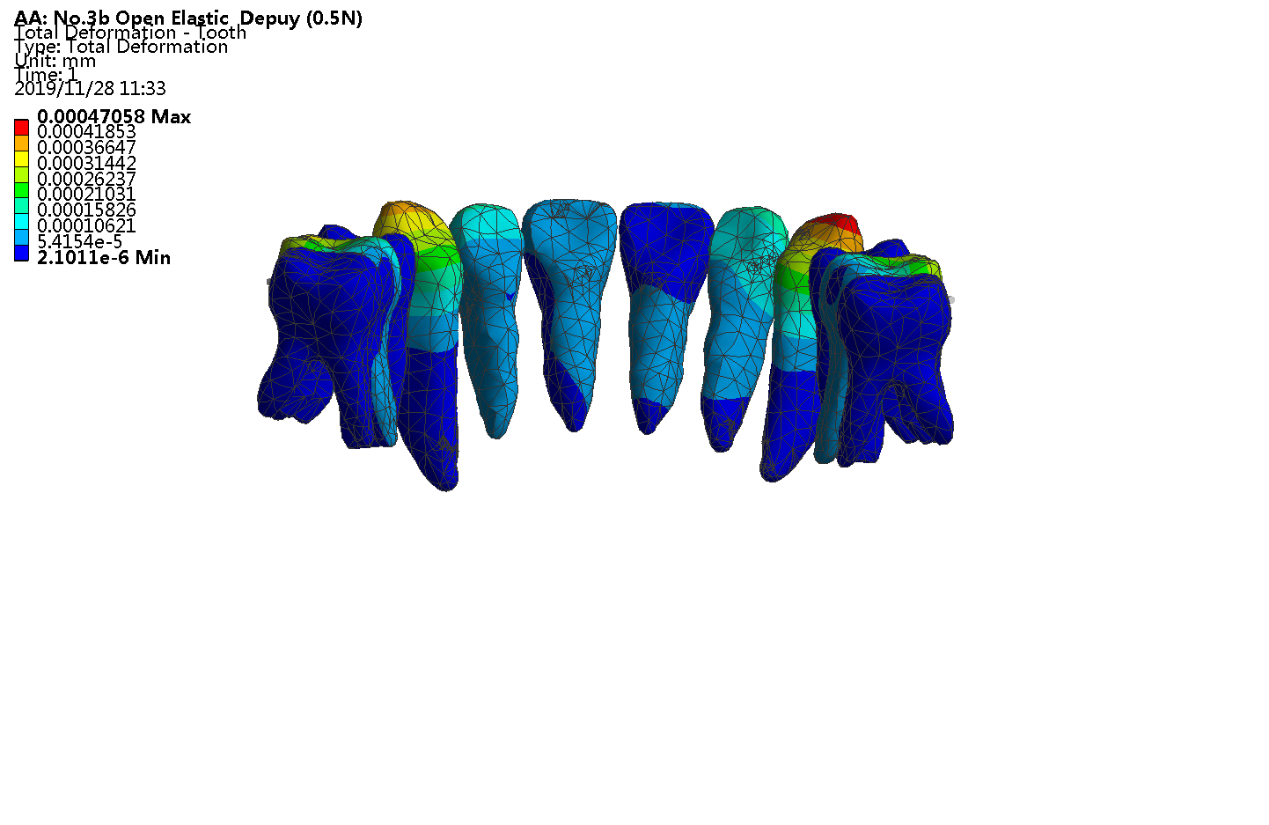

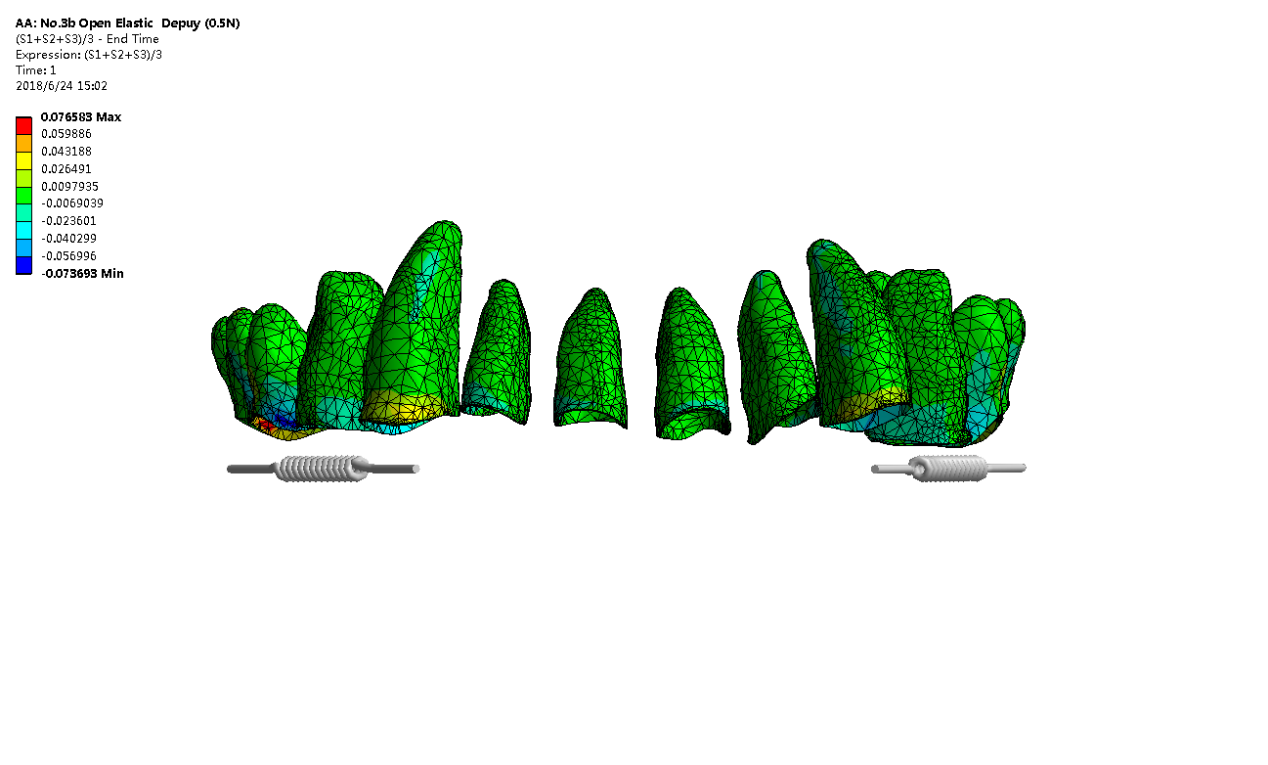

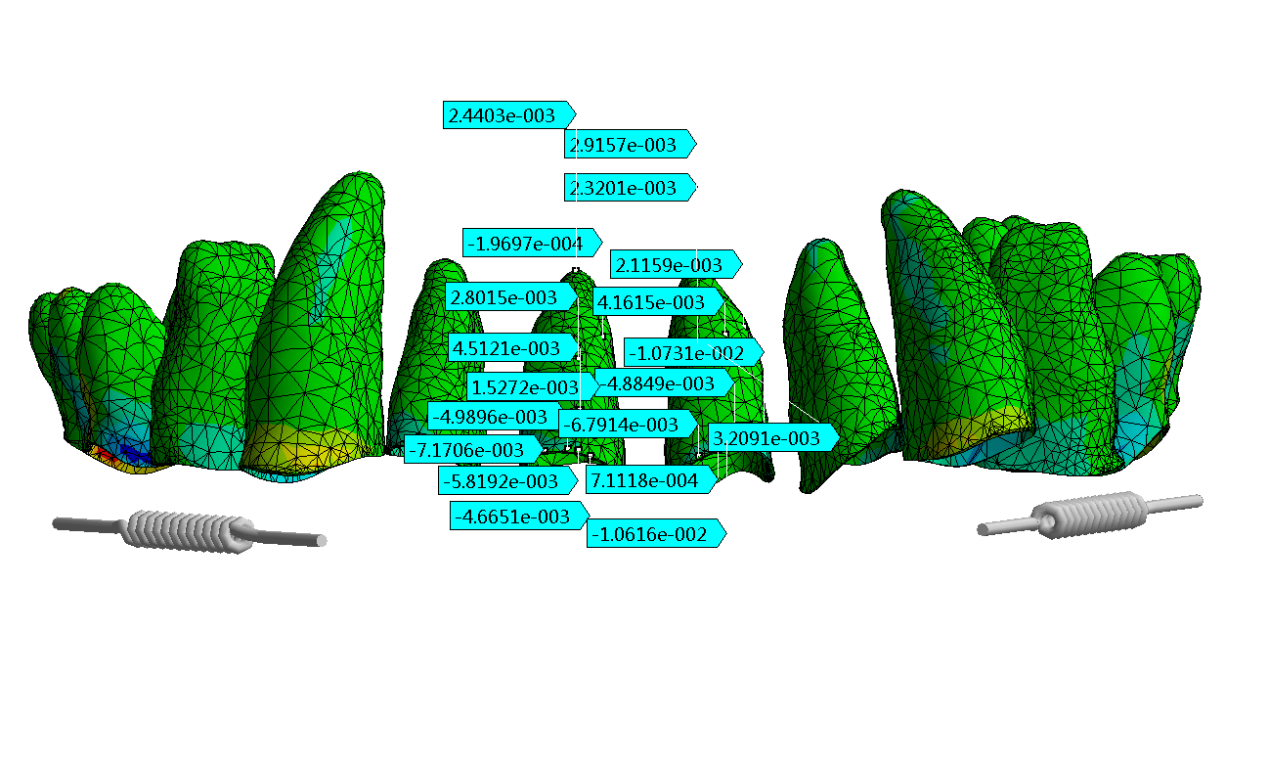

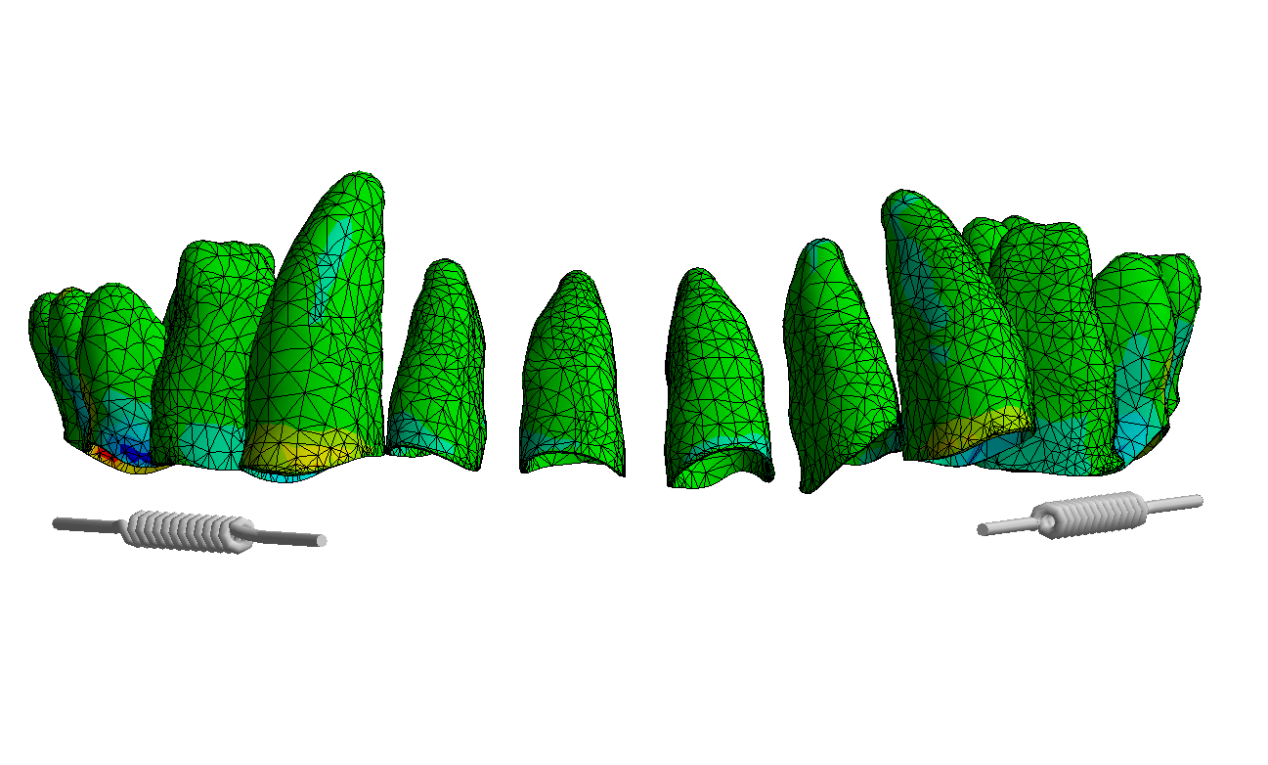
 **Supplementary Figs S9: 3a group**


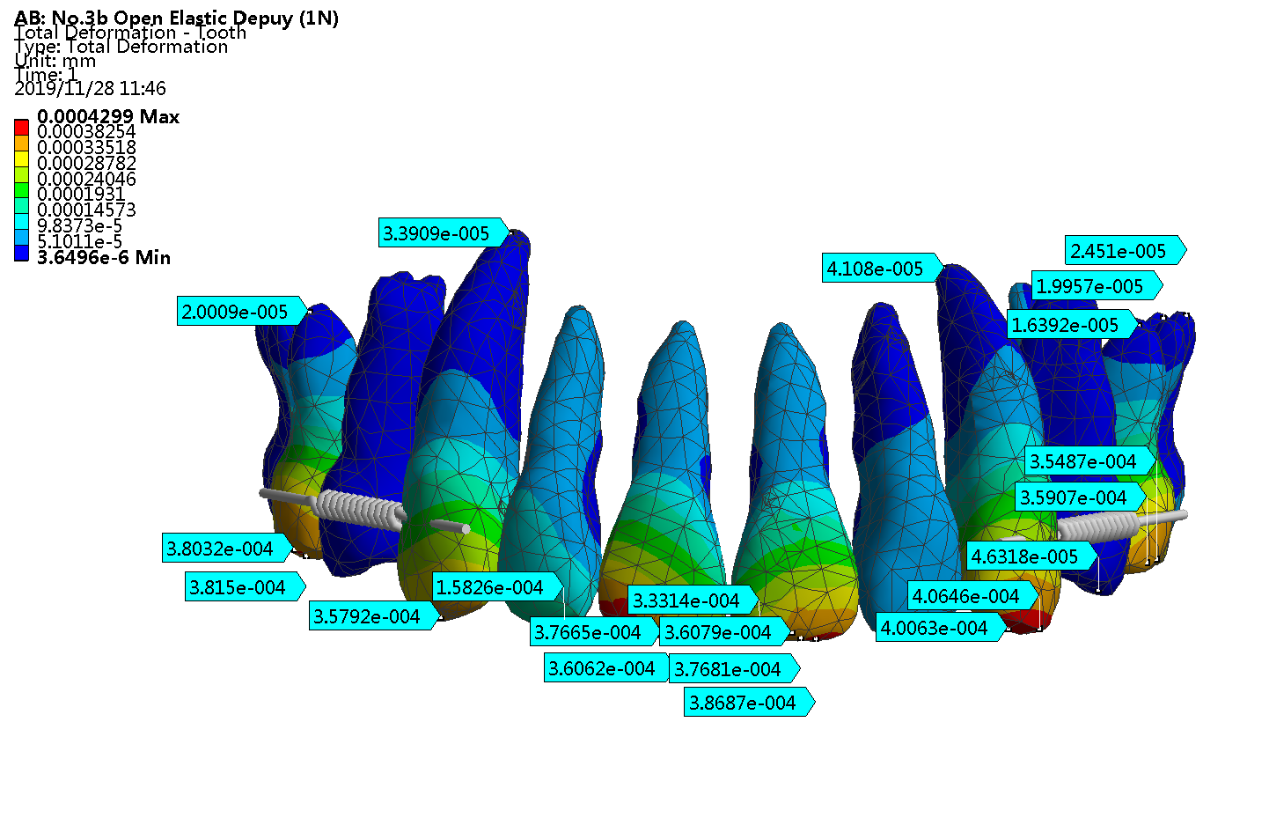

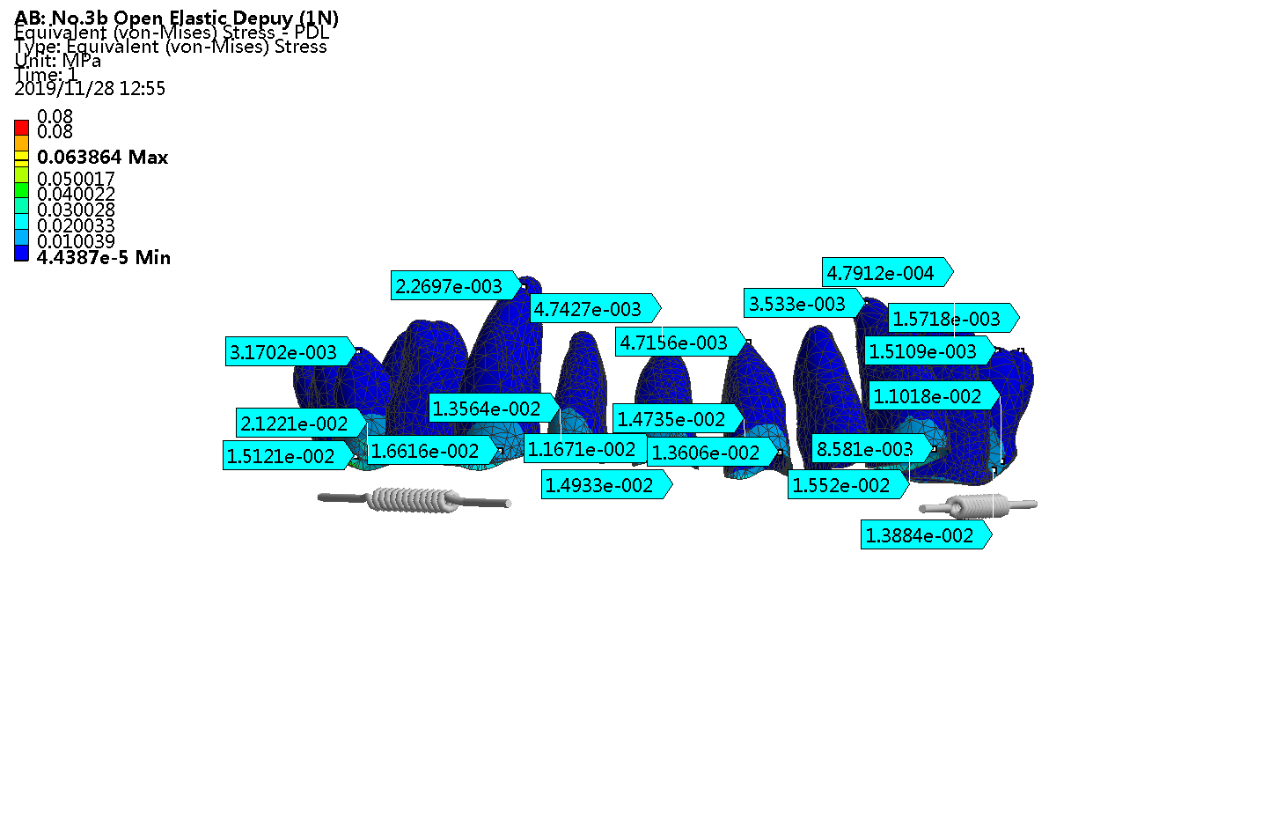

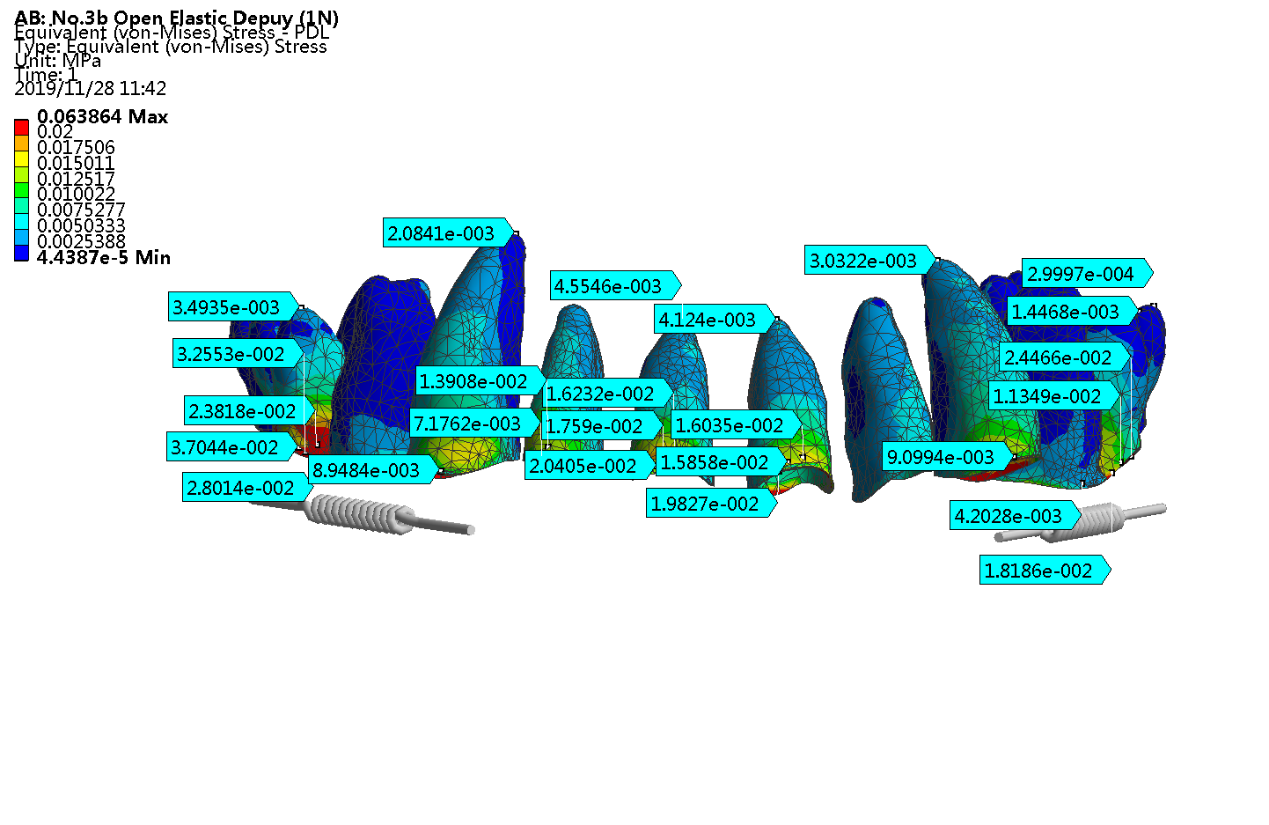

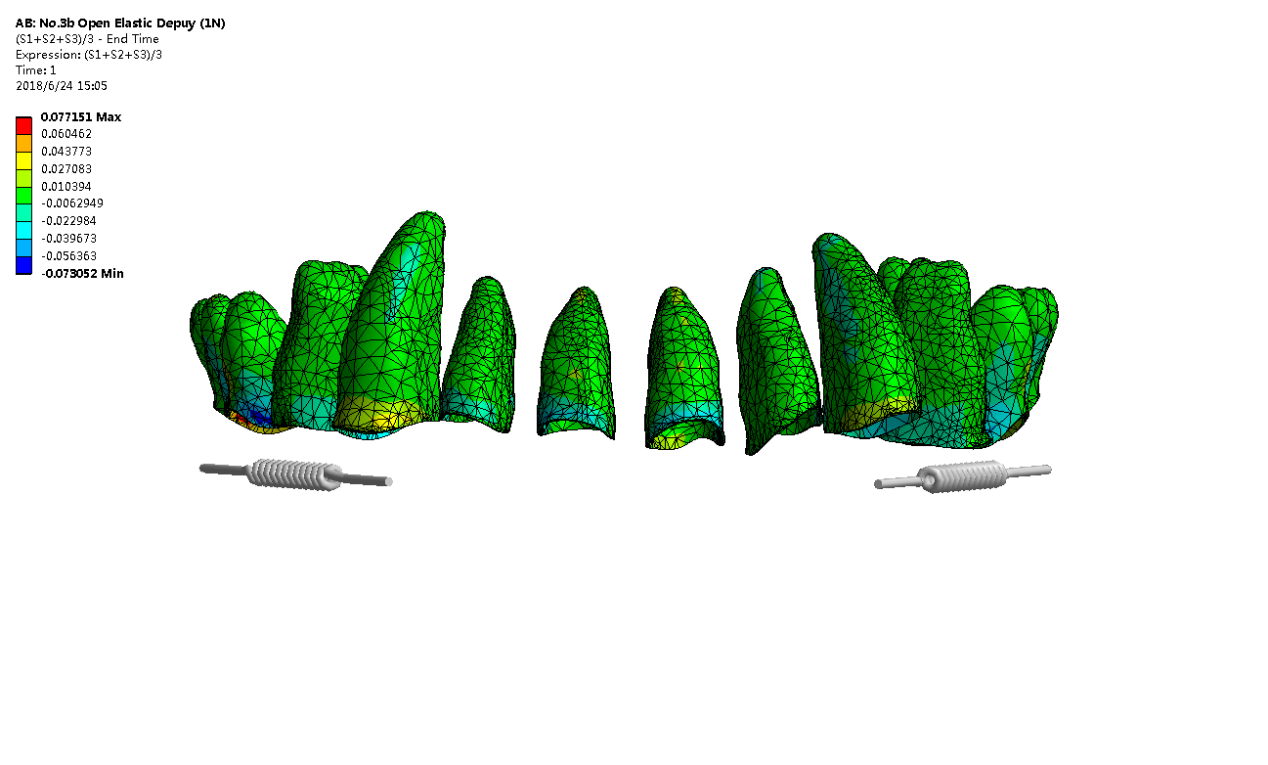

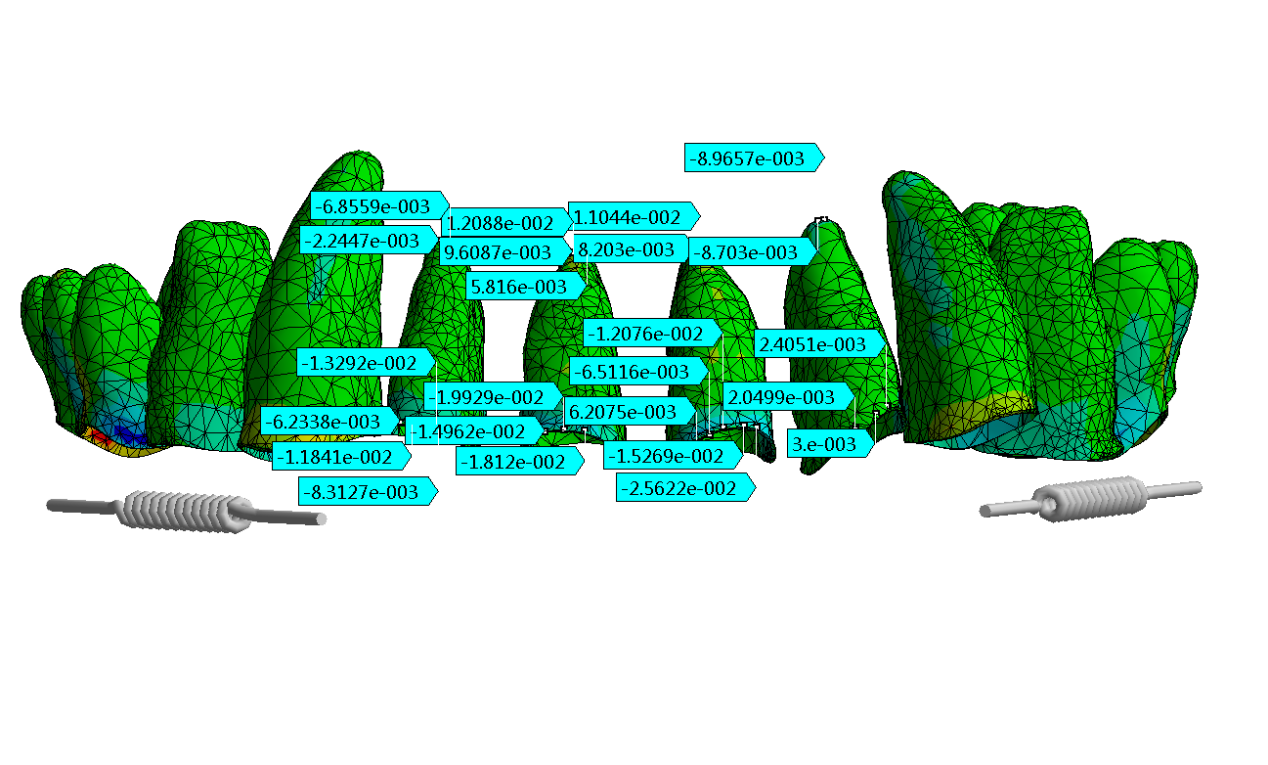

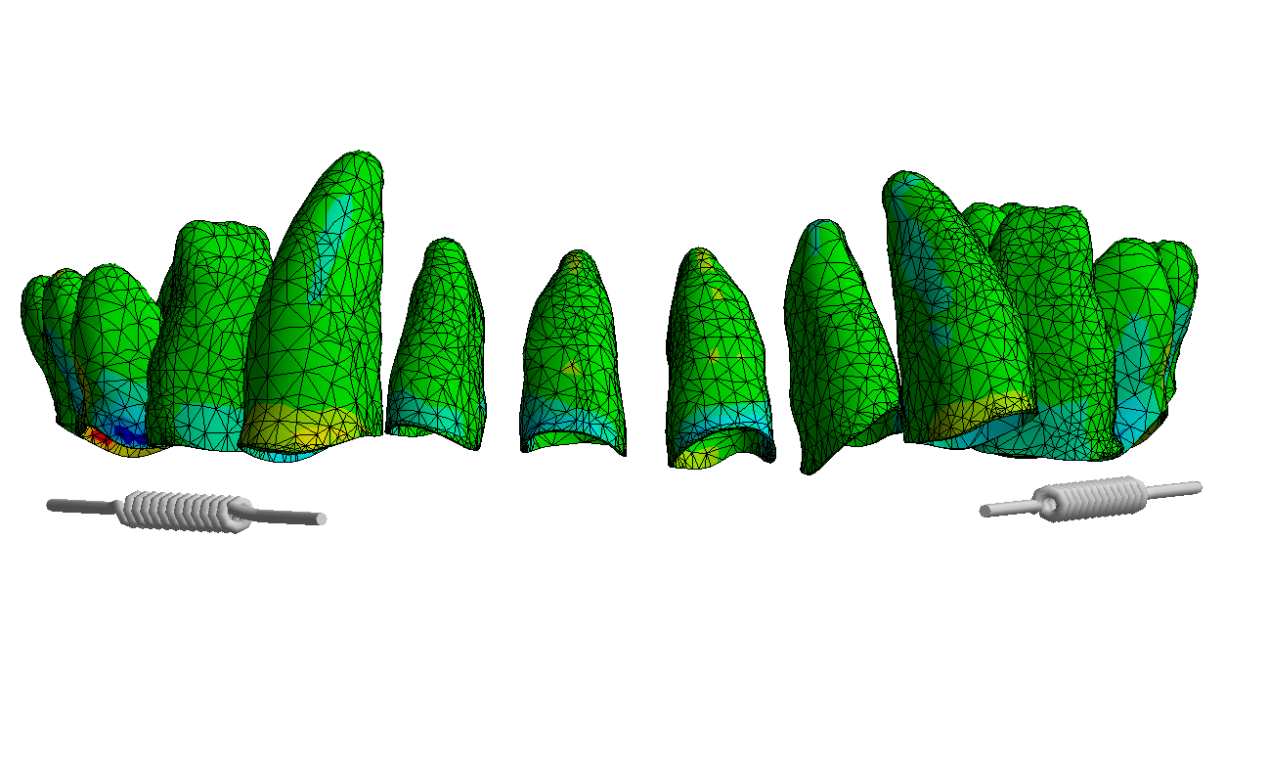


**Supplementary Figs S10: 3b group**


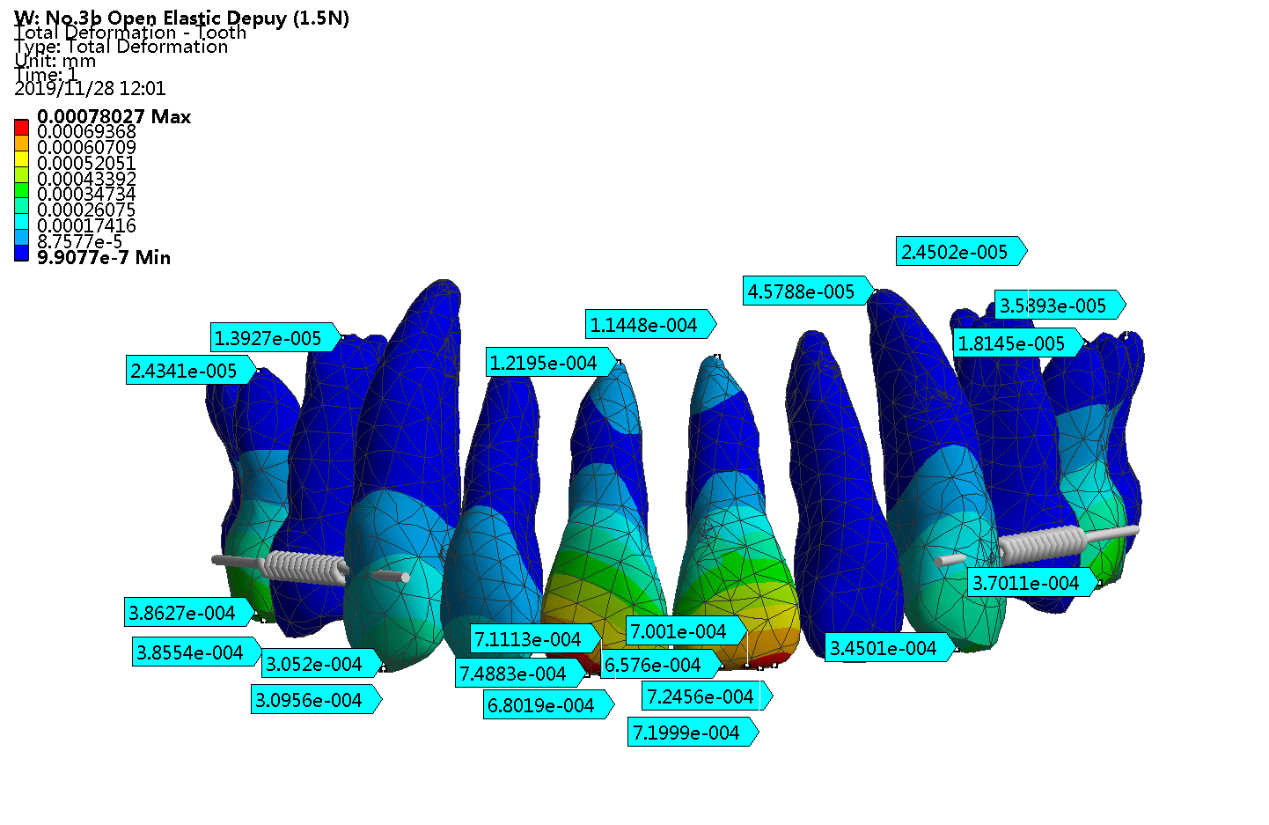

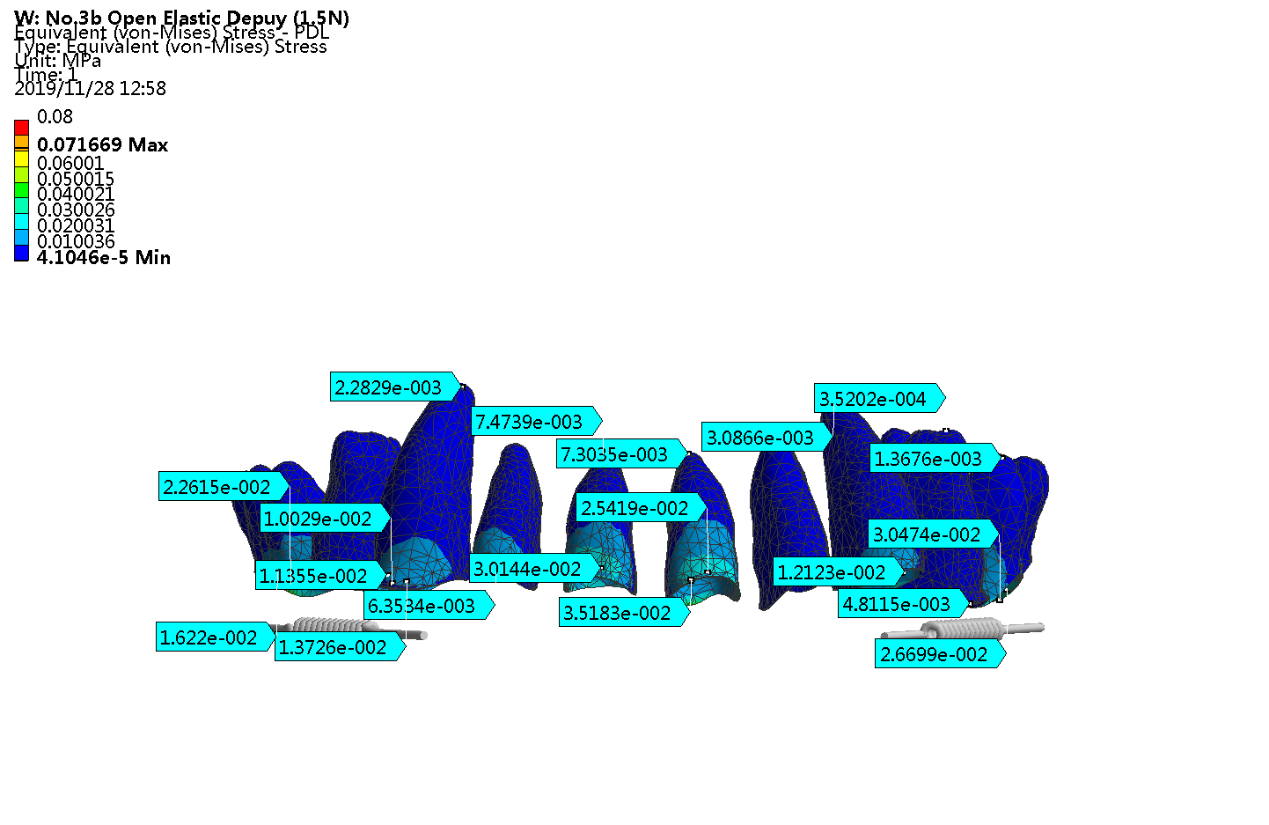

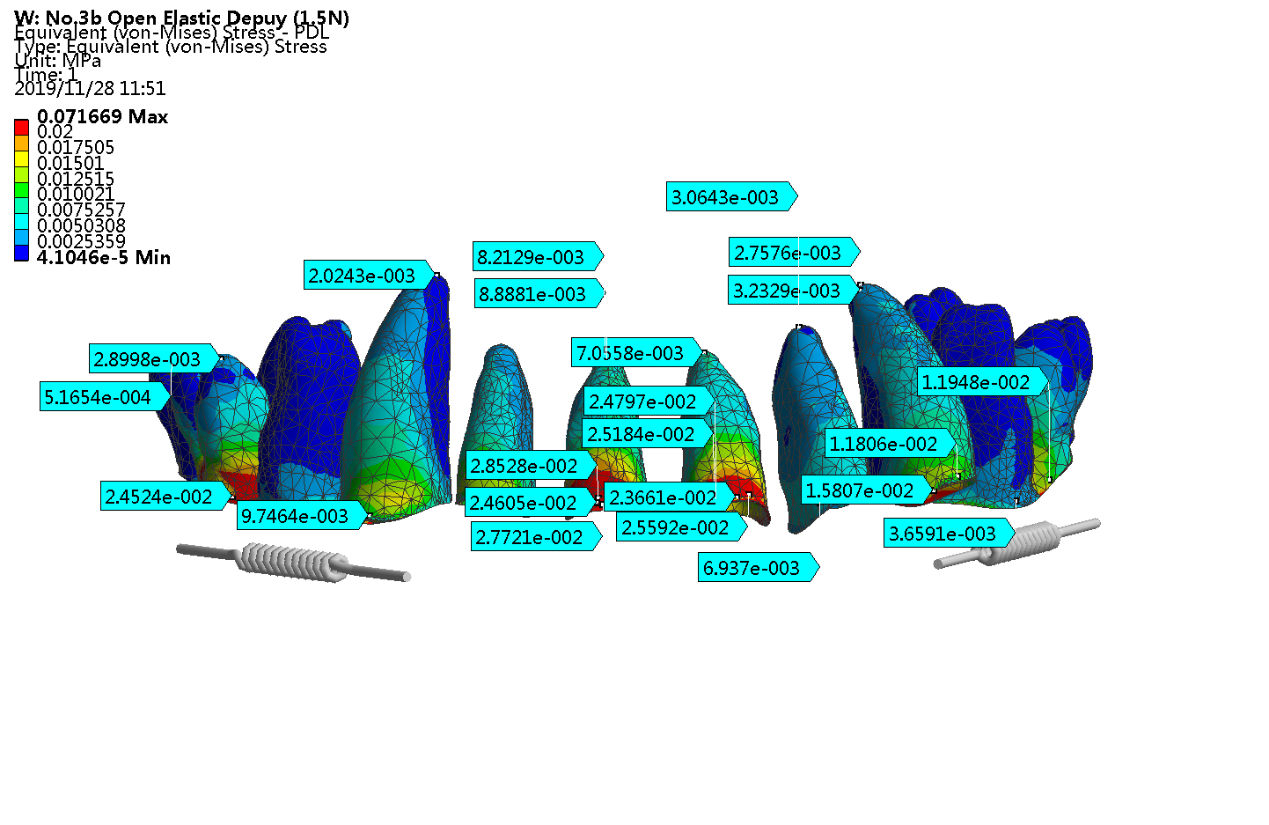

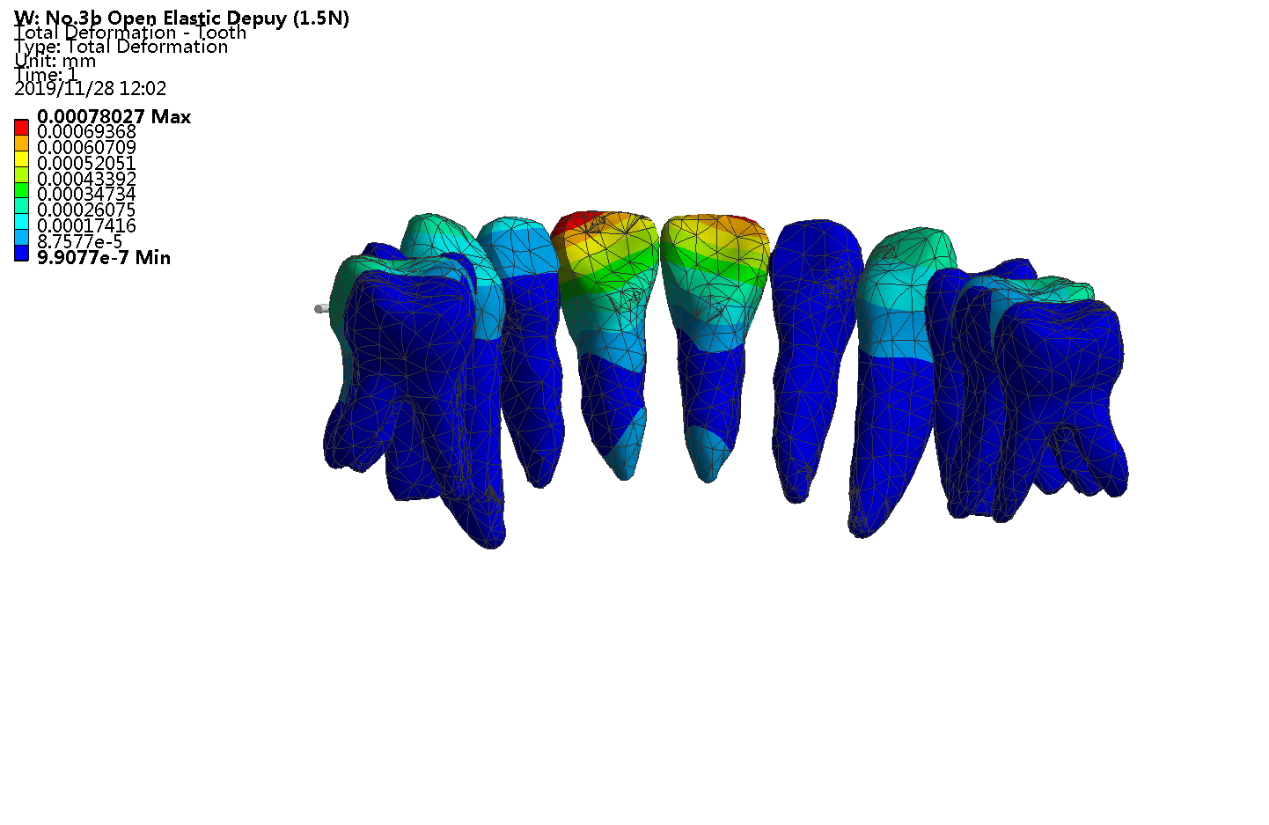

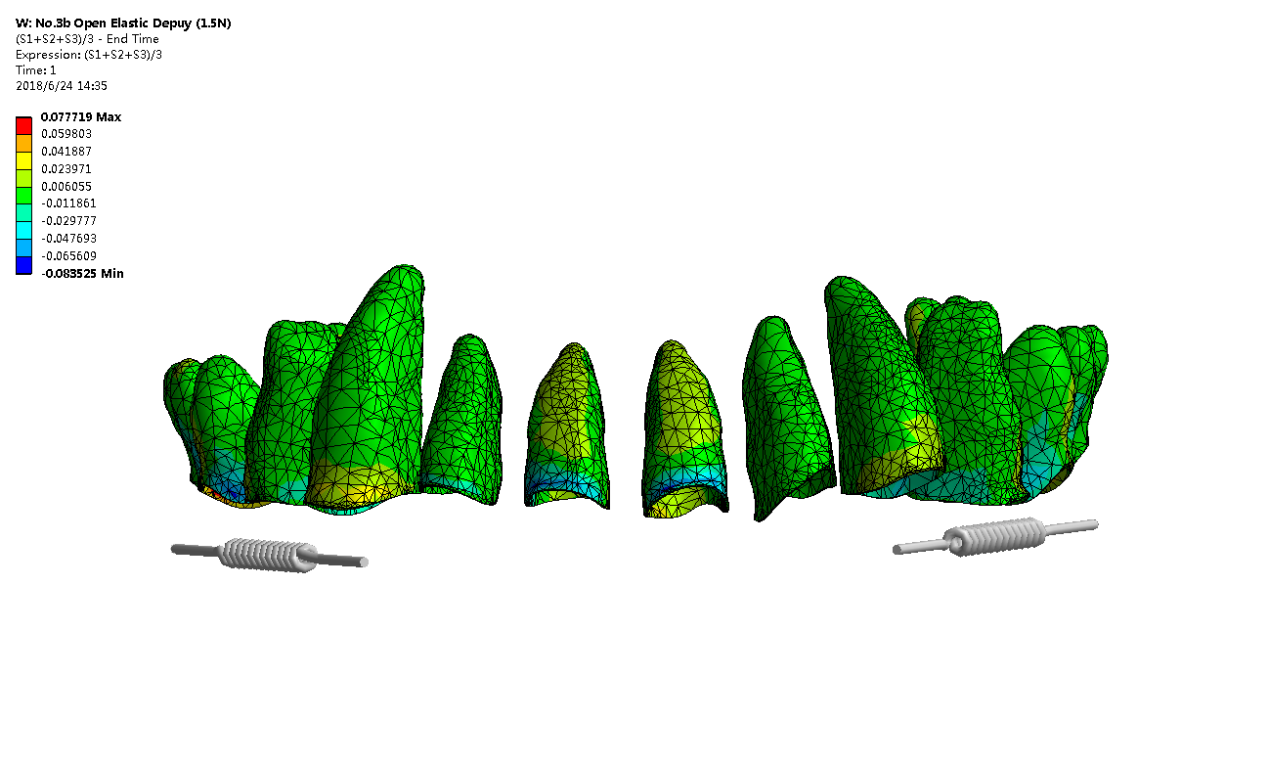

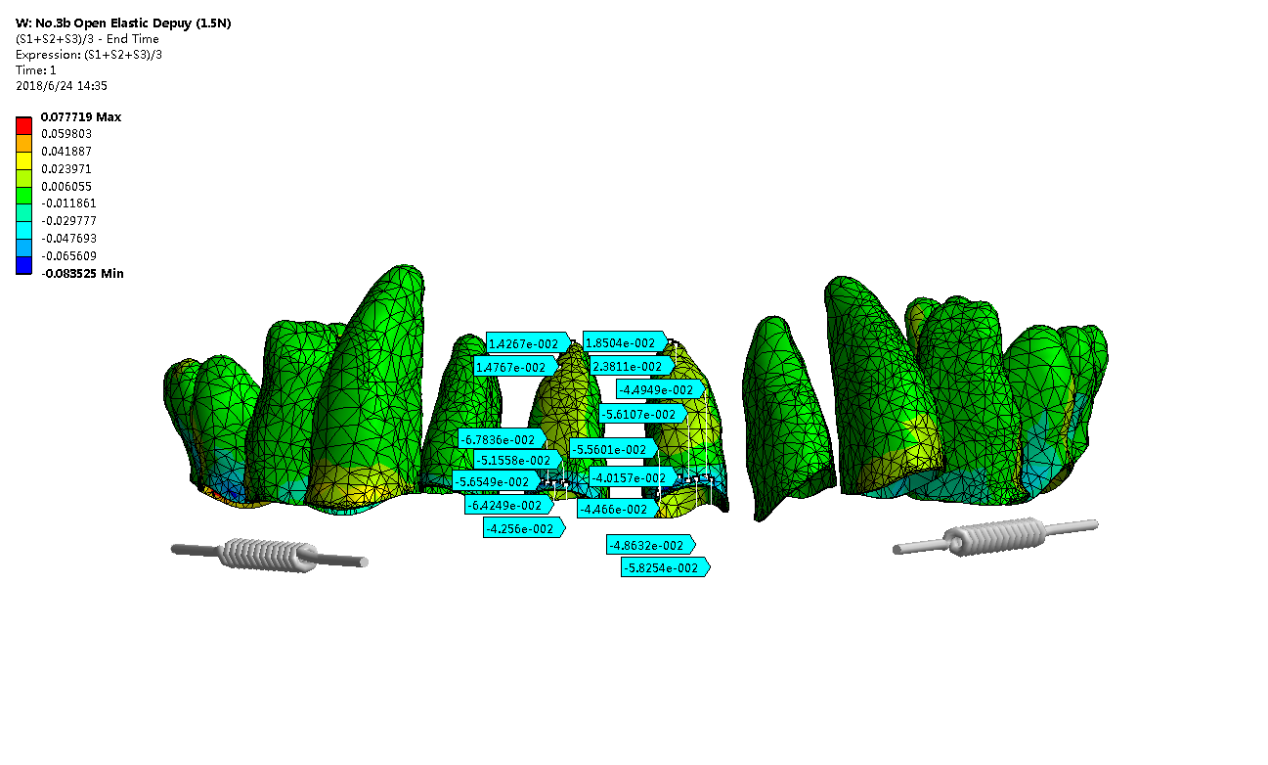

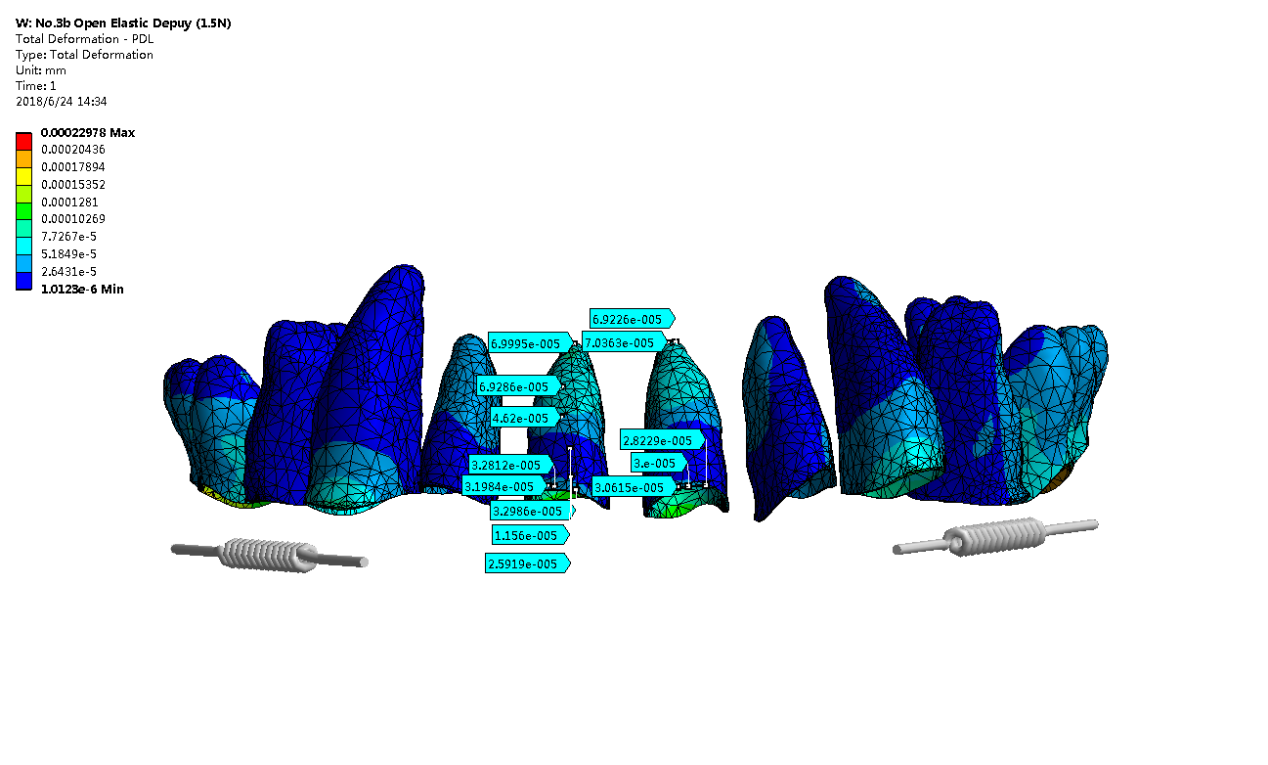

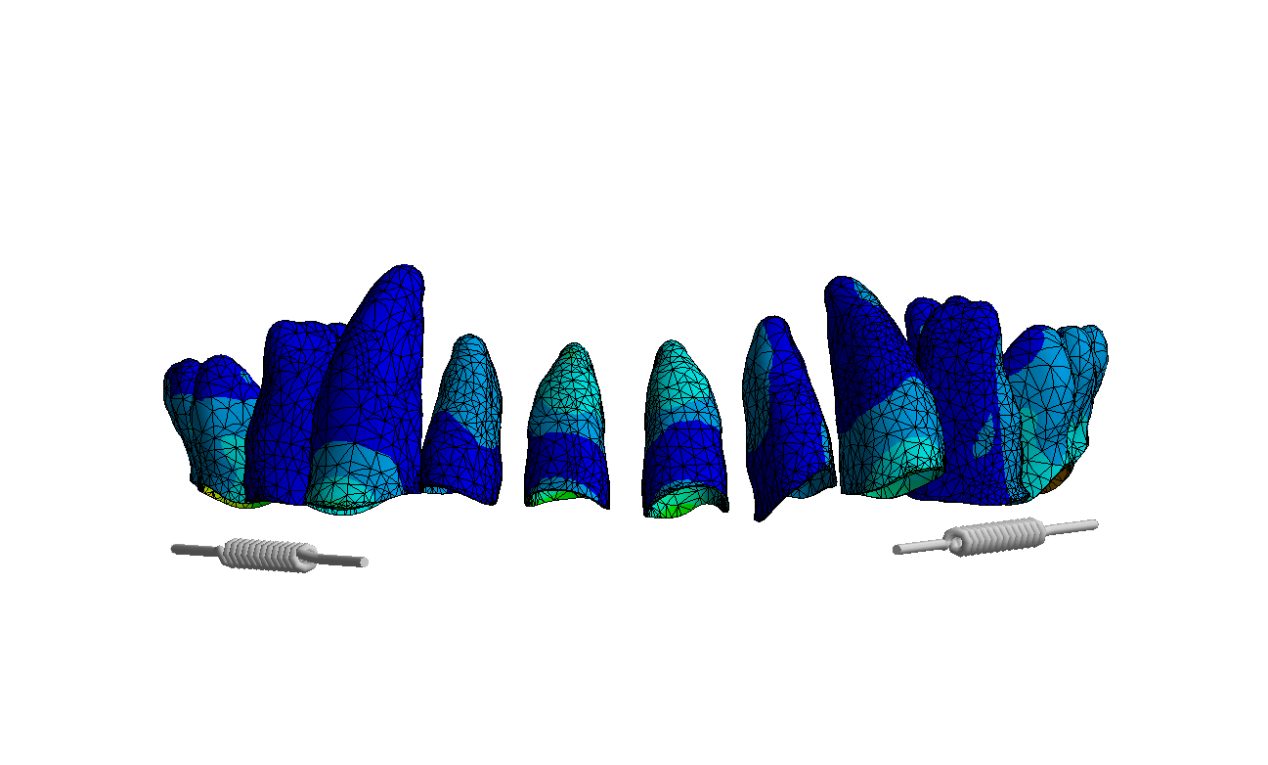

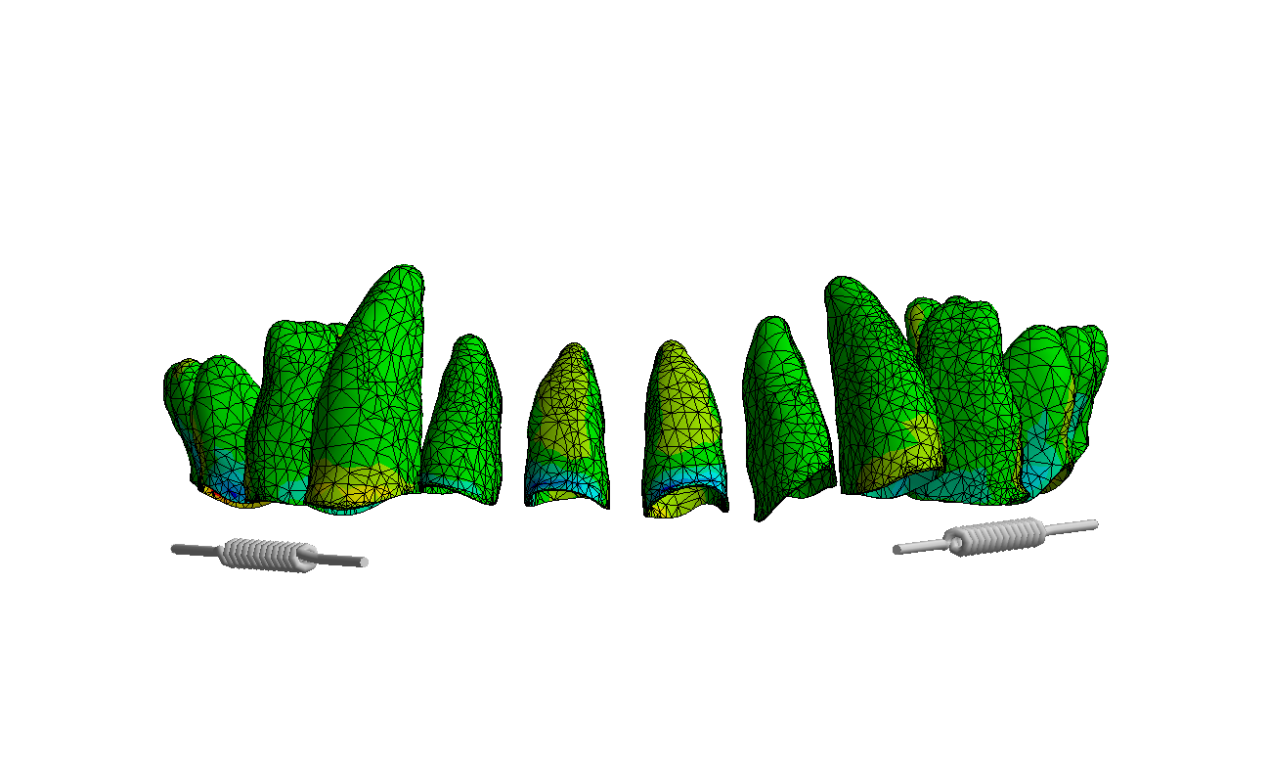


**Supplementary Figs S11: 3c group**


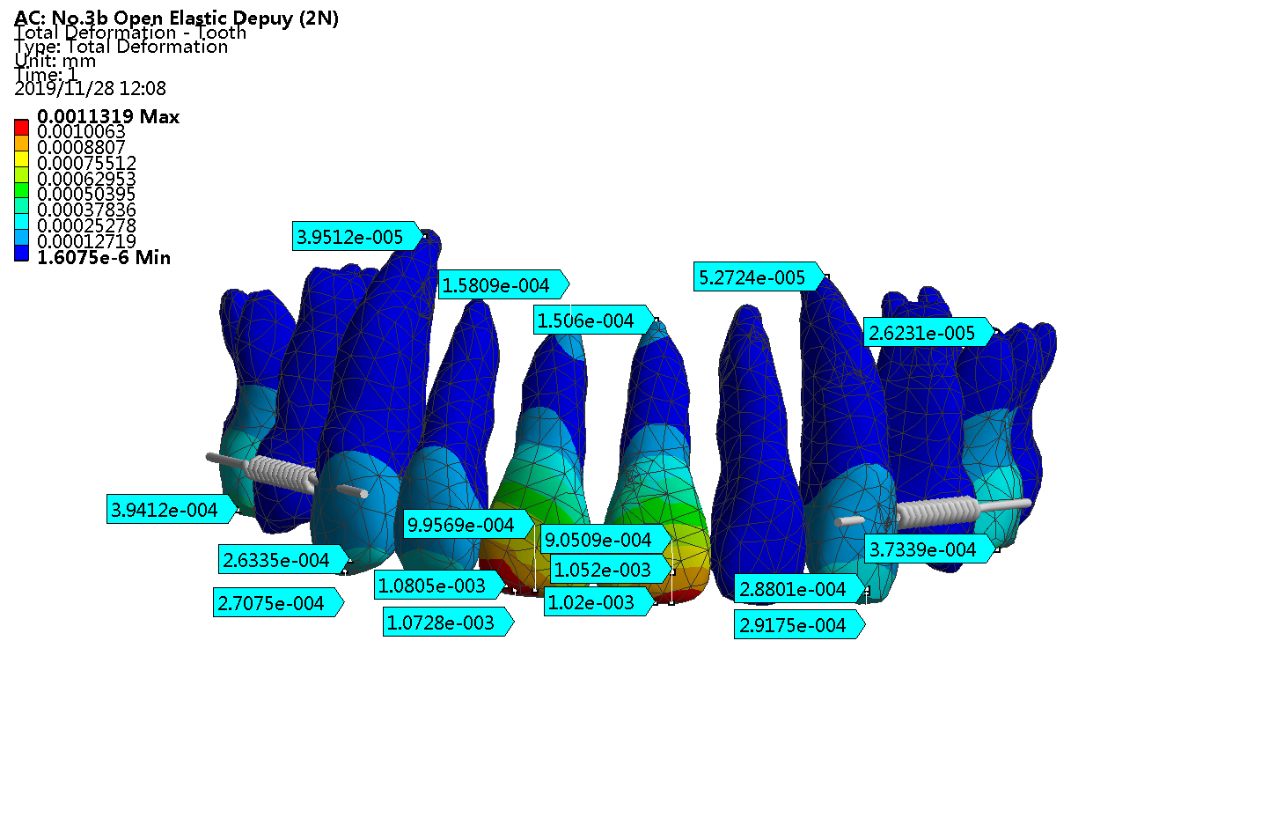

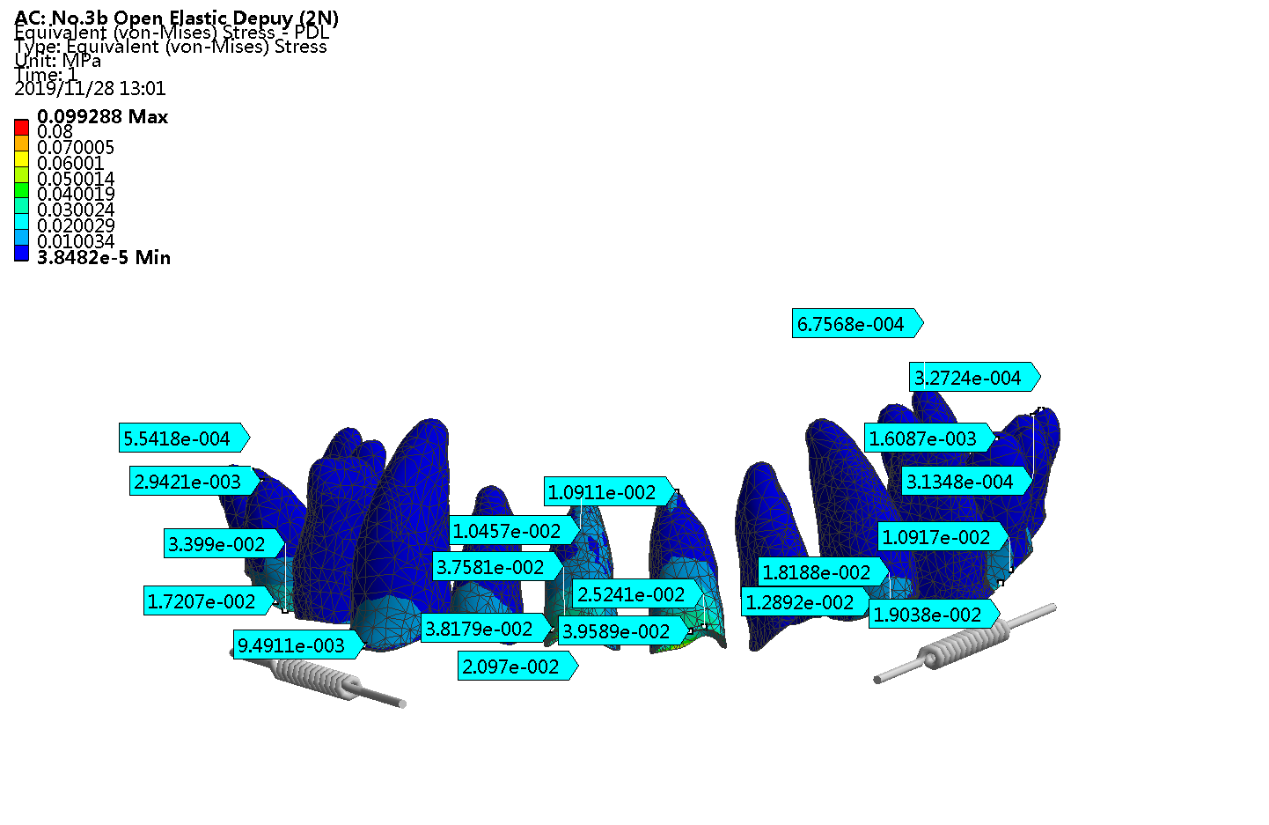

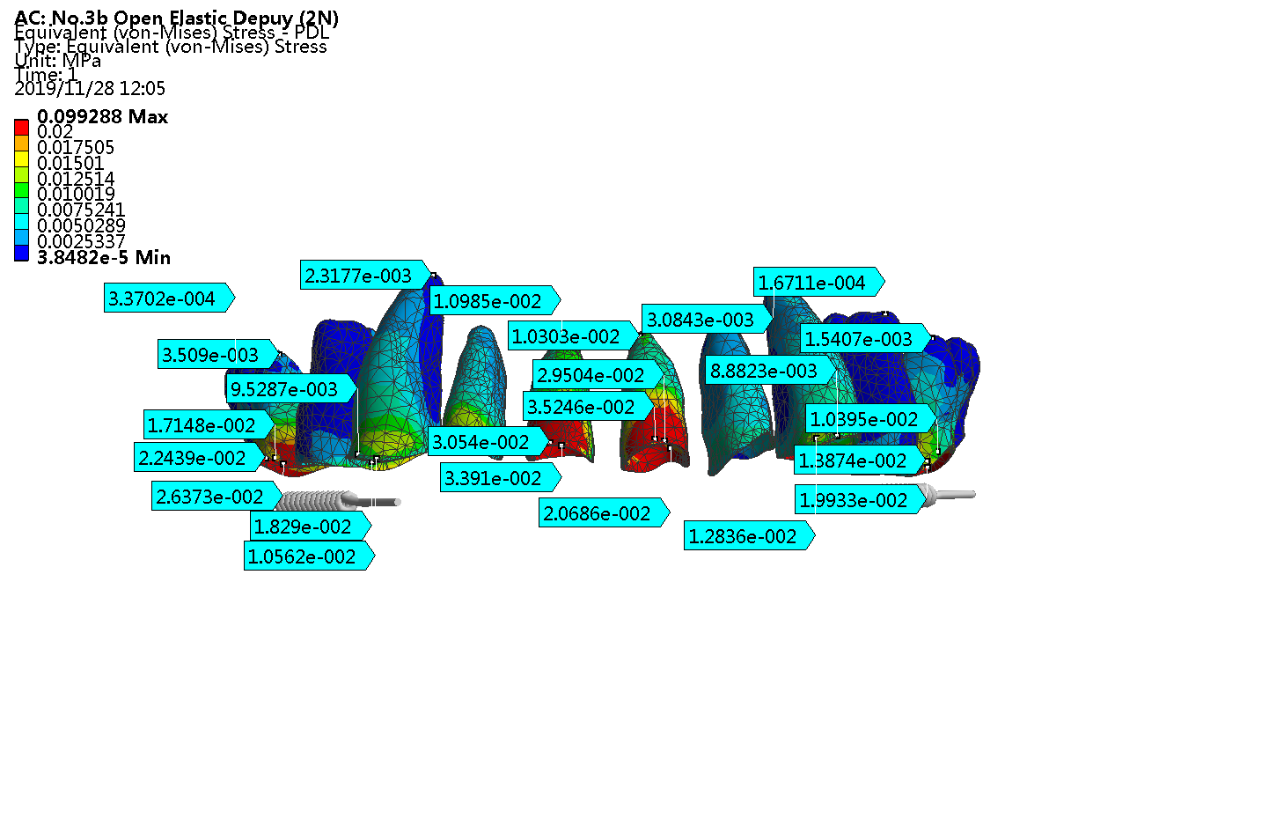

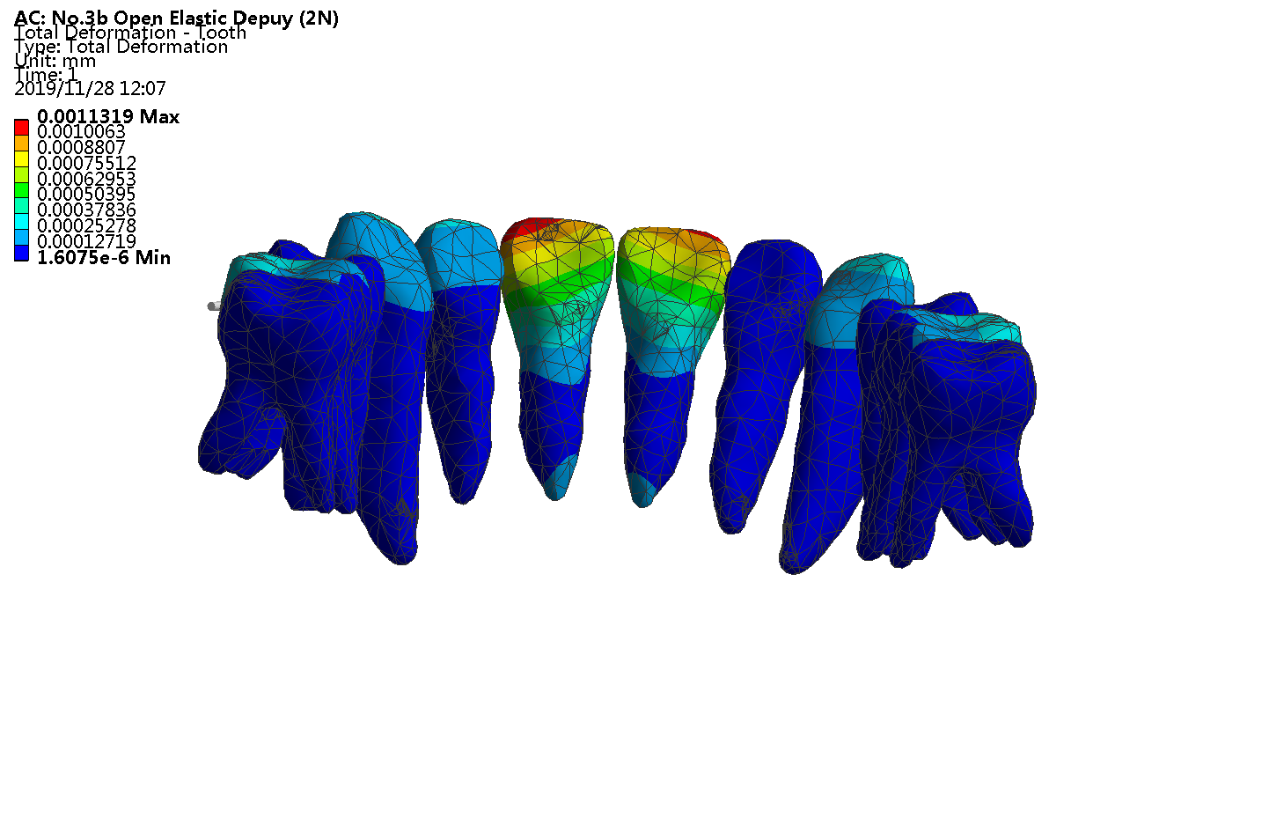

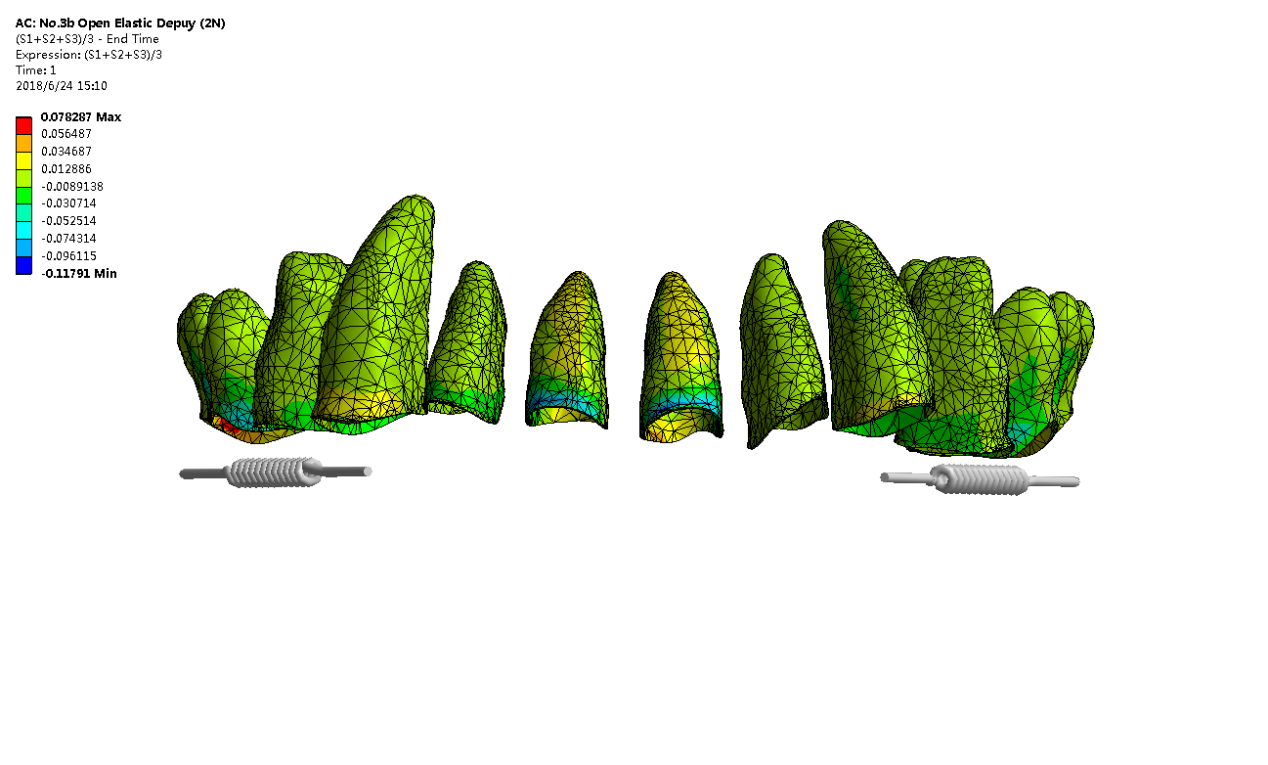

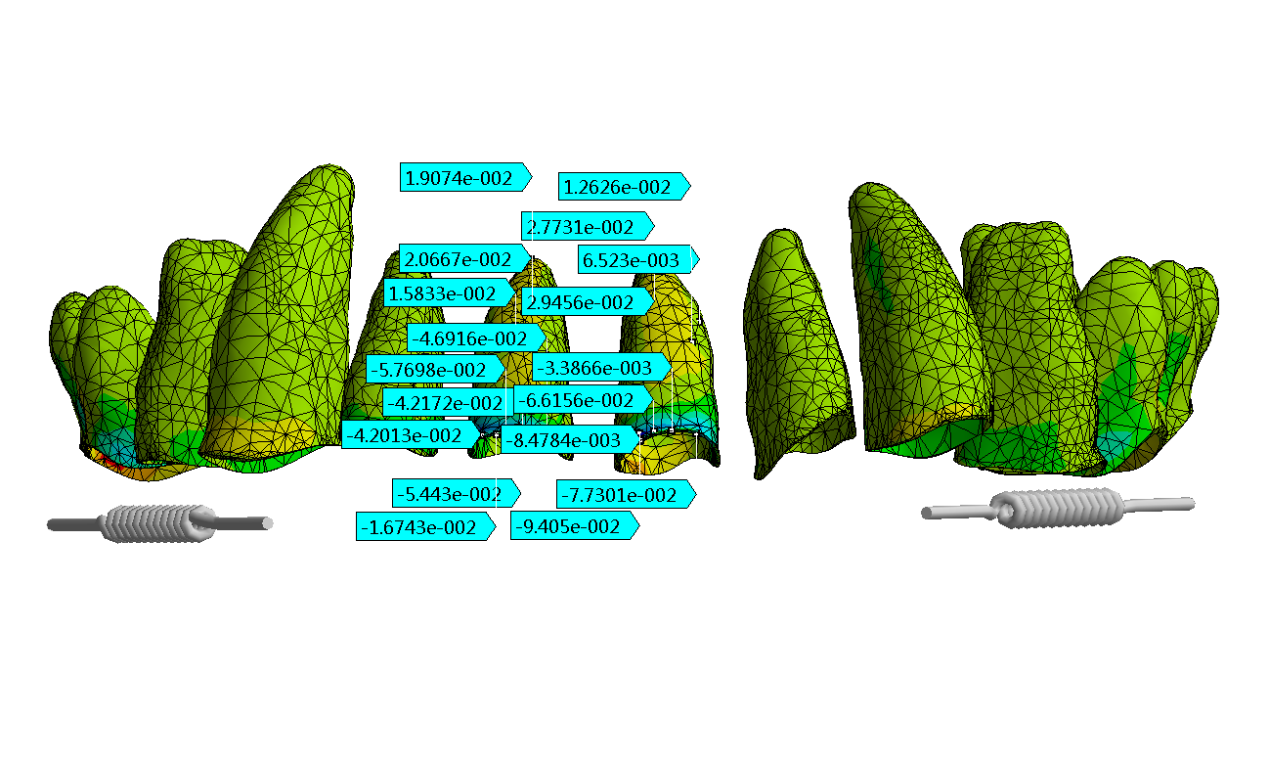

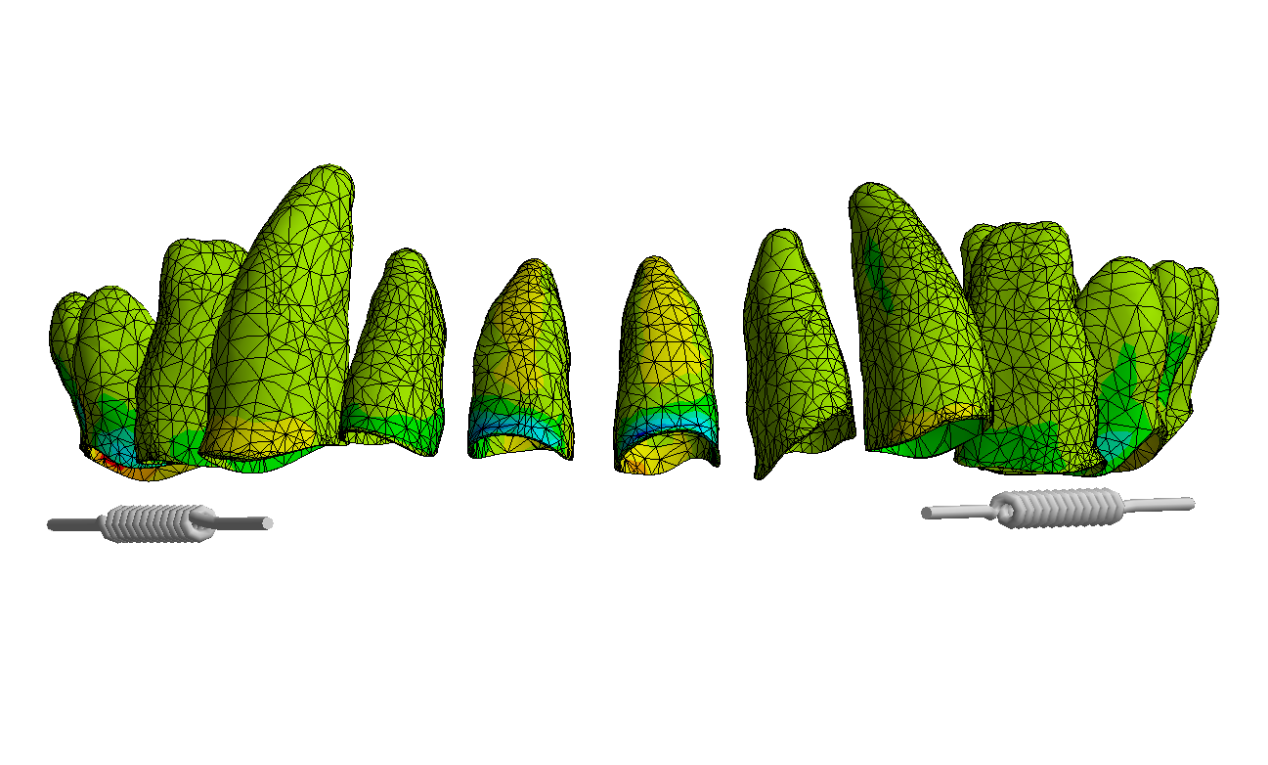


**Supplementary Figs S12: 3d group**

**4 group: Incisor displacement**


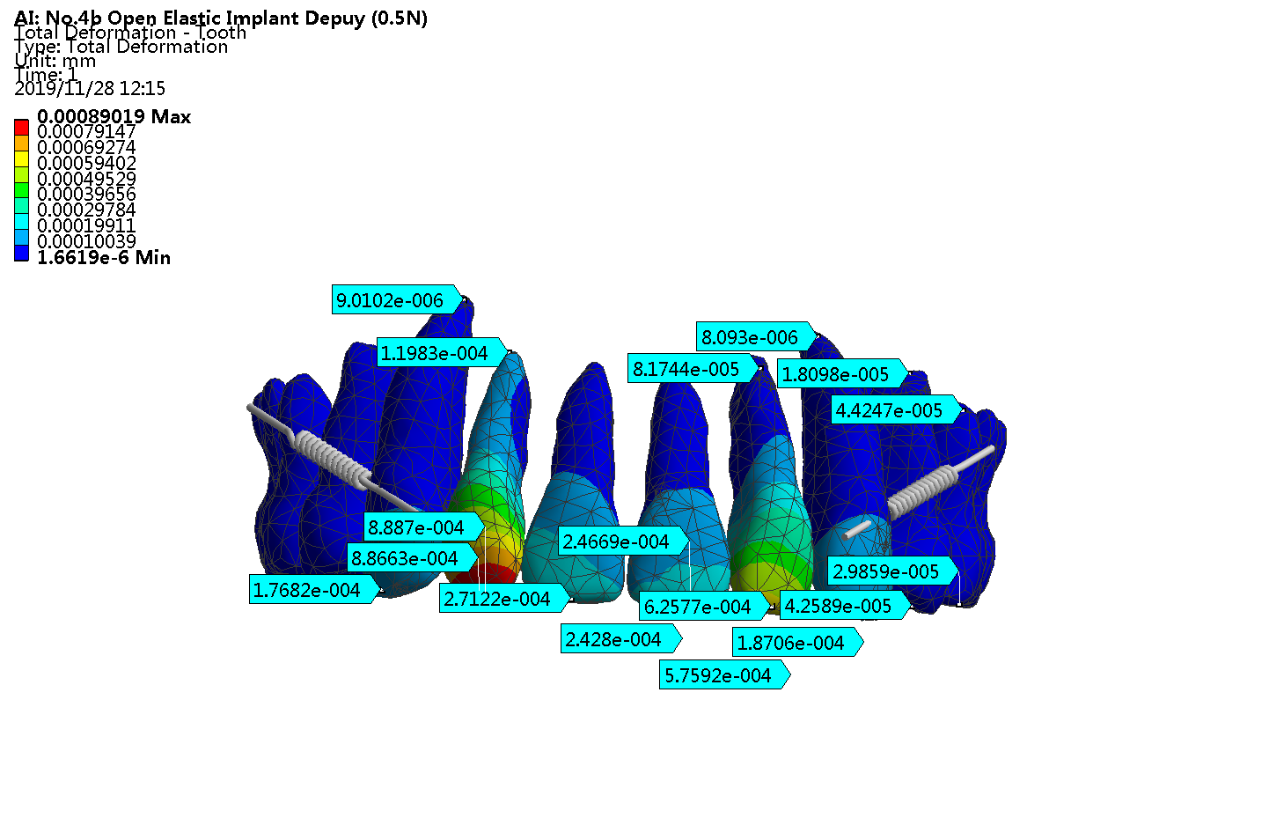

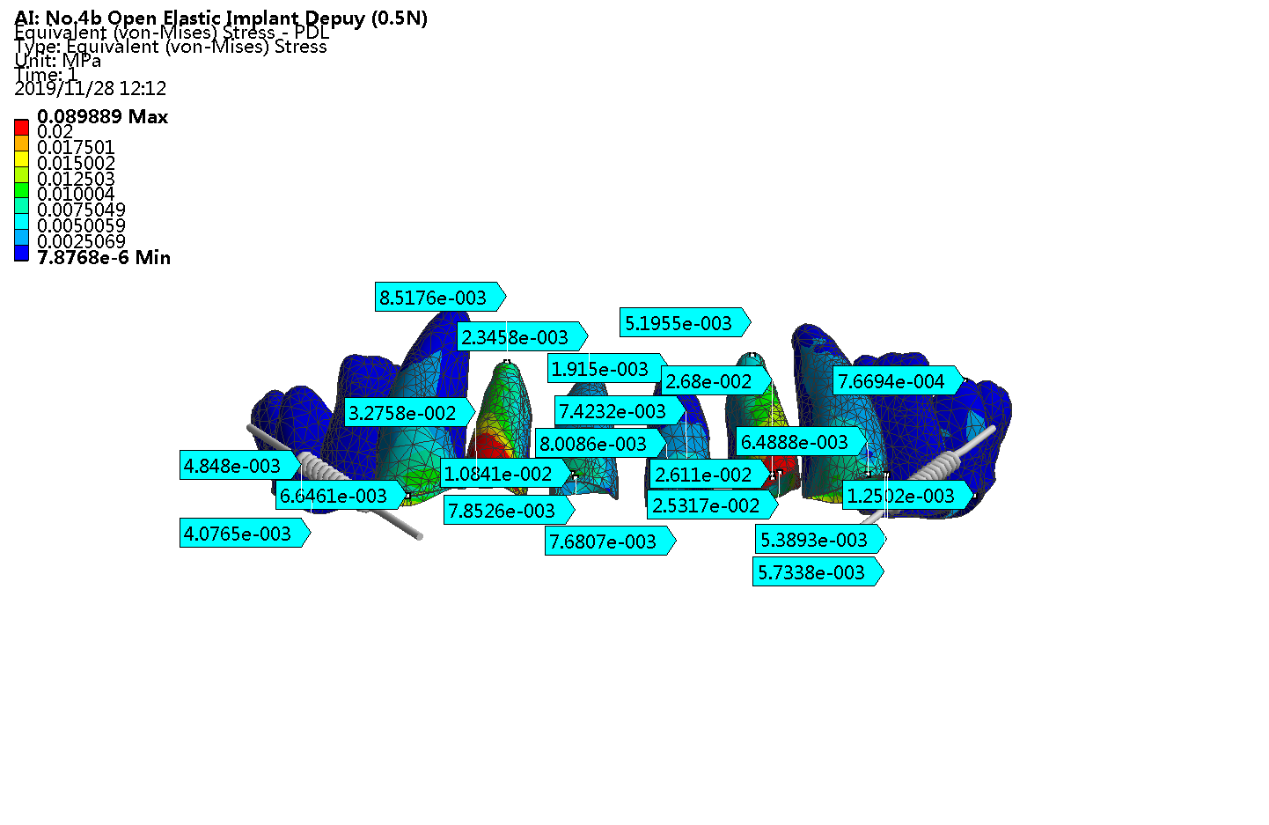

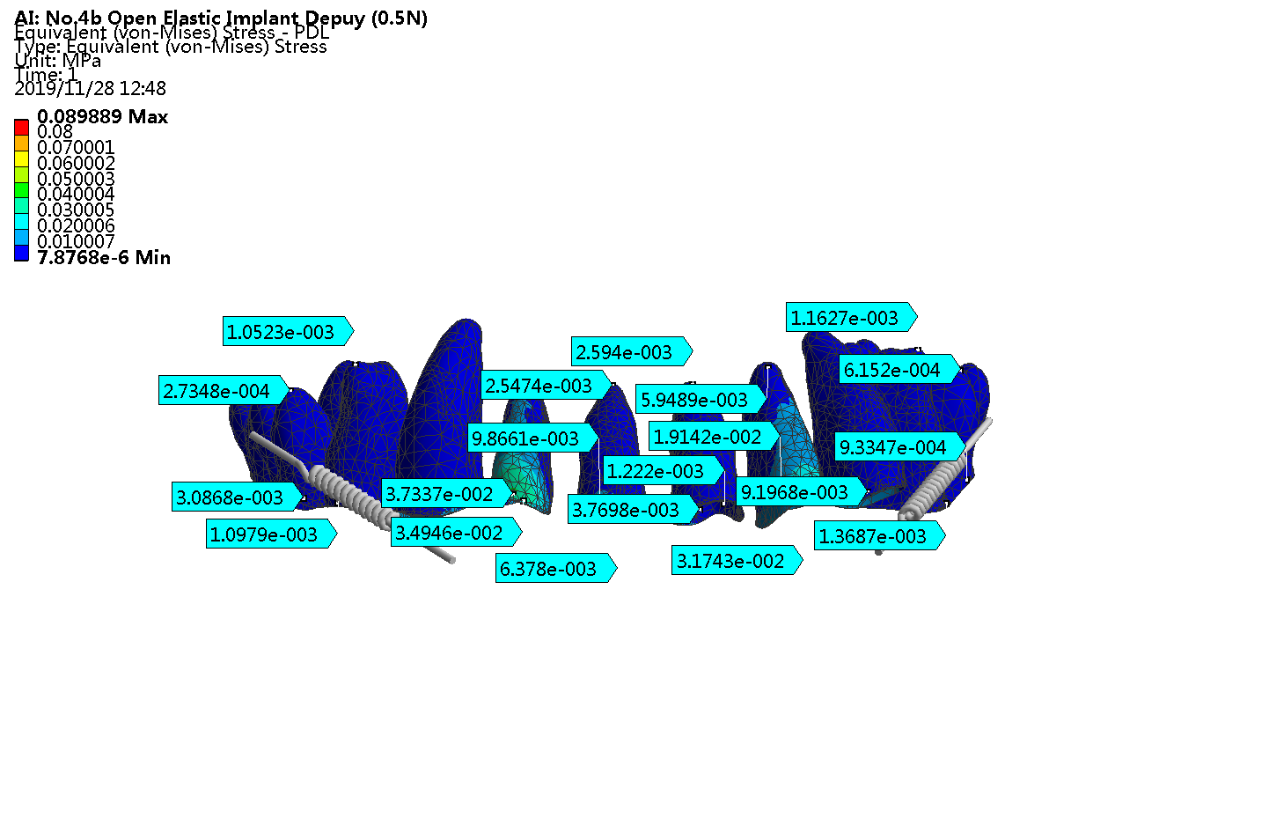

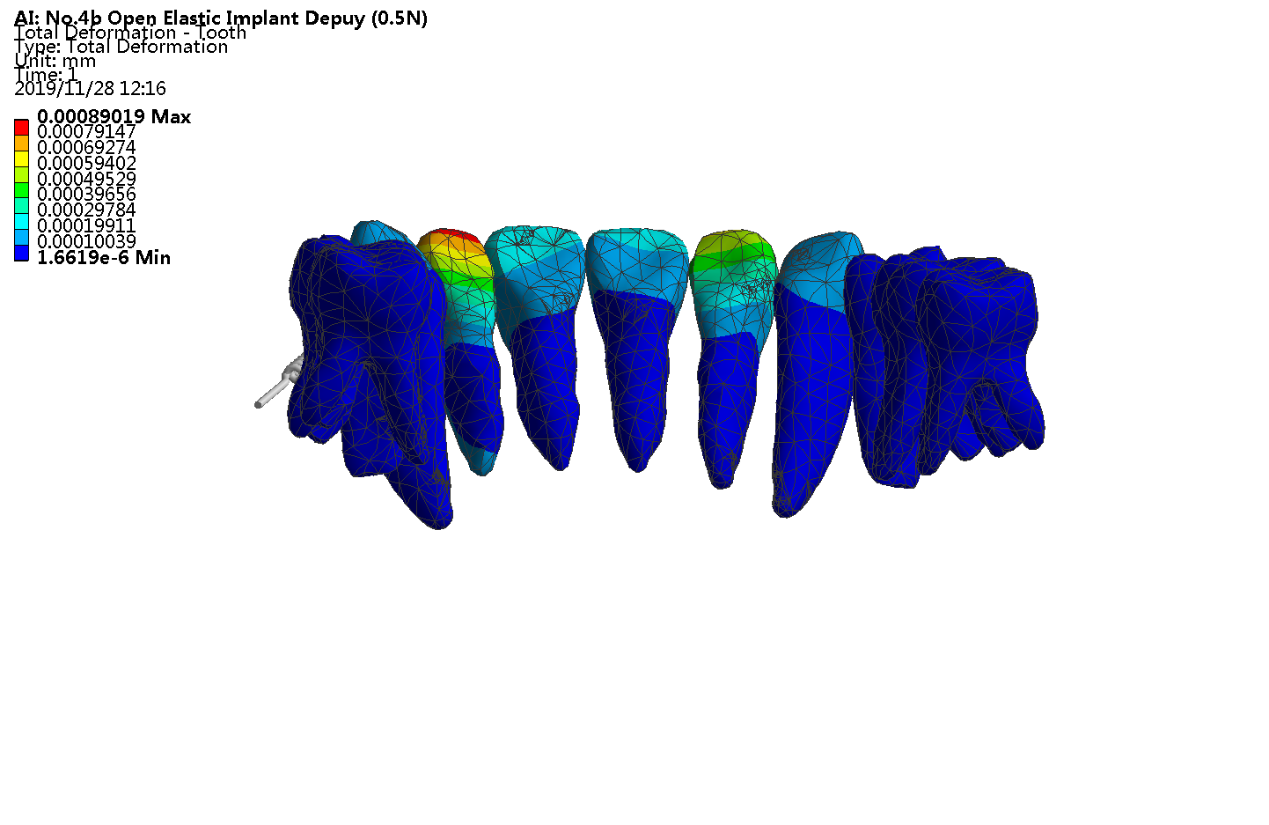

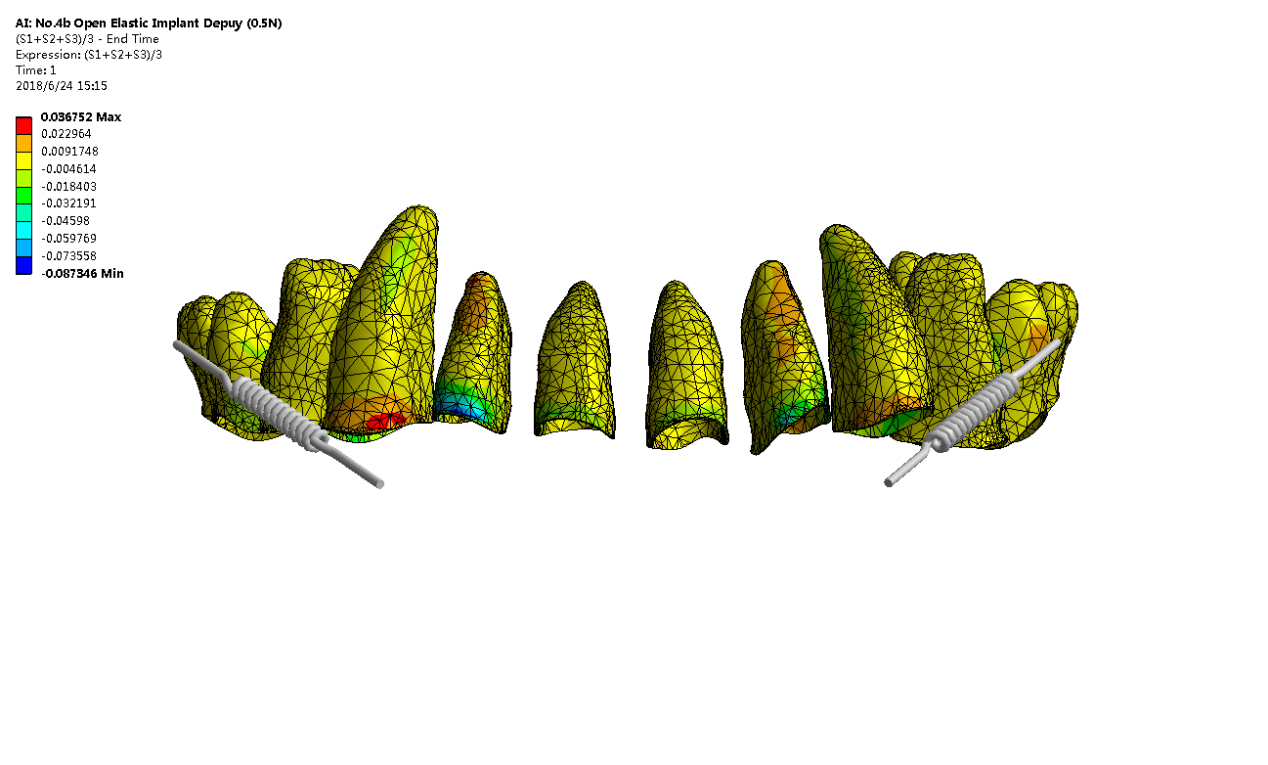

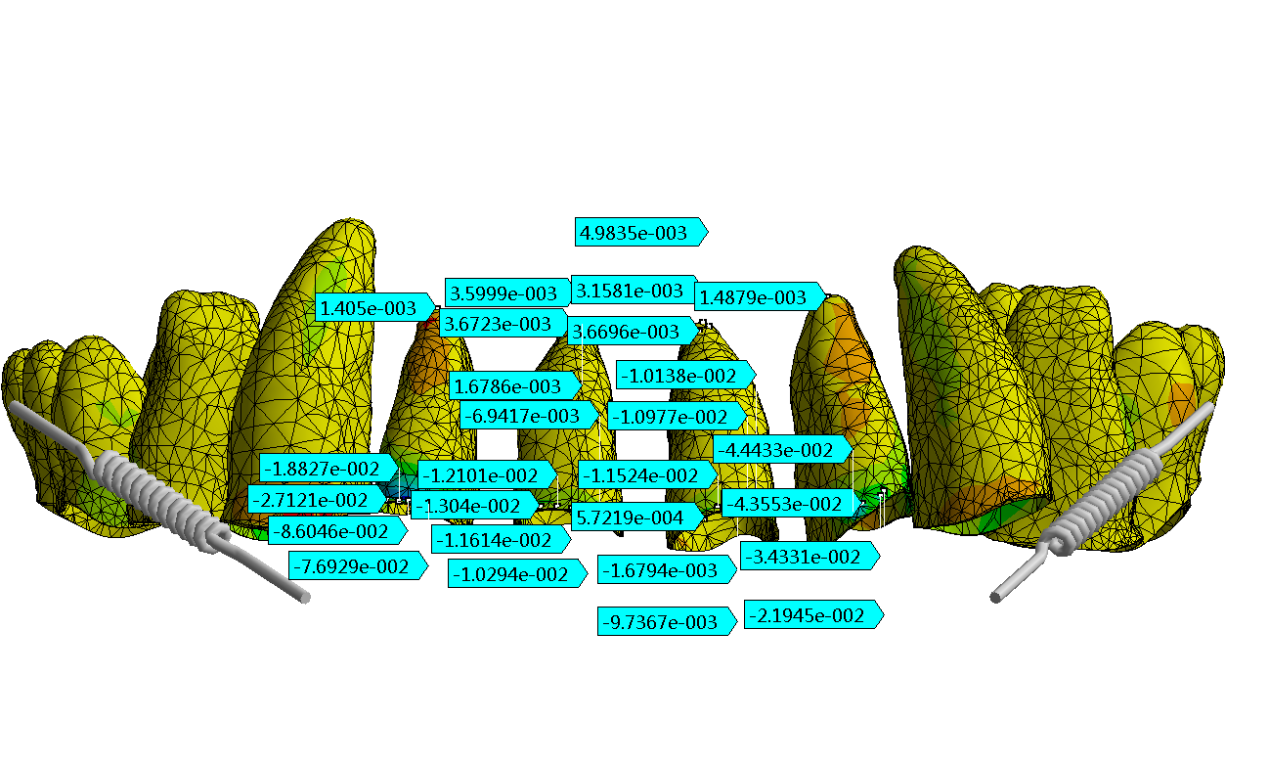

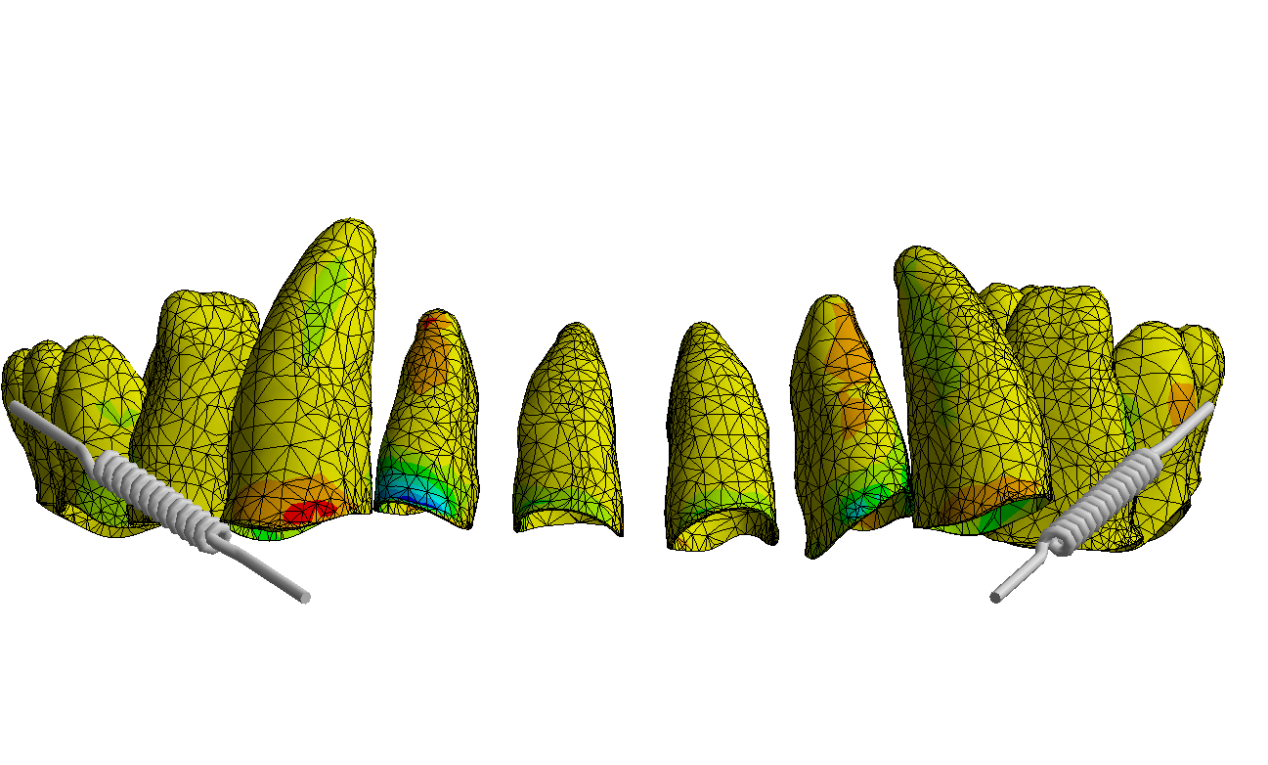


**Supplementary Figs S13: 4a group**


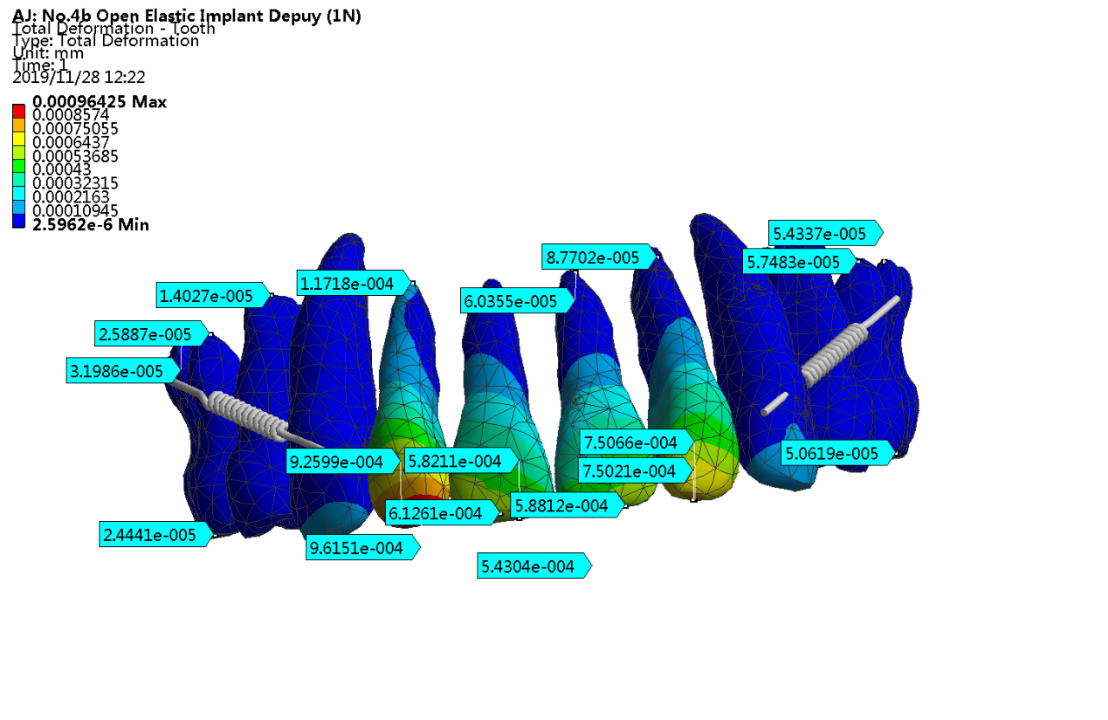

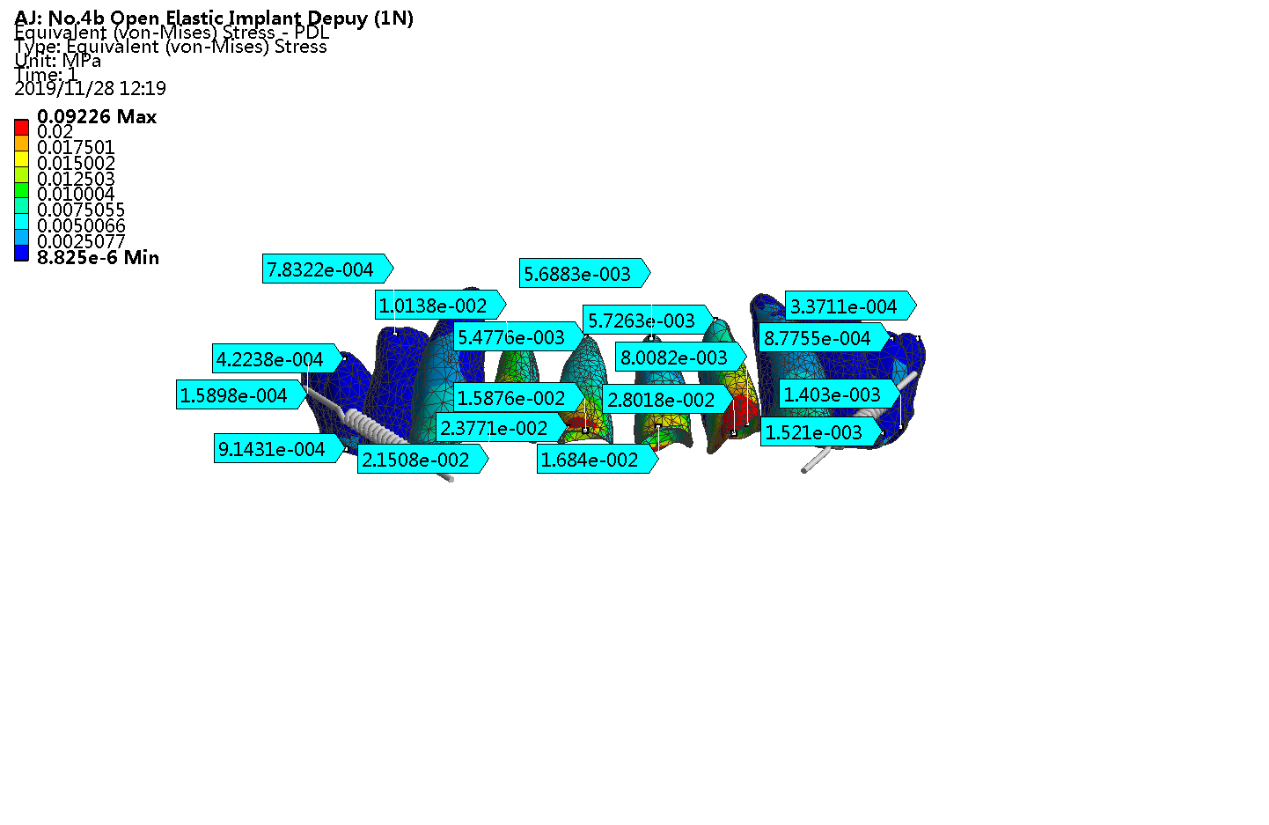

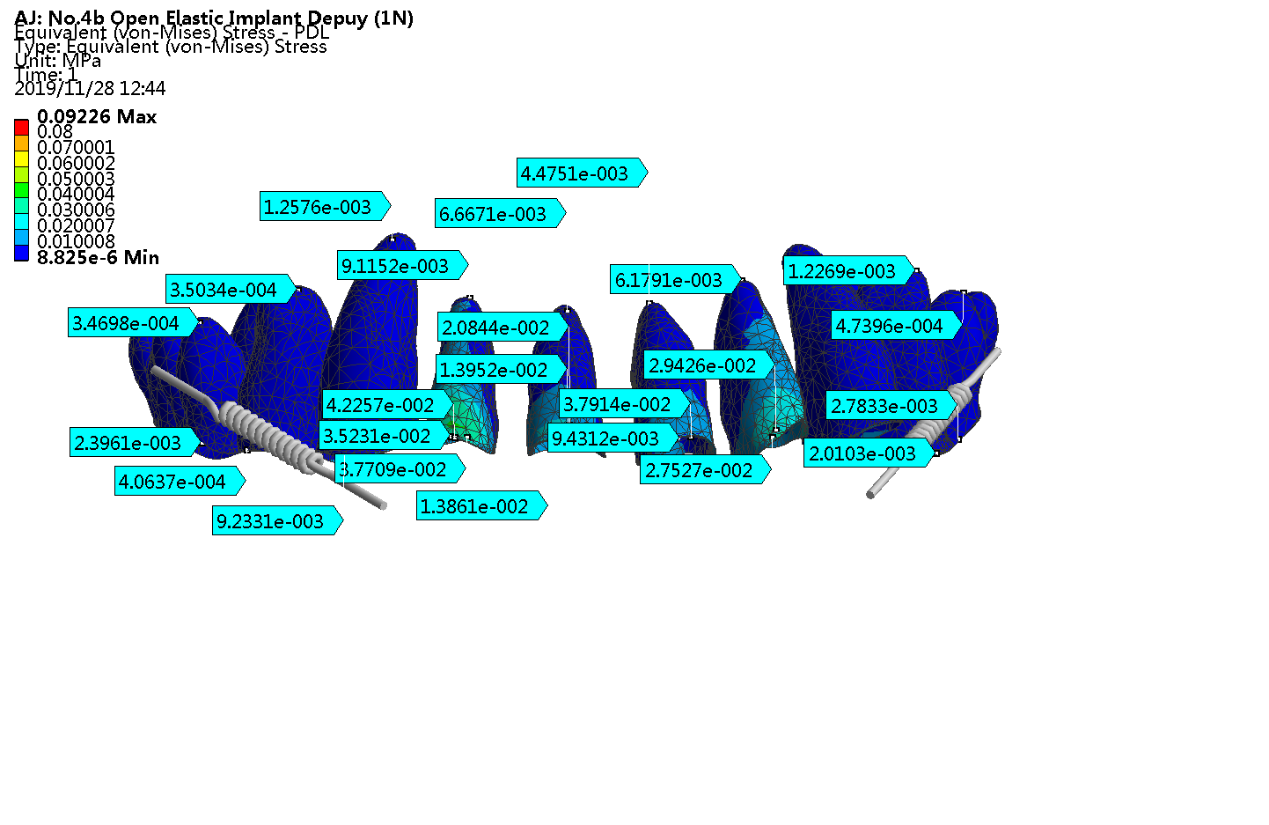

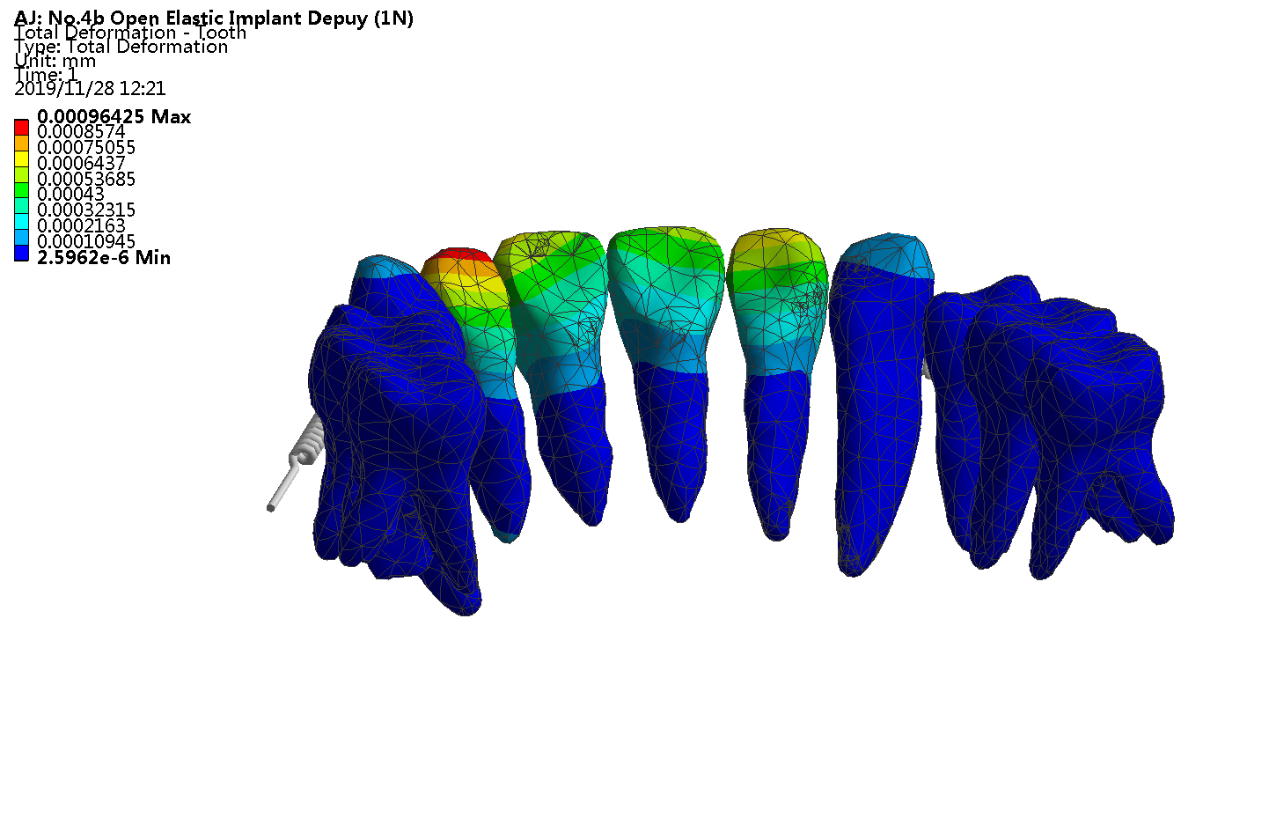

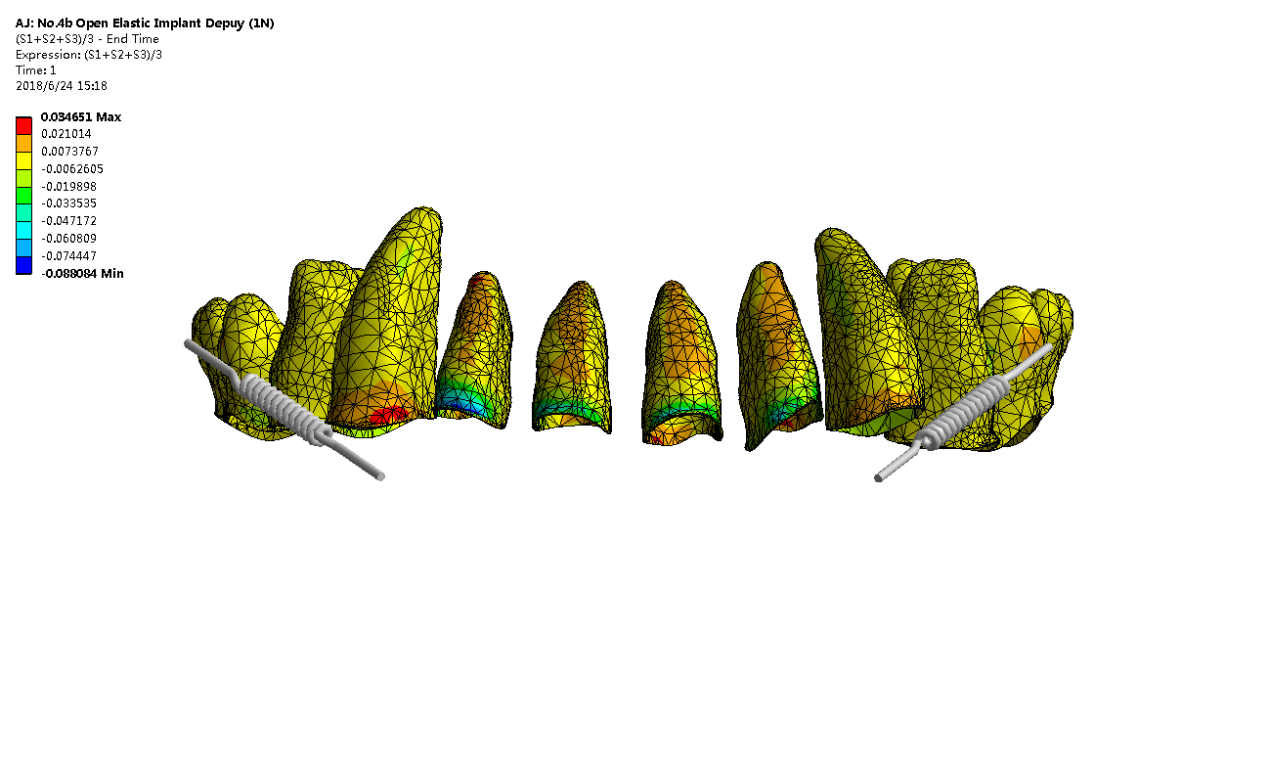

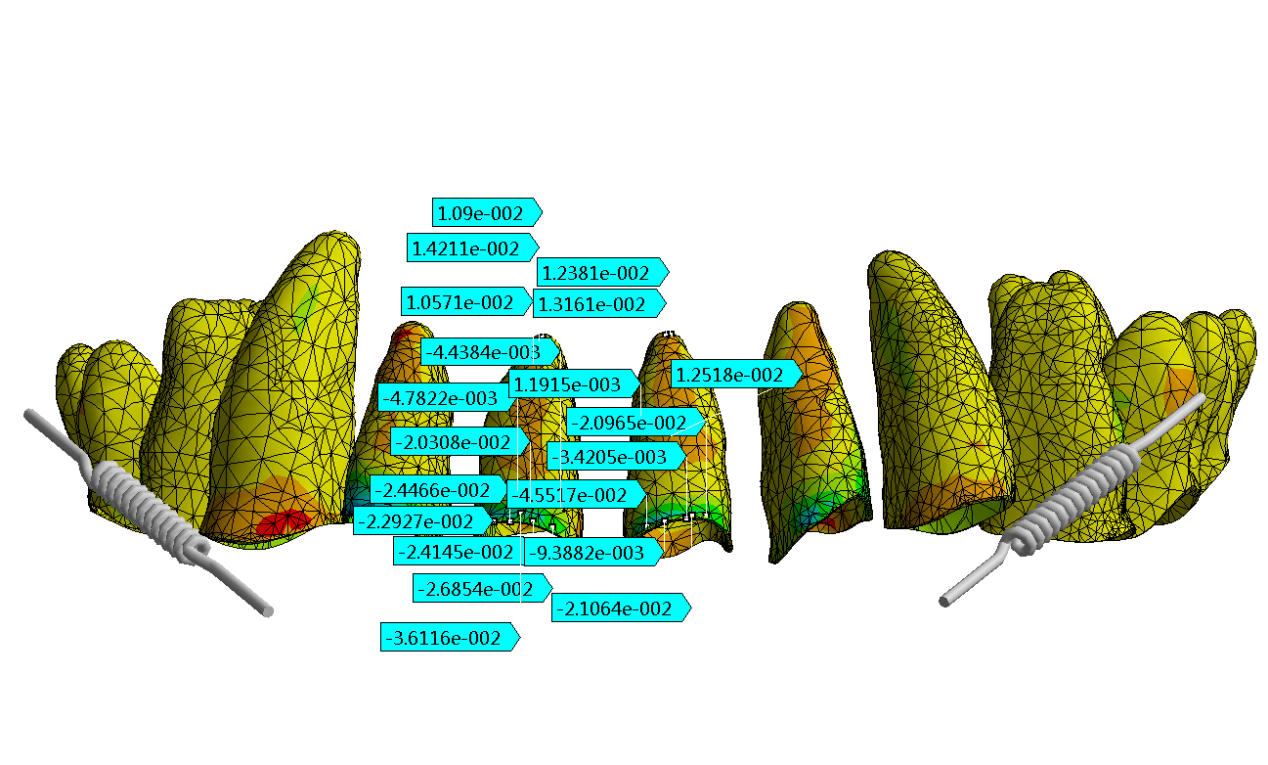

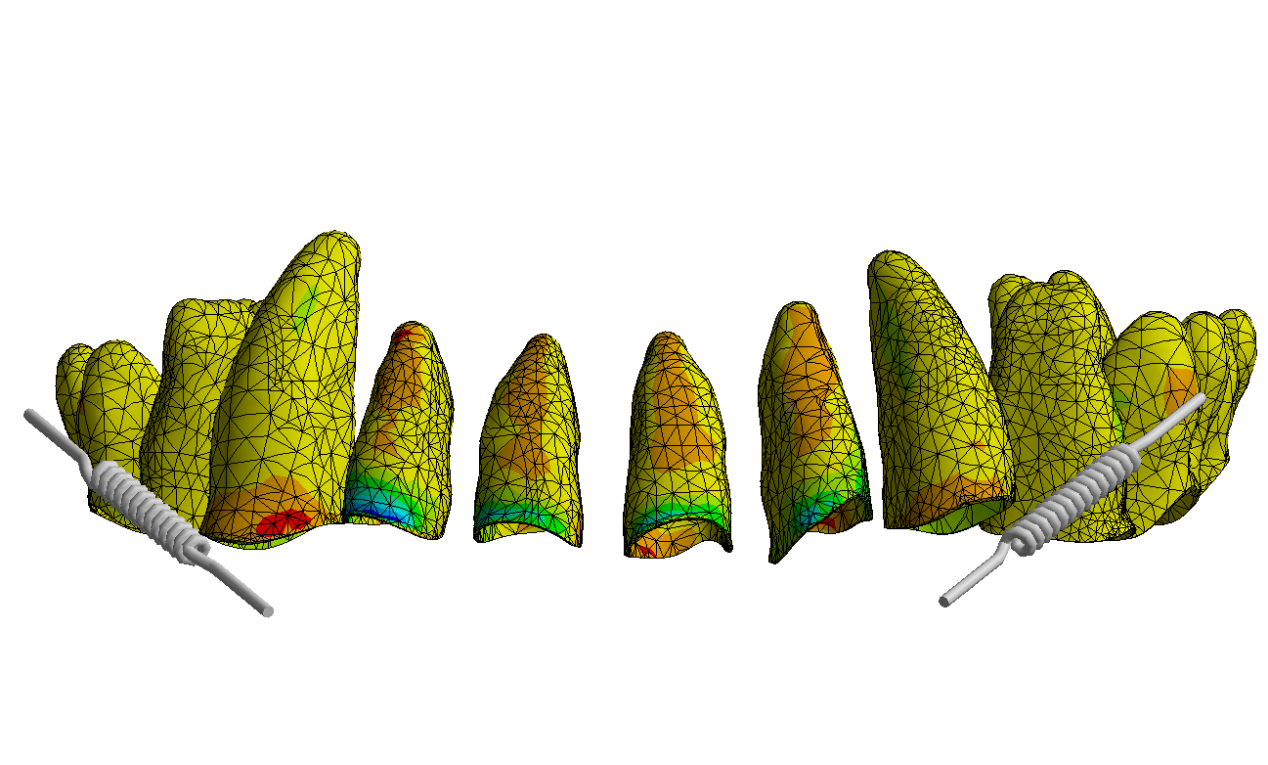

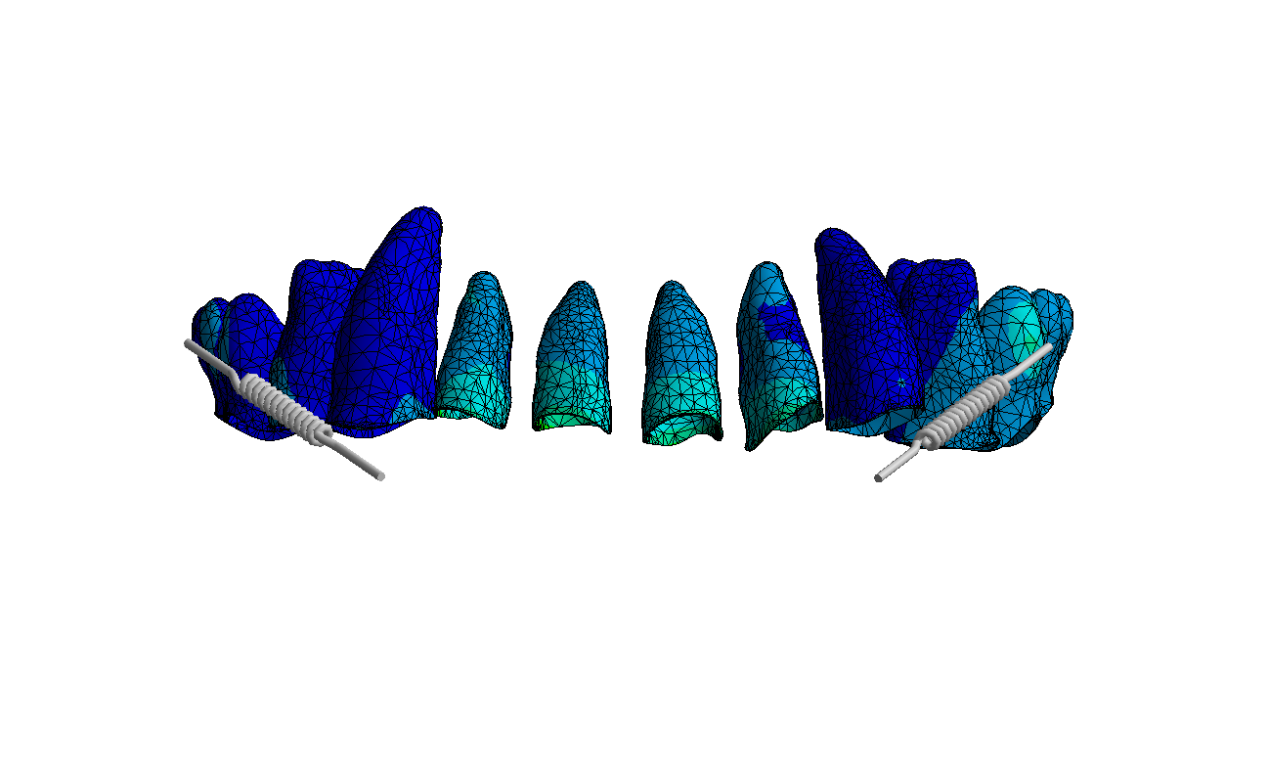


**Supplementary Figs S14: 4b group**

**Supplementary Figs S15: 4c group**

**Supplementary Figs S16: 4d group**

**Application of clinical cases**

**Supplementary Fig S34:** **Intraoral photos and radiographs**

**Supplementary Fig S35: self-made four-curvature Intraoral photos and radiographs**

**Supplementary Fig S36: end of the treatment Intraoral photos and** **radiographs**

**Supplementary Fig S37: Comparison of panoramic X-ray radiographs before, during and after treatment**
